# Advances in the Application of Low-Cost, Natural Materials, and Waste-Derived Catalysts for Catalytic Upgrading of Plastic and Biomass Pyrolysis Oil

**DOI:** 10.1021/acs.energyfuels.5c05623

**Published:** 2026-01-22

**Authors:** John Hughes, Abarasi Hart, Bikashbindu Das, Joseph Wood

**Affiliations:** † School of Chemical Engineering, 1724 University of Birmingham, Edgbaston, Birmingham B15 2TT, U.K.; ‡ Department of Chemical Engineering, 193160 Malaviya National Institute of Technology, Jaipur 302017, India

## Abstract

Unprecedented levels of population growth, urbanization, and industrialization have occurred in the 21st century, and with them has come an increase in demand for energy as well as a rise in the production of solid and plastic wastes. Heterogeneous catalysts designed from abundant and readily available solid waste provide a sustainable recycling strategy for industrial-scale applications. Nonetheless, the impact of purification techniques and raw material compositions may aid in tailoring the development of a robust catalyst for specific applications. Waste-derived catalysts have demonstrated capability in biofuel refining applications such as biodiesel synthesis, pyrolysis of lignocellulosic biomass, and waste plastic into oils. The first part of this review focuses on metal oxides that make different solid wastes viable for catalyst design and development, and the second and third parts cover the application of waste-derived catalysts in the catalytic upgrading of waste plastic and lignocellulosic biomass pyrolysis oils into fuels. For the industrial scalability of these waste-derived catalysts, their activity, stability, reusability, and regenerability in the context of upgrading oils derived from the pyrolysis of biomass and waste plastics were critically evaluated. Waste-derived heterogeneous catalysts were found to perform comparably to conventional industrial catalysts such as zeolite-based and hydrotreating (e.g., Ni-Mo/Al_2_O_3_) catalysts. Particularly, Red mud-derived catalyst has demonstrated cost-effectiveness and sustainable catalytic upgrading of bio-oil and waste plastic pyrolysis oil into fuel-range hydrocarbons (28–40 wt % gasoline, 35–50 wt % diesel fractions, and chlorine content less than 0.1 wt %). This research promotes the design and development of heterogeneous catalysts from industrial, municipal solid waste, biomass and agricultural residues, eggshells, seashells and bones, and e-waste by combining synthesis and purification methodologies to recover mixed metal oxide materials to bridge existing supply gaps. Consequently, their applications in the catalytic upgrading of oil produced from the pyrolysis of waste plastics and lignocellulosic biomasses into fuels offer economic, environmental, and energy security benefits.

## Graphical abstract

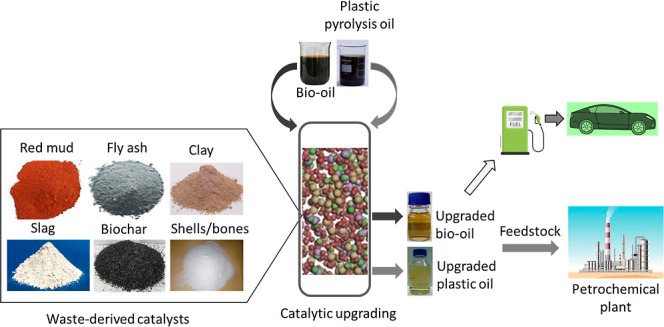

## 1 Introduction

Globally, about 150–180 million tons of Red mud (RM) are generated annually as a byproduct of the production of aluminum,^1^ with the total accumulated stockpile already exceeding 4 billion tons and projected to grow to 10 billion tons by 2050.^2^ In 2018, the annual coal fly ash (CFA) worldwide was around 500 million tons,^3^ while a projected 74.7 million metric tons of electronic waste (e-waste) will be generated by 2030.^4^ Furthermore, more than 400 million tons of iron and steel slag are generated annually worldwide.^5^ These solid waste materials contain a mixture of silica, calcium oxide (CaO), magnesium oxide (MgO), titania, alumina, and iron oxides, which makes them a potential resource for low-cost catalyst synthesis. In addition, poor treatment of these wastes can cause environmental degradation and pose serious health risks to inhabitants in surrounding areas. There is little attention paid to resource sustainability from the perspective of the circular economy model from industrial, e-waste, agricultural, and geological wastes.^6^ Industrial solid waste contains a complex array of transition, alkali, and rare earth metals as well as metal oxides (basic oxides, amphoteric oxides, and acidic oxides), which are known as active candidates for heterogeneous catalysts or support materials. Whereas electronic waste (e-waste), especially printed circuit boards (PCBs), contains high content of valuable metals like Cu and Sn, precious metals such as Ag, Au, and Pd; and rare earth elements that could be used as catalysts, such as Nd, La, and Ce.^4^ Hence, waste materials from mining and metal manufacturing, coal-fired power plants, agricultural residues, biomass and plastic pyrolysis, seashells and animal bones, and clays are being used to synthesize heterogeneous catalysts that are active, selective, and stable.^7^ However, improving the catalytic activity of the solid waste-derived materials would require target-based purification tailored to the composition of the starting material.

On the other hand, plastics have a broad spectrum of applications worldwide due to their affordability, durability, and simplicity of molding into various products, forms, and sizes. As a result, their production and usage have expanded and currently account for 8% of the global oil output.^8^ In 2021, the world’s plastics production reached 460 million tons (Mt), with 90.2% of the feedstock fossil-based and 44% for packaging applications.^9^ Whereas waste plastic has more than doubled, from 156 Mt in 2000 to 353 Mt in 2019.^10^ Macroplastic leakage is the result of mismanaged plastic waste due to inadequate collection and disposal. In 2019, about 22 Mt of plastic waste leaked into the environment, and 6.1 Mt leaked into rivers, lakes, and the ocean.^10^ Additionally, plastic waste is a global problem, as it is difficult to degrade naturally. About 80% of waste plastics accumulate in the natural environment.^11^ Furthermore, when waste plastics break down (e.g., photodegradation, thermal oxidation, hydrolysis, etc.) into tiny particles (i.e., microplastics), they become challenging and costly to clean up. However, recycled plastics accounted for about 8.3%, while biobased plastics represented about 1.5% of the world’s plastics production. Therefore, waste plastics are among the least recycled materials worldwide when compared to recycling steel (85%), aluminum (75%), paper (60%), and glass (50%).^12^ The use of plastics results in large plastic waste, which now limits landfill space and contaminates aquifers, rivers, and oceans. The impact of mechanical recycling of waste plastic on a global scale is limited to 9% and cannot handle hard-to-recycle plastics.^13,14^ As a result, mechanical recycling has a limited impact on an industrial and global scale. The recycling of waste plastic is crucial to the transition to a sustainable world. Thus, chemical recycling of plastic wastes via pyrolysis helps to reduce landfill, prevent the possible leakage of plastics into the environment, and consequently promote a plastic waste management strategy. Pyrolysis technology is a key strategy of chemical recycling and is a plausible and practical approach to recycling waste plastics on a large scale. Furthermore, chemical recycling via pyrolysis of waste plastics can contribute toward addressing Sustainable Development Goals (SDGs) such as SDG 7 (affordable and clean energy), SDG 9 (industry, innovation and infrastructure), SDG 11 (sustainable cities and communities), and SDG 13 (climate action). It has been shown that the composition of plastic feedstock affects pyrolysis oil quality in terms of hydrocarbon content [e.g., pyrolysis of polymers like polypropylene (PP), which produces highly branched olefins, and PE, which consists of largely linear compounds and wax] and contaminant concentration.^15,16^

Globally, over 181.5 billion tons of lignocellulose biomass waste [such as forestry residue (sawdust, wood chips, bark, etc.), agricultural residue (sugar cane bagasse, rice husk, corn stalk, etc.), and nonfood plant biomass (grasses), etc.] are generated annually.^17^ These are potential feedstocks for biofuel production, but only a small fraction are currently used to produce bioenergy. If waste plastic worldwide could be recycled using an efficient and successful recycling process like chemical recycling, it could save around 3.5 billion barrels of oil per year, equivalent to US$176 billion.^18^ These projections demonstrate the untapped potential of industrial and municipal solid waste (MSW), plastic, forestry, and agricultural waste for novel catalyst development and a sustainable energy future. Consequently, waste from ore processing, metal extraction plants, and plastics poses a serious challenge to the environment and represents a significant economic loss. Similarly, plastic waste disposal and emissions from fossil fuel combustion are major global challenges that have an impact on the environmental balance. Consequently, there is a possibility that oil reserves have peaked and are declining,^19^ with the added disadvantage of emitting large volumes of carbon dioxide, which contributes to environmental pollution and increases greenhouse gas emissions. From a pollution and energy crisis standpoint, increased energy demands can be supplemented by fuels produced by upgrading waste biomass and plastic pyrolysis oils (PPO). To achieve this, pyrolysis technology is the most widely used method to convert biomass and waste plastic into oils and valuable chemicals.^18,20,21^ It has been reported that pyrolysis of mixed plastic waste offers a lower climate change impact and better energy utilization compared to energy recovery from incineration.^22^ However, to efficiently convert waste plastic pyrolysis oil (WPPO) and bio-oil into sustainable fuels, valuable chemical building blocks, and other high-value products, a catalyst would play a pivotal role. As a result, the cost of a catalyst could be one of the most important factors for competitiveness. Therefore, achieving sustainability in both energy sectors and catalytic processes can be promoted by recycling industrial waste materials through waste-to-catalyst. In addition, recycling will contribute to the development of effective sustainable management strategies for industrial and MSW, plastic, and biomass waste. Thus, the conversion of oil from waste plastics and biomass utilizing solid waste-derived catalysts is beneficial not only for increasing fuel yields and reducing reliance on fossil fuels but also for minimizing environmental issues. Consequently, waste-derived heterogeneous catalyst applications not only address these environmental challenges, but they also contribute to resource recovery and energy sustainability, with the potential to reduce process costs and improve circularity.^23^

Catalytic pyrolysis technologies exhibit significant flexibility in recovering fuels from diverse waste plastics and biomasses (e.g., agricultural residues, wood chips, forest residues, energy crops, etc.). These waste plastics and biomasses are a resource worldwide, and recycling them through pyrolysis offers both economic and environmental benefits. The industrial scale processing of biomass pyrolysis and plastics pyrolysis oils face significant challenges related to feedstock variability, oil physicochemical properties, and process economics.^24–27^ As a result, the crude pyrolysis oil compositions are frequently subject to significant variation and complexity due to heterogeneities in the feedstock and pyrolysis conditions, making it challenging to develop and tailor a catalyst that can withstand these compositional variations. Specifically, WPPOs containing contaminants like oxygen, nitrogen, sulfur, chlorine, and other impurities,^28^ in addition to variations in feedstock composition, require catalytic upgrading to meet the stringent specifications of petrochemical plants or transport fuels.^29^ For biomass pyrolysis bio-oils, the high oxygenated compounds and water content greatly deactivate the conventional heterogeneous catalyst, adding to the economic burden of commercialization. Heterogeneous catalysts derived from abundant industrial solid waste have effectively improved the quality of bio-oil and WPPO obtained by the pyrolysis technology. This is because these industrial waste materials contain common metal oxides such as SiO_2_, Al_2_O_3_, TiO_2_, and Fe_2_O_3,_ which possess an intrinsic porous structure and catalytic activity. A review on the application of industrial solid waste catalyst to increase the yield and the quality of bio-oil obtained from biomass by pyrolysis has been published with a focus on conversion mechanism, pyrolysis characteristics, and product composition.^30^ Consequently, the role of waste-derived catalysts in increasing process efficiency, minimizing the formation of undesirable substances, improving product yield, and quality from plastic pyrolysis into oil has been highlighted in the review on selection of relevant catalysts,^31^ factors affecting the catalytic pyrolysis of waste plastic,^32^ and properties of plastic feedstock and different catalysts.^14^ These reviews focus on the application of waste-derived catalysts in improving the quality of liquid products from pyrolysis. However, this work focuses on in-depth evaluation of the suitability of catalysts derived from industrial waste, their application, and scalability in catalytic upgrading of pyrolysis oil from waste plastics and biomasses into sustainable fuels. This is because strict specifications will be necessary to ensure compatibility with existing infrastructure, depending on whether the upgraded pyrolysis oil will eventually be utilized for petrochemicals or blended into fuels.

Most of the published reviews focus on the application of natural clay, biochar, and industrial waste-derived catalysts in the pyrolysis of waste plastic into fuels; biochar-based catalysts,^29^ RM,^33^ clay materials,^34^ and industrial solid waste [RM, CFA, copper slag, blast furnace slag (BFS), coal gangue, and aluminum dross].^35^ A review on the advances of the effect of industrial solid waste as a catalyst on the quality of bio-oil during the process of biomass pyrolysis has been reported.^30^ These reviews are on the effect of operating parameters on the yield and quality of bio-oil and mechanisms of catalytic upgrading of bio-oil. There is a limited comprehensive review on the application of low-cost, natural materials and waste-derived catalysts in the upgrading of bio-oil and PPO. Hence, this review addresses critical gaps in the literature by presenting holistic strategies used to produce catalysts from different geological and solid waste materials and evaluating their performance and practical applications of these waste-derived catalysts for bio-oil and PPO upgrading into fuel-range hydrocarbons. It highlights crucial environmental and economic issues of industrial scale production of waste-derived catalysts, provides a roadmap for industrial applications, and elucidates diverse mechanisms for catalyst deactivation. Also, the challenges associated with the transition from laboratory synthesis to industrial scale production of heterogeneous catalysts from waste materials and their full utilization in sustainable fuel production are highlighted. The first aspect of the review focuses on method and purification techniques used to synthesize robust and low-cost catalysts from different industrial, clay, and mining waste materials. The waste-derived catalyst activity in the production of fuel-range hydrocarbons from pyrolysis oil from waste plastic and biomass is covered in the third and fourth sections. Catalytic upgrading of bio-oil is mostly aimed at removing oxygen to produce liquid fuel-range hydrocarbons, while that of WPPO focuses on contaminant removal and C-C cracking. Thus, developing more efficient and cost-effective catalysts for upgrading pyrolysis oils from waste plastics and biomass is crucial to commercialization.^36^ The cost of catalysts contributes greatly to the overall process economics due to their influence on energy requirements, reaction pathways and kinetics, product selectivity, and yields. Thus, a cheap catalyst that is active, selective, and stable is an ideal candidate that would improve the process economy and competitiveness with fossil-fuel counterparts. However, the low-cost catalyst must be active and can withstand high contaminants in plastic oils and consume less hydrogen in the case of hydrodeoxygenation (HDO) and hydrogenation of bio-oil.

## 2 Waste-Derived Catalysts

### 2.1 Context and Motivation

Catalytic upgrading is essential to resolve the challenges associated with PPOs and biomass pyrolysis bio-oils, but the high cost of conventional catalysts has limited industrial scale-up.^37^ This has driven a surge in research into using low-cost waste materials as alternative catalysts. Industrial residues like RM and steel slag, agricultural byproducts like biomass ash, and even MSW ashes are rich in catalytically active metal oxides (e.g., Fe_2_O_3_, CaO, Al_2_O_3_, and SiO_2_) and have shown remarkable performance.^30,38,39^ The effectiveness of waste-derived catalysts stems from their intrinsic properties. As a byproduct of steel manufacturing, the properties of BFS include bulk density (0.69 to 1.43 g cm^–3^), true density (2.8 to 3.1 g cm^–3^), chemical composition of primary oxides (SiO_2_, Al_2_O_3_, MgO, and CaO), the CaO/SiO_2_ ratio determine the basicity (pH 8–10), high thermal stability, specific surface area (400–600 m^2^ kg^–1^), and particle size (0.5–4.26 mm). CFA is a heterogeneous material with primary components (oxides of silicon, aluminum, calcium, and iron) generated from coal combustion. Its particle size ranges from less than 0.1 to 200 μm, specific gravity (1.6–3.1), bulk density (0.6–3 g cm^–3^), classified into acidic ash (pH 1.2 up to 7), mildly alkaline ash (pH 8–9), and strongly alkaline ash (pH 11–13), high thermal stability, porous structure, specific surface area (2 to 40 m^2^ g^–1^), and possesses active sites that can promote catalytic activity.^40^ RM is the byproduct of bauxite ore refining into alumina for aluminum production. The color of RM is due to its high content of iron oxide (Fe_2_O_3_), high alkalinity (pH 11–13.2), coarse sand and fine particle with particle size of 90% less than 75 μm, porous structure with a density of 2.7–2.9 g cm^–3^, specific surface area is about 64.1–186.9 m^2^ g^–1^, and high thermal stability with melting point (between 1200 and 1250 °C).^41^ The pore structure and volume of these waste materials can be improved through acid or alkali treatments, which increases its surface area, removing impurities like sulfur and others, and exposing more active sites for enhanced catalytic activity. Typical sources of low-cost catalysts include biochar and MSW ash. Biochar is a carbon-rich solid material obtained as a byproduct of biomass pyrolysis into bio-oil or gasification into syngas. Depending on the feedstock and pyrolysis temperature, the produced biochar possesses an interconnected porous structure, high specific surface area (3–152 m^2^ g^–1^), high carbon content (35–95%), pH (3.1–12.3), bulk density (0.12–0.32 g cm^–3^), thermal stability depends on its composition, and surface functional groups, such as −COOH, −C=O, −OH, etc. (provide active sites for reactions).^42^ MSW ash is composed primarily of oxides like silica, alumina, and calcium, high metal content (3–15 wt %.), pH (10–12), density (200–500 kg m^–3^), and specific surface area (2–35 m^2^ g^–1^). Postproduction treatments such as acid or alkali activation can significantly increase the surface area and enhance pore structure and catalytic activity. These properties make biochar and MSW ash good candidates for low-cost carbon-based catalysts. Table 1 shows the catalytically active components in waste-derived catalysts, their catalytic functionalities and applications, and common sources.

**Table 1:** Primary Active Components in Waste-Derived Catalysts: Physicochemical Properties, Catalytic Functions, and Common Sources

active component	key functions and impact	common waste sources (**Ind**: industrial/**Bio**: biogenic)
Group 1: Primary Base Catalysts (Deacidification and Upgrading)		
calcium oxide (CaO)	excels at deacidification and deoxygenation^43^	Bio: eggshells, seashells, bones
	boosts hydrocarbon yields^44^	Ind: paper-mill lime mud, marble sludge^46,47^
	biogenic forms resist deactivation^45^	
magnesium oxide (MgO)	mitigates coke formation^7^	Ind: dolomite mining waste, magnesium slag
	synergistic with CaO for oil upgrading^48^	Bio: certain clamshells^48–50^
alkali metals (K_2_O, Na_2_O)	highly effective for deacidification^51^	Bio: banana peels, sugar cane bagasse
	directs selectivity to valuable chemicals^52^	Ind: paper mills waste^53–56^
Group 2: Redox and Acidic Metal Oxides (Cracking, Reforming, and Contaminant Removal)		
iron(III) Oxide (Fe_2_O_3_)	high tar conversion (>94%)^57^	Ind: red mud, steel slag, coal fly ash^38,57,60,61^
	boosts valuable aromatics^58^	
	some forms are magnetic for easy recovery^59^	
zinc oxide (ZnO)	excels at desulphurization^62^	Ind: spent synthesis catalysts.^64^ Waste tire char (closed-loop system)^65^
	improves bio-oil stability^63^	
nickel (Ni/NiO)	essential for hydrodeoxygenation, hydrodesulphurisation, and steam/dry reforming. Produces high gasoline-range fractions^66,67^	Ind: spent petroleum hydrotreating catalysts^68^
		nickel-plating sludge^69^
		active metal sites onto red muds or slag derived zeolites^70,71^
titanium dioxide (TiO_2_)/ilmenite (FeTiO_3_)	promotes polymer cracking to fuel^72^	low-cost ilmenite ore^72,73^
	ilmenite (FeTiO_3_) acts as an oxygen carrier^73^	Bio: from orange peel waste^74^
other metal oxides	CuO: selective hydrogenation oxygenate compound^75^	Ind: CuO: electronic waste (printed circuit boards, PCBs)^80^
	MnO_*x*_: redox catalyst from spent batteries^76^	Ind: MnO_x_: spent Lithium-ion batteries^81,82^
	CeO_2_: redox promoter with oxygen storage^77^	Ind: CeO_2_: spent industrial polishing slurries^83,84^
	V_2_O_5_: promotes valuable phenols/furans.^78,79^	Ind: V_2_O_5_: spent vehicle/power plant catalysts^85,86^
Group 3: Frameworks and Supports (Shape Selectivity and Stability)		
aluminum oxide (Al_2_O_3_)	provides Lewis acid sites for cracking.^87^ Critical, stable support for active metals^88,89^	Ind: red mud, coal fly ash.^90,91^ Recycled: spent fluid catalytic cracking (FCC) catalysts^92–94^
silicon dioxide (SiO_2_)	thermally stable platform for active metals. Al_2_O_3_ creates strong acid sites^95,96^	Bio: rice husk ash (high purity)^97^
		Ind: coal fly ash spent FCC catalysts^98^
aluminosilicates (zeolites)	micropores control product distribution to favor aromatics. Waste-derived versions offer improved stability^99^	Ind: spent FCC catalysts. Synthesized from various industrial slags (steel, blast furnace, etc.)^27,71,100,101^
Group 4: Specialised Function Catalysts		
phosphates (hydroxyapatite)	selectively produces aromatics from plastic (Sun et al., 2020). Stable for biodiesel production^102^	Ind: sewage sludge and ash^104,105^
		Bio: animal bone char^103^
sulphided phases (MoS_2_, NiWS)	dual function hydrodeoxygenation and hydrodesulphurization.^106^ Highly resistant to coking	Ind: spent hydrotreating catalysts from petroleum refineries^107^

### 2.2 Waste Streams as Catalyst Precursors

Waste-derived solids are emerging as a versatile, low-cost platform for catalytic upgrading of PPOs and crude bio-oils. Because their mineralogy and carbon structures were created in high-temperature industrial or natural processes, they already tolerate the 400–800 °C conditions typical of fast-pyrolysis reactors and secondary crackers. Valorizing such “waste” as catalysts therefore offers a double dividend: it displaces virgin catalyst manufacture and converts potential environmental liabilities into valuable refinery inputs.

The catalytic effectiveness of waste-derived materials arises from several key physicochemical properties. These include high surface area and developed porosity for reactant access,^108^ acid–base character, with both Lewis and Brønsted sites available for cracking, isomerization, and cyclization reactions,^109^ redox-active metal oxides (particularly Fe_2_O_3_, MnO_x_, and CeO_2_) that facilitate oxygen transfer and hydrogenation,^110^ and the inherent thermal and mechanical stability from their high-temperature origins. The morphology and particle size can be controlled through post-treatment. Understanding these properties enables rational catalyst modification and design for specific upgrading reactions. The flowchart for the synthesis and development of heterogeneous catalysts from different waste materials is shown in Figure 1. The methods of synthesizing catalysts from waste materials depend on their source, nature, and composition. They include thermochemical processes like pyrolysis [design of carbon-based catalysts like activated carbon (AC) and biochar],^29^ hydrothermal treatment (converts materials fly ash, slags, and clays into catalytically active form),^111^ calcination (converts waste animal bones and shells into metal oxides),^112,113^ and precipitation (extract dissolved metal ions from a solution by adding a precipitating agent). Consequently, structure-directing agents such as tetrapropylammonium bromide (TPAB), cetyltrimethyl ammonium bromide, etc. can be added during the synthesis stage to improve textural properties, such as pore structure and surface area, in the catalyst synthesis from fly ash, industrial solid waste, and clay materials. To improve the purity of heterogeneous catalysts synthesized from industrial waste and clay materials, the following purification strategies can be implemented: pretreatment (e.g., thermal treatment, acid washing, hydrothermal treatment with alkaline solution, etc.), optimization of synthesis conditions (such as time, temperature, etc.), and post-treatment (e.g., acid treatment, ion exchange, solvent washing, etc.).^111^ The associated costs for the synthesis of heterogeneous catalysts from waste materials include collection and transportation, solvent and chemical, and energy costs.

**Figure 1:**
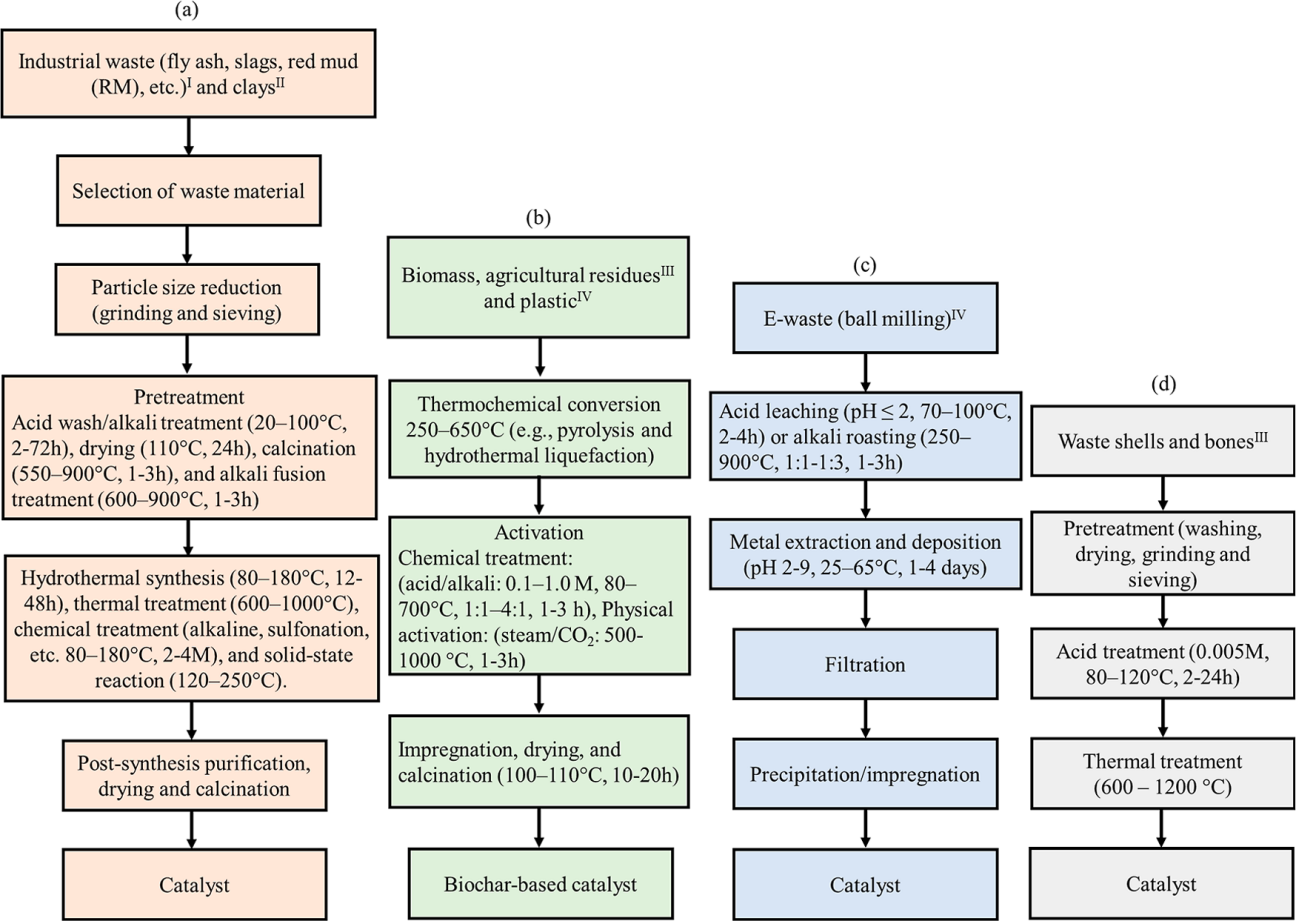
Flowchart for waste-derived catalyst synthesis: (a) industrial waste, (b) biomass and agricultural residues, (c) e-waste, and (d) shells and bones. Note: waste sources (Industrial sources^I^, Geological sources^II^, Biological sources^III^, and Municipal sources^IV^).

#### 2.2.1 Industrial and Metallurgical Waste-Derived Catalysts

Solid waste generation is expected to reach 11 million tons per day by 2100 based on current trends.^114^ Thus, the industrial and metallurgical sectors would generate sustainable quantities of solid byproducts that are rich in catalytically active elements. By unification of these streams, they can be categorized by their dominant active phases: metal oxides and aluminosilicates. Waste materials such as slags, dusts, CFA, and muds typically contain high concentrations of metal oxides, aluminosilicates, and alkaline compounds, making them inherently suitable for the high temperature, demanding environment of pyrolysis oil upgrading. Their use as catalysts is a prime example of industrial symbiosis, offering a pathway to convert hazardous or low-value waste into functional materials. For instance, BFS is composed largely of SiO_2_, Al_2_O_3_, CaO, and MgO, and traces of Fe_2_O_3_, MnO, Na_2_O, K_2_O, TiO_2_, P_2_O_5_, and Cr_2_O_3_, while CFA is composed of silica (SiO_2_), alumina (Al_2_O_3_), iron oxide (Fe_2_O_3_), and sometimes CaO. Bayer RM is a highly alkaline slurry (pH 11.9–13.0) rich in catalytically active oxides like Fe_2_O_3_ (31–47 wt %), Al_2_O_3_ (11–23 wt %), TiO_2_ (trace–25%), SiO_2_ (3–50%), and CaO (3–21 wt %) based on the bauxite ore.^60,61^ The methodology for catalyst development from these waste materials is shown in Figure 1a. Simple thermal activation via acid washing and calcination (∼700–750 °C) converts its native goethite into porous hematite, exposing a high density of redox-active and basic sites and increasing its surface area.^115^ Its utility as a catalyst support has been clearly demonstrated in hydrotreating. For example, a nickel-supported RM catalyst (Ni/RM) was more effective for deoxygenating guaiacol (a lignin model compound) than a commercial Ni/SiO_2_-Al_2_O_3_ catalyst. Crucially, the Ni/RM catalyst moderated hydrocracking, which preserved the aromatic ring structure, and produced less coke, resulting in a higher liquid yield of valuable chemicals like cyclohexane (38.8%) and benzene, toluene and xylenes (BTX) components.^116^ This catalytic activity is also robust; in the fast pyrolysis of pinyon juniper, the catalyst maintained stable deoxygenation performance even when the temperature was lowered from 450 to 400 °C, suggesting a wider and more flexible operating window for industrial processes.^117^

A key strategy for maximizing RM’s effectiveness is its use in dual-bed catalytic systems. By placing RM as a robust, first-stage cracking catalyst ahead of a more sensitive zeolite like HZSM-5, it can function as a “guard bed”. This approach boosted BTX yield from rape-straw by over 70% while simultaneously cutting coke deposits on the expensive zeolite by 37%, dramatically improving both yield and catalyst lifetime.^115^ From a practical standpoint, a major advantage of RM is its ease of recovery. During thermochemical treatment, its high iron content is converted into magnetic phases (e.g., Fe_3_O_4_), which allows for magnetic separation and recovery from char. This enables straightforward regeneration and reuse, which is crucial for process economics, and its effectiveness over multiple cycles has been demonstrated.^59,118^ Beyond biomass, the RM is a versatile catalyst for waste plastic valorization. It effectively cracks simple PP to increase valuable olefin content,^119^ and its basic sites can convert complex oxygenated plastics like PET into valuable aromatics.^120^ Furthermore, its high CaO and Fe_2_O_3_ content makes it an excellent in situ catalyst for trapping chlorine during the pyrolysis of mixed plastic waste containing PVC, a critical function for producing cleaner, noncorrosive fuels.^121^ The extensive research on RM thus highlights its potential as a robust, regenerable catalyst for a wide range of feedstocks.

Steelmaking slags like RM are effective catalysts due to their high content of basic oxides (e.g., CaO) and redox-active iron. Electric-arc-furnace slag, for instance, achieved 85.5% deoxygenation of durian-shell pyrolysis oils, the process of which is displayed in ref 122. Beyond direct use, these slags can also be upgraded into higher-value materials. For example, hydrothermal conversion of steel slag into a zeolite creates a microporous, acidic catalyst that can achieve *a* >95% yield in biodiesel production, demonstrating a different pathway for valorization.^123^ Consequently, slags from nonferrous metallurgy also show strong catalytic performance. In the pyrolysis of wastepaper, copper slag dramatically reduced the resulting oil’s viscosity by 59%, an improvement for downstream pumping and processing.^124^ Similarly, sodium-modified aluminum slag proved to be a robust upgrading catalyst for corn cob bio-oil, achieving 64.02% deoxygenation and remaining stable over five regeneration cycles.^125^

Residues from the process industry, particularly the large-volume waste streams from pulp and paper manufacturing, offer another route to effective catalyst synthesis. Calcining kraft-pulp lime mud, for instance, yields a highly basic CaO catalyst (≈3.3 mmol g^–1^). This high basicity is extremely effective for deacidification; in the pyrolysis of Jatropha residues, it eliminated corrosive carboxylic acids and boosted the total hydrocarbon content from 4% to over 37%.^46^ The same catalyst has proven effective for different feedstocks, such as oil-based drill cuttings, where it promoted the cracking of long-chain alkanes to more than double the yield of valuable alkenes.^126^ Conversely, spent or “equilibrium” Fluid Catalytic Cracking (FCC) catalyst from petroleum refineries is a widely available and potent catalyst for pyrolysis. While partially deactivated regarding primary cracking, these materials retain sufficient textural properties and “tempered” acidity to function as robust, sacrificial “guard beds” in dual-catalyst systems. For instance, in lignin pyrolysis, using spent FCC as an in situ catalyst to perform initial cracking resulted in the lowest coke formation on the more sensitive downstream HZSM-5 catalyst, extending its operational life.^127^

Interestingly, the performance of spent FCC can sometimes be more desirable than that of a fresh catalyst. In the fast pyrolysis of corncob, spent FCC produced a higher oil yield with less coke than its fresh counterpart. This is likely because the “tempered” acidity of the partially deactivated spent catalyst is sufficient for cracking target molecules but less prone to catalyzing unwanted polymerization reactions.^128^ Spent FCC is also highly effective for tackling the major challenge of heavy wax formation during plastic pyrolysis. In the cracking of wax from mixed plastics, it increased the liquid oil yield from 4.7% to over 22% with minimal coking, successfully converting a problematic, low-value byproduct into valuable diesel-range hydrocarbons.^93^

#### 2.2.2 Low-Cost Natural Materials

Low-grade clays and naturally occurring zeolites that accumulate as mine tailings or quarry fines possess the acidity, porosity, and thermal robustness required for catalytic upgrading. While naturally occurring, their classification as “waste” often stems from their generation as byproducts of mining operations or their disposal after use as absorbents. Natural clays like montmorillonite, bentonite, and kaolinite provide moderate Lewis and Brønsted acidity, making them versatile catalysts (Figure 1a). They can be utilized directly, with K10 montmorillonite proving highly effective for eliminating wax and achieving a 75% liquid yield during plastic pyrolysis.^129^ They also serve as excellent supports; montmorillonite-supported iron nanoparticles boosted the pyrolysis oil yield from corncob significantly.^130^ Furthermore, their performance can be dramatically enhanced through modification. For example, grafting sulfonic groups onto montmorillonite raised its acidity to enable 88 wt % liquid fuel production from waste cooking oil.^131^ Other clays, such as fibrous sepiolite and bentonite, act as effective, though milder, catalysts. Their gentler activity can be advantageous for preserving liquid yields while still improving oil quality by lowering the acid number and improving storage stability.^132^ Bentonite has shown similar performance for mixed-plastic feeds, increasing liquid yield and narrowing the product’s boiling range.^133^

Discarded tuffs rich in minerals such as clinoptilolite, mordenite, or heulandite offer the key benefit of shape-selective acidity at a much lower cost than synthetic zeolites. After a simple acid wash to expose their Brønsted acid sites, these materials are highly effective; early work showed that protonated clinoptilolite could convert polystyrene almost quantitatively into gasoline-range liquids.^134^ Even in their untreated or mildly treated forms, these natural zeolites are powerful catalysts. They can significantly deoxygenate and dewater–oils from the copyrolysis of plastic and biomass^135^ and can boost liquid oil yields from plastic pyrolysis to over 86 wt % while minimizing gas formation.^136^ Furthermore, the properties of these natural materials are not fixed. Their performance can be dramatically improved via activation, which can more than double their surface area and triple their acidity, unlocking even greater catalytic potential.^137^

#### 2.2.3 Municipal, Post-Consumer, and E-Waste-Derived Catalysts

Municipal solid-waste incinerators, wastewater treatment plants, and postconsumer plastics generate ashes and chars that already contain catalytically active minerals or metals. Repurposing these residues as catalysts simultaneously offsets disposal costs and improves the pyrolysis oil quality. Incinerator bottom ash (IBA) and fly ash (IFA) from MSW plants typically contain a range of metal-based components, usually metal oxides. Municipal incinerated bottom ash’s main components have been found to be CaO, SiO_2_, and Al_2_O_3,_ with some other iron, sodium, and calcium-containing species identified at lower concentrations.^138^ Experimentally, MSW-derived fly ash increased the benzene, toluene, ethylbenzene and xylene (BTEX) yield from plastic pyrolysis by ∼35%; when loaded with nickel, the aromatic oil yield doubled.^139^ These MSW-derived ashes can also serve as catalyst supports (Figure 1b). After a hydrothermal treatment to enhance its properties, Ni-impregnated IBA proved to be a stable and effective tar reforming catalyst, a performance attributed to the synergy between the added nickel and the native iron oxides in the ash.^140,141^ Furthermore, they can be used as reforming agents to enhance H_2_ production from biomass steam reforming, with yields as high as 7.9 mmol H_2_ per gram of biomass reported.^142^

Wastewater biosolids are a source of two distinct catalytic materials: sewage sludge ash (SSA) from incineration and sewage sludge char from pyrolysis. SSA is a mineral-rich material, notable for its high P_2_O_5_ content (15–20 wt %), making it a valuable precursor for phosphate-based catalysts (Table 1). The char, a carbonaceous material often with naturally embedded metals such as iron, is also catalytically active. Used on its own, it can enhance in situ reforming of pyrolysis volatiles, boosting gas yield and H_2_ content.^143^ Its performance is further enhanced when used as a support for active metals; Ni- and Fe/Ni-loaded sludge chars, for instance, are highly effective for tar reforming.^144^ Advanced modifications can lead to the creation of multiple value streams, with trimetallic sludge-derived catalysts able to coproduce two high-value products, hydrogen and multiwalled carbon nanotubes (MWCNTs) simultaneously, demonstrating a highly efficient circular economy model.^145^

The char from plastic pyrolysis represents a powerful “in-process” circular economy opportunity, where a byproduct is used to improve the primary process (Figure 1c). After chemical activation to develop a high surface area (up to 487 m^2^ g^–1^), this char can be used for CO_2_ capture or as a catalyst support.^146^ Crucially, this char is often a composite material, retaining mineral additives like CaO and TiO_2_ from the original plastic formulation, which gives it intrinsic catalytic activity.^147^ As a low-cost reforming catalyst, it has been shown to convert ∼50% of tar model compounds.^148^ In contrast to plastic char, catalysts derived from electronic waste (e-waste) require different synthesis pathways due to their metallic complexity. While plastic char is often utilized directly or after activation, e-waste processing typically involves mechanical separation (ball milling), followed by hydrometallurgical or pyrometallurgical recovery.^149^

The resulting char from nonmetallic e-waste fractions has been shown to consist of a glass-fiber-reinforced carbon matrix naturally enriched with active metals like Cu, Fe, and Ni.^150^ Active metals derived from catalytic converters of cars and PCBs supported on bacteria biomass have been utilized to catalytically upgrade heavy oil, demonstrating comparable activity with typical refinery catalysts.^151,152^ E-waste often contains brominated flame retardants, which contaminate pyrolysis oil. The iron oxides in the e-waste char, whether native or added, promote debromination by capturing bromine to form stable iron bromide salts (FeBr_x_). One study demonstrated 68% debromination while also boosting the yield of valuable single-ring aromatics.^153^ This dual-action performance, enhancing BTEX yields while cleaning the oil, makes these iron-functionalized chars a particularly valuable class of waste-derived catalyst.^139^

#### 2.2.4 Agricultural Residue Silica, Carbon, and Ash Catalysts

Agricultural byproducts contain high loadings of SiO_2_, Al_2_O_3,_ and alkaline-earth or alkali oxides embedded in thermally stable organic or mineral matrices. They are renewable, widely distributed, and often available at zero cost, making them ideal feedstocks for circular-economy catalysts. Ashes rich in silica (SiO_2_) and trace elements of other oxides like alumina (Al_2_O_3_) and ferric oxide (Fe_2_O_3_) represent a significant category of agro-waste catalysts, as shown in Figure 1b.^98^ For example, rice husk ash (RHA) is particularly valued because it provides a source of high-purity, amorphous silica, which is a far more reactive precursor for catalyst synthesis than common crystalline silica like sand.^154^ While RHA can be used as a direct, mild catalyst to enhance hydrocarbon yields,^155^ its primary value is as a sustainable support. In the light of this, a Ni/RHA catalyst successfully deacidified and upgraded pyrolysis oil via esterification, raising its pH from 2.3 to 4.4.^156^ The high quality of RHA-derived silica allows for the synthesis of advanced, highly ordered mesoporous frameworks, such as MCM-41 and KIT-6. These materials enable fine control over the product selectivity through nanoconfinement effects. For instance, in the pyrolysis of rice husk, a Ni/MCM-41 catalyst (derived from RHA) produced a balanced oil, while a Ni/KIT-6 catalyst with larger, more interconnected pores preferentially produced aromatics (67.5%), demonstrating precise tuning of the final product.^155^ This behavior is consistent with RHA’s amorphous, high-purity silica, which is more reactive than crystalline sand for templated mesoporous synthesis. RHA is thus a versatile resource for creating a new generation of low-cost, sustainable catalysts.

Another major class of catalysts is produced from carbon-rich agricultural residues such as biochar, which is a byproduct of biomass thermochemical conversion. It is a plausible and promising catalyst and catalyst support material due to its cost-effectiveness, large specific surface area, and rich surface functional groups that can be tailored through activation processes.^98,157^ Biochar has been utilized as a support for Ni-supported catalyst and applied for HDO of vanillin to *p*-creosol.^158^ Its value lies in its low cost, tunable surface chemistry and physicochemical properties, and its role as a form of carbon sequestration.^98,157^ While biochar has some native catalytic activity, its full potential is unlocked when it is upgraded into AC and functionalized with active metals. Iron-loaded AC (Fe/AC) catalysts, for example, have proven to be highly versatile. In one application, an Fe/AC catalyst was used for in situ reforming of palm kernel shell pyrolysis bio-oil, simultaneously producing a hydrogen-rich gas (75.1 vol % H_2_), and a liquid oil highly enriched in valuable phenol (75.1 area %).^159^ In a different application, an Fe/AC catalyst derived from coconut shell was used for downstream steam reforming of pyrolysis oil, efficiently cleaning the gas by reducing tar to below 100 mg Nm^–3^ while producing a high-quality syngas.^160^ These examples show how a simple pyrolysis coproduct can be engineered into a sophisticated catalyst for diverse upgrading strategies.

Certain agrowastes are notable for their exceptionally high alkali metal content, making them effective catalysts for pyrolysis oil upgrading. Banana peels are a prime example; their ash can contain over 40–50 wt % potassium compounds (e.g., K_2_O and K_2_CO_3_).^53^ This strong basicity profoundly influences pyrolysis reaction pathways, steering them away from acids and toward specific valuable chemicals. It has been shown that alkali metal catalysts can increase phenol content by up to 80% from bamboo pyrolysis or boost cyclopentanone yields to over 50% from cellulose.^52,161^ This activity extends to other sources of alkali metal catalysts, such as coffee husk residue. Its ash contains high levels of K_2_O (46.5 wt %) and CaO (17.7 wt %), is effective for deacidification by reducing phenolics,^162^ and for the catalytic cracking of heavy tars.^163^ While much of the research on these ashes has focused on their successful use in biodiesel production,^164,165^ their powerful effect on pyrolysis product distribution makes them a critical class of upgrading catalysts.

#### 2.2.5 Shell and Bone-Derived Calcium and Phosphate Catalysts

Waste seashells, eggshells, and bones are renewable sources of heterogeneous catalyst rich in alkaline earth material oxide such as CaO, hydroxyapatite, beta tricalcium phosphate, phosphate, and bone char.^113,166^ They provide a versatile, low-cost source for highly basic catalysts, such as CaO. Simple calcination converts the native calcium carbonate (CaCO_3_) into high-purity CaO, a potent catalyst for deacidification (Figure 1d). Its strong basicity drives the conversion of corrosive acids into more stable ketones and alcohols,^45^ effectively improving hydrocarbon content in copyrolysis feeds,^44,167^ and boosting the heating value of upgraded oils.^168^ A critical advantage of using eggshells is the superior durability of the resulting catalyst, which overcomes a major operational hurdle. Studies show that while commercial CaO deactivates rapidly, eggshell-derived CaO maintains significant deoxygenation activity, even after 10 regeneration cycles. This enhanced longevity is attributed to the material’s unique microstructural properties, making it a far more practical option for continuous industrial processes.^45^ However, a review on waste bone-derived hydroxyapatite catalyst and bone char has demonstrated their applications as a catalyst and catalyst support in organic synthesis, biodiesel production, hydrocracking of heavy oil, selective oxidation, and selective hydrogenation.^113^

## 3 Application of Waste-Derived Catalysts in Upgrading PPOs

### 3.1 Waste Plastic Pyrolysis Oils

WPPO is produced by either noncatalytic or catalytic pyrolysis of plastic waste, which is a part of MSW.^31^ MSW mostly consist of polyolefins such as polyethylene (PE), polypropylene (PP), polyethylene terephthalate (PET), polystyrene (PS), and polyvinylchloride (PVC), while automobile shredder residue, and waste electrical and electronic equipment (WEEE) include acrylonitrile butadiene styrene (ABS), polyamide (PA), and poly­(methyl methacrylate) (PMMA). To provide consumers with the right functionality, several additives are added to plastic during the manufacturing process; these additives end up as contaminants in WPPOs. The pyrolysis process leading to WPPO is shown in Figure 2. Pyrolysis, a waste valorization process, produces solid char, synthetic oil (WPPO), and gas products by subjecting plastic waste to temperatures in the range 300–900 °C in an oxygen-free atmosphere. The produced PPO can be viewed as a substitute for a fossil-based feedstock in the production of a broad range of monomers and chemicals. As a result of an uncontrolled feedstock mixture, WPPOs may contain a significant number of contaminants such as heteroatom-containing species (e.g., sulfur, chlorine, nitrogen, etc.). Thus, it can be integrated into refinery olefin production facilities, by providing feedstock for naphtha crackers for ethylene and propylene production.^169^ As a result, waste plastics can be a resource for fuel, new plastics, and other chemicals. The technoeconomic analysis of the production of PPOs from the catalytic pyrolysis of plastic wastes showed prospects for industrialization.^14^ Consequently, associated variation in WPPO composition is a major reason why thermochemical recycling of mixed waste plastics is not yet industrially viable.^15^

**Figure 2:**
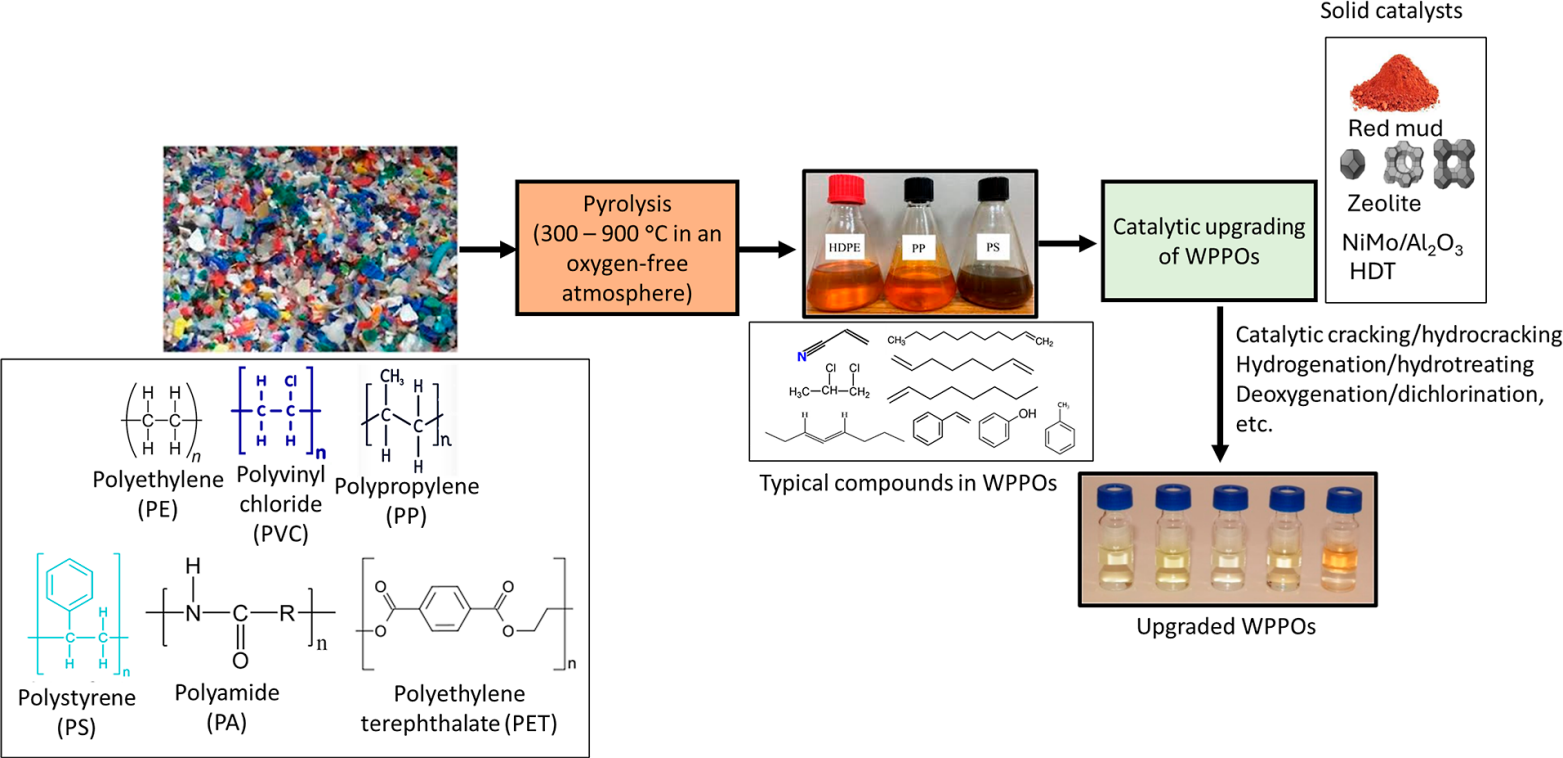
WPPO production from noncatalytic/catalytic pyrolysis of waste plastics.

The process of upgrading WPPO involves removing contaminants and improving their quality so that it can serve as fuel or meet feedstock specifications for chemical and petrochemical processes. The methods of upgrading WPPOs include hydroprocessing technology (i.e., hydrotreating and hydrocracking), catalytic cracking, electrochemical hydrogenation (ECH), and distillation. Recently, a critical review of the recent advancements in electrochemical hydrogenation (ECH) was published.^170^ On the one hand, the variability of the waste plastic feedstocks implies huge variations in underlying chemical compositions and physical properties of the WPPOs. Additionally, some plastics have a lot of inorganic additives, such as carbon black, carbonate, and clay included during the manufacturing process contribute to impurities in the WPPOs.^32^ These additives improve the properties of the plastics but result in impurities, such as sulfur, chlorine, and nitrogen, upon pyrolysis into WPPOs. If not removed, these impurities will become foulants and poisons to catalysts and processing equipment used in downstream processes when PPO is used directly as a feedstock. Hence, impurity and compositional variety are the most significant technical challenges limiting WPPOs from being utilized more widely in downstream petrochemical industries and directly as a fuel.^12^ On the other hand, experimental studies on the properties and suitability of WPPO obtained from high-density polyethylene (HDPE), low-density polyethylene (LDPE), polypropylene (PP), and styrene as diesel fuel have been reported.^171^ The results indicate that WPPO derived from PP exhibited excellent physicochemical properties as a diesel fuel blend compared to the pyrolysis oils of HDPE, LDPE, and styrene. Consequently, the diesel engine performance and emission characteristic of 5, 10, and 15% blend with diesel showed an increased cylinder pressure as well as increased emissions of CO, hydrocarbon (HC) and NO_x_. This can be attributed to the unsaturated (i.e., olefins and aromatics) and long chain saturated hydrocarbons contents. This further demonstrates the need for catalytic upgrading toward targeted fuel-range hydrocarbons and the removal of heteroatoms contaminants. Thus, without proper sorting, mixed plastic waste contaminates the WPPO streams. The challenge is the high costs associated with collection and sorting, large volume of mixed waste plastics, limited sorting technologies, and lack of global standardization.

It is therefore difficult to directly process pyrolysis oil from complex municipal plastic wastes, such as mixtures containing polyethylene (PE), polypropylene (PP), among others, polystyrene (PS), poly­(ethylene terephthalate) (PET), and halogenated polymers like poly­(vinyl chloride) (PVC).^121^ Industrially, while most plastics, including PS, PP, and PE, are considered high-value pyrolysis feedstocks, PVC is identified and removed prior to pyrolysis, as its presence can significantly degrade WPPO quality and complicate downstream upgrading due to chlorine-containing compounds. Hence, catalytic upgrading of WPPOs plays a pivotal role in industrial scale chemical plastics recycling. The composition of the WPPOs can be very complex depending on the nature of the mixed waste plastics and the process conditions.^172^ These heteroatom compounds pose challenges, for example, chlorinated PPO can cause significant corrosion problems during storage, transportation, combustion, etc. and poisons to catalyst during refining.^173–175^ Thus, catalytic upgrading is aimed at lowering contaminants below acceptable limits, cracking large chain hydrocarbons into smaller chains, and concurrently hydrogenating olefins, dienes, and aromatics. PPOs from PE and PP polyolefins are mostly olefins and then paraffins, while those from PS and PET are mostly aromatics, followed by olefins, and then paraffins.^176,177^

Depending on the waste plastic feedstock composition, the WPPO contains a broad range of products, such as olefins, paraffins, naphthene, aromatics, dienes, and heteroatoms such as sulfur (in the form of thiophenes, thiols, etc.), nitrogen (in the form of nitriles, pyridines, etc.), oxygen in the form of ketones, aldehydes, etc. or halogen.^178^ For instance, PVC contains about 20–40% chlorine.^174^ In petrochemical plants, chlorides, a major contaminant in plastic waste streams, can lead to corrosion problems in steam cracking furnaces, causing equipment breakdown. The complexity of organic chlorides in WPPO poses a challenge. Chlorine has been identified in WPPOs in the form of 1,2-dichlorobenzene, 2-chloro-2-methyl pentane, 2-chloroethyl ester, 2-chloro-2-methyl propane, 2,6-dichlorotoluene, chloroethyl benzene, 1,4- benzenedicarboxylic acid, di-2-chloroethyl ester, and 2-chloro-2-phenyl propane^179,180^ Thus, these impurities can increase oil viscosity, poison downstream catalysts, cause plugging of packed bed, or corrode equipment. Furthermore, the WPPOs contained considerable levels of nitrogen, oxygen, chlorine, iron, sodium, and silicon, which exceeded the feedstock specifications for refinery steam crackers.^15^ As a result, the successful integration of a thermochemical recycling process into an industrial petrochemical process will depend on minimizing contaminants present in WPPOs.^181^ Hence, upgrading is aimed at improving the hydrocarbon range and the quality of the PPO by removing impurities in the form of HCl, NH_3_, H_2_O, CO, CO_2_, H_2_S, etc.

WPPO primarily composed of olefins (linear, branched, and diolefinic), paraffins, iso-paraffins, and aromatic hydrocarbons with carbon chain length from C_6_ to C_30_, along with N-, S-, Cl-, and O-containing heteroatomic compounds.^182,183^ Generally, the sulfur content is relatively low, has a high energy value, and has a flash point in the range of diesel. Typical elemental composition and physicochemical properties are shown in Table 2. The properties of WPPO such as viscosity, density, and calorific value vary depending on the plastic mixture, type, and pyrolysis temperature.

**Table 2:** Typical Physicochemical Properties and Element Composition of WPPOs^184–186^

property	value
calorific value (MJ kg^–1^)	28.2–46.1
density at 15 °C (g cm^–3^)	0.74–0.89
viscosity at 40 °C (mPa s)	1.98
ash (wt %)	0.006–0.02
flash point (°C)	26.1–48
pour point (°C)	–67 to −5
C (wt %)	84.63–91.9
H (wt %)	8.31–15.23
N (wt %)	0.06–0.14
S (wt %)	0.01–0.81
O (wt %)	0.64–1.38

### 3.2 The Role of the Catalyst in Upgrading PPO

The catalytic upgrading strategies can be classified as thus: (1) in situ catalytic upgrading (the catalyst is homogeneously mixed the plastic feedstock), (2) ex situ catalytic upgrading of vapors from pyrolysis of plastic wastes (catalyst is packed in another reactor, while the pyrolysis vapors flow through it), and (3) catalytic upgrading of the condensed PPOs (fluidized or packed bed catalyst is used to upgrade the produced WPPOs).^187^ Unlike the ex situ approaches, the in situ catalytic upgrading technique is faced with the challenge of separating spent catalyst from char, and the simultaneous optimization of catalytic upgrading and pyrolysis is difficult. Ex situ catalytic upgrading methods allow the optimization of conditions to ensure optimal catalytic performance. Despite ex situ copyrolysis catalytic upgrading of PPO being energy-efficient, it is difficult to measure and quantify the extent of catalytic upgrading that had occurred. Additionally, ash particles leaving the reactor during waste plastic pyrolysis can become entrained in the gas/vapor stream, which can build-up, deactivate, and plug the catalyst bed downstream in the case of ex situ catalytic upgrading.^188^ Thus, this review focuses on the catalytic upgrading of PPOs. The primary role of catalysts in upgrading of WPPO includes improving oil quality via cracking, reforming reactions, facilitating the incorporation of hydrogen, and removing undesired contaminants.

The catalytic upgrading process of WPPO with the aim of producing fuel or naphtha includes hydrocracking (HCK) and hydrotreating (HDT) reactions. Some studies focus on catalytic upgrading of WPPO into aliphatic monomers, primarily ethylene, propylene, and butene derivatives.^169,189^ One option for the catalytic upgrading of WPPOs is the production of light olefins (i.e., C_2_–C_4_: ethylene, propylene, and butene). These monomers are building blocks for plastic production, indicating that this approach is a recycling pathway to commercialization. Thus, in addition to removing impurities, catalytic upgrading of WPPO can be designed to target fuel range hydrocarbons, naphtha (i.e., straight-chain C_5_–C_9_ hydrocarbons) feedstock, or light olefins (C_2_–C_4_) production. Thus, WPPOs are potential feedstocks for the production of drop-in fuels, chemicals, and petrochemical feedstocks (e.g., ethylene, propylene, and butene; C_2_–C_4_), closing the plastic recycling loop, as shown in Figure 3.

**Figure 3:**
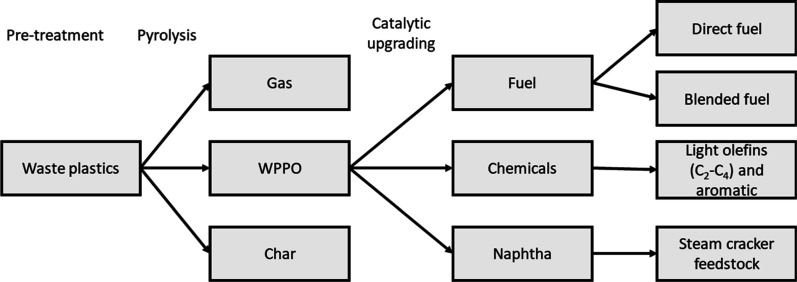
Target products of the catalytic upgrading of WPPOs.

The complexity of WPPO’s composition places additional demands on catalyst design and development, which must consider the characteristics of the molecules and contaminants that are present in WPPOs. As a result, key characteristics of effective heterogeneous catalysts derived from industrial waste for upgrading WPPO oil are shape selectivity, strong and adjustable surface acidity, well-defined pore architectures, and high surface area.^190^ Other important characteristics include mono- or bimetallic doping such as Ni, Mo, Co, and others to promote catalytic activity, reduce coke formation, and improve heteroatom removal (e.g., deoxygenation, denitrogenation, dehalogenation, etc.). Studies have shown that the catalyst’s pore structure and acid sites play significant roles in the performance of catalytic upgrading of hydrocarbons of different fuel ranges.^191^ It has been reported that zeolite-based catalysts with meso- and macro-porous network structures are beneficial for the diffusion of long-chain polymeric hydrocarbons commonly found in WPPOs.^192^ Interestingly, catalysts with zeolitic compositions, morphologies, and structures can be prepared from industrial waste such as RM, BFS, CFA, biomass waste, and clay materials (Figure 1b), due to their silica and or alumina-rich components.^111,193^ This approach offers a sustainable pathway to zeolite catalyst preparation from industrial waste.

Zeolitic catalyst compositions can be synthesized from most of the industrial solid wastes (e.g., RM, CFA, blast furnace slags, and natural clays) because they contain SiO_2_ and Al_2_O_3_.^111,193^ For instance, BFS has been used as a precursor to synthesize zeolite MCM-41 through leaching 5 M HCl for 3 h at 100 °C followed by dissolution in 5 M NaOH solution and hydrothermal treatment at temperature 100 °C for 24 h.^194^ The synthesized MCM-41 was utilized as a catalyst support in upgrading of tire pyrolytic oil (TPO) into fuel, with 35 wt % of gasoline and 33% diesel fractions. Therefore, understanding the effect of physicochemical properties of zeolite on upgrading of WPPO would provide insight into pretreatment and synthesis process customization for waste-derived catalysts from various industrial wastes. Figure 4 shows the effect of catalyst properties on catalytic upgrading of WPPO. In comparison to thermal cracking, while the n-paraffins content increase and its iso-paraffins drop in the upgraded oil, the zeolite’s Si/Al ratio increases through dealumination with oxalic acid (Figure 4a), and the total acidity (Brønsted + Lewis acid sites) decreases from 0.341 mmol g^–1^ (Si/Al = 25) to 0.103 mmol g^–1^ (Si/Al = 130).^195^ This indicates that the extent of catalytic cracking increases as the acidity of the catalyst increases. High acidity favors gas yields, coke formation, and olefin selectivity. It was found that the application of a catalyst greatly reduced chlorine levels in liquid and gas products compared with thermal cracking (Figure 4b), with a further reduction noted after doping with iron oxide.^196^ This result indicates that the ratio of Si/Al in the preparation of a catalyst from industrial waste would play a crucial role in the cracking and decontamination of WPPO. Therefore, in the design of catalysts with zeolitic composition, such as industrial waste (Figure 1b), a tradeoff is required between acid site-induced cracking activity and metal dopant decontamination.

**Figure 4:**
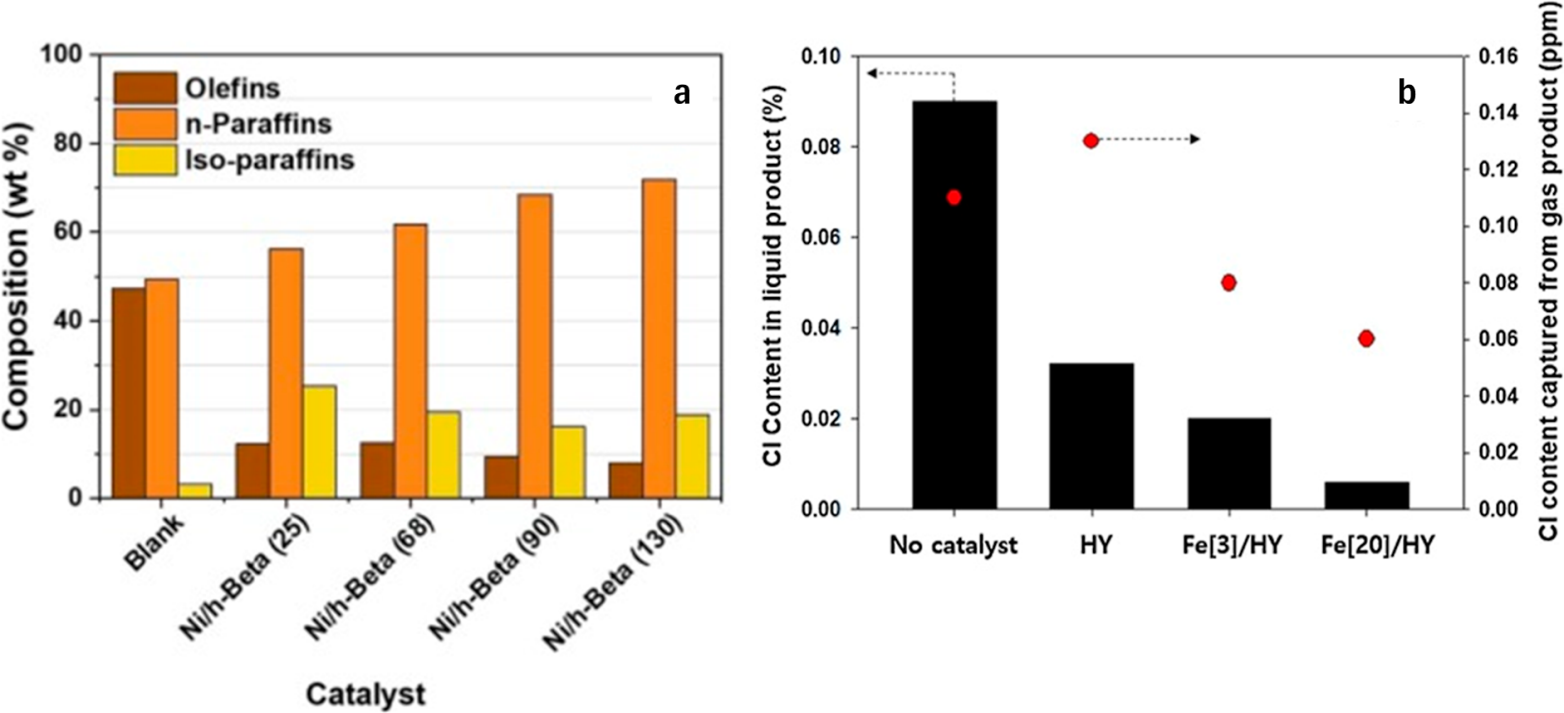
Effect of catalyst properties (a) zeolite-based catalyst Si/Al ratio (25 to 130) and acidity on the liquid composition with hydrocarbons less than C_12_ fraction in the conversion of model LDPE over Ni/h-Beta catalysts (Reproduced from ref 195 Available under a CC-BY-NC-ND 4.0 license. Copyright Briones et al.) and (b) effect of iron oxide doped HY zeolite on chlorine removal (Reproduced from ref 196 Available under a CC-BY-NC-ND 4.0 license. Copyright Hwang et al.).

The effect of pore structure and shape selectivity of zeolite-based catalysts based on the conversion of 1-octene as a model compound representation of polyolefin PPO at 500 °C and 1 atm was studied.^197^ It was found that zeolites with a small pore size (pore diameter ≤0.61 nm) facilitated catalytic cracking of octene, yielding up to 98% of small olefins (54% C_5_), while large pore size (pore diameter ≥0.61 nm) zeolite results in aromatization to BTX (benzene, toluene, and xylene) and paraffin products. This observation elucidates the role of catalyst pore sizes on product selectivity and feedstock conversion. Thus, waste-derived catalysts must possess both Brønsted and Lewis acid sites, which are crucial for cracking the long polymeric chains found in WPPOs into shorter hydrocarbons. Hence, the physicochemical properties of waste-derived catalysts, such as particle size, pore size distribution, specific surface area, number and strength of acid sites, can be linked to their performance, activity, and lifespan. It is important to note that the primary obstacle to commercialization is the greater cost of products from pyrolysis of waste plastic compared to petroleum products.^31,198^ Thus, the cost of upgrading PPOs can be significantly reduced by developing and utilizing robust catalysts derived from waste materials. The zeolitic component compositions of most industrial waste materials make them potential candidates for synthesizing catalysts for catalytic upgrading of WPPOs. Thus, scale-up and industrial applications of catalysts synthesized from industrial waste and natural clays would significantly improve process economics.

### 3.3 Waste-Derived Catalysts for PPO Upgrading

By indirectly improving energy security, plastic-derived fuels are a viable energy source that can lessen the reliance on fossil fuels and offer a pathway to recycle huge amounts of waste plastics. However, direct application of PPO is faced with challenges of poor fuel quality due to contaminants, high viscosity, and low calorific value.^199^ Most studies reported in the literature on catalytic upgrading of WPPO are majorly based on zeolite (e.g., ZMS-5, HY zeolite, etc.) and hydrotreating (NiMo/Al_2_O_3_, CoMo/Al_2_O_3_, W/Al_2_O_3_, etc.) catalysts.^197,200^ The cleavage of C–C bond occurs over solid acid catalysts and bifunctional catalysts (acid sites and metallic sites), which perform catalytic cracking, C–C bond rearrangement, isomerization, hydrocracking, and hydrogenolysis.^201^ In addition to the cost factor impeding industrial scale-up, they possess strong acid sites, especially zeolites that produce high yields of light fractions, gaseous products, and coke, and are easily prone to deactivation. However, waste-derived catalysts offer tunable acidity via pretreatment and a sustainable alternative to conventional catalysts. Additionally, waste-derived materials can be used as catalysts or support due to their mixed metal oxides nature, such as Fe_2_O_3_, Al_2_O_3_, and SiO_2_.^111,202^ This allows the impregnation of active metals such as Ni, Co, and W, etc. to design bifunctional catalysts. Furthermore, this catalyst development strategy reduces waste disposal costs, promotes the circular economy, and converts industrial waste and clay into high-value functional materials. This has positive effects on the scalability and the environment. A study has shown that calcined spent FCC catalysts (HY zeolite and MCM-41) produced diesel range hydrocarbons comparable to commercial zeolites (HY and MCM-41) but exhibited low coke formation in the catalytic upgrading of WPPO (HDPE, LDPE, and PP at a ratio of 1:1:1 at 600 °C) at 300 °C.^93^ Depending on the catalyst type, acid site strength, pore size distribution, and catalytic upgrading conditions of temperature, residence, and feedstock composition, typical cracking reaction schemes are shown in Figure 5.

**Figure 5:**
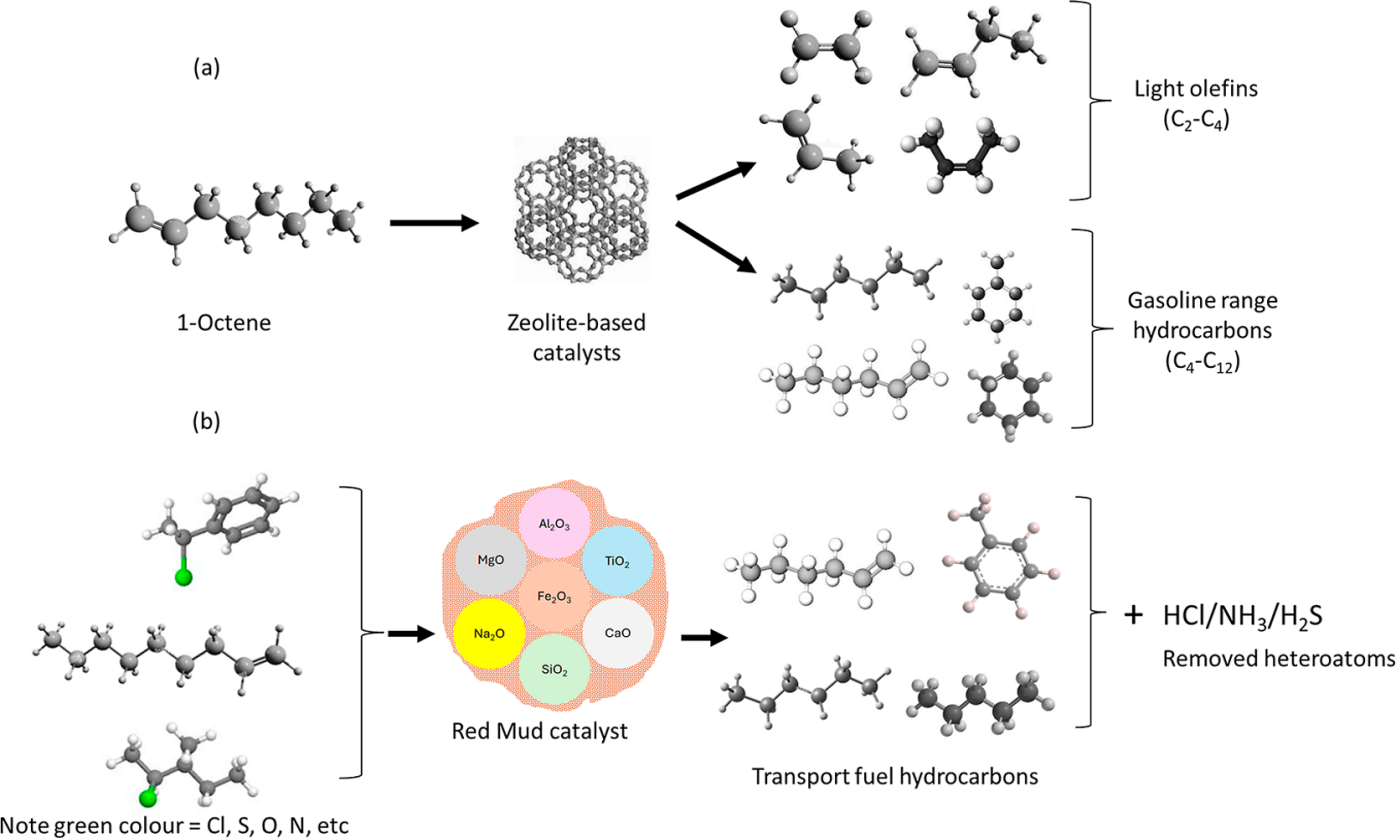
Catalytic upgrading over (a) spent zeolite-based catalysts and (b) RM-derived catalysts.

Annually, about one million tons of waste FCC catalysts are generated from oil refining processes, which have diminished catalytic activity for cracking hydrocarbons and are generally landfilled.^203^ This waste FCC catalyst contains valuable metals and moderate acid sites strength. Refinery waste FCC catalysts are mostly applied in the catalytic pyrolysis of waste plastic to improve oil quality and its fuel fractions and reduce heteroatoms/contaminants.^204–207^ In upgrading WPPO, spent FCC catalyst has demonstrated potential in improving the yield of diesel-range hydrocarbons and suppressing aromatic production at 200–400 °C for 90 min.^93^ To further improve the performance, the waste FCC catalyst can be activated through sequential acid treatment (e.g., nitric, oxalic, or sulfuric acid) and pore structure and acidity enhancement by the ammonium hexafluorosilicate method to improve physicochemical and catalytic properties. An activated waste FCC catalyst demonstrated improved selectivity toward light olefins from 5.79–19.49% and the yield of light fuels from 21.67–29.76% in catalytic pyrolysis of municipal mixed plastic waste.^208^ Therefore, the activation of waste FCC catalysts can be harnessed for upgrading WPPO.

#### 3.3.1 Red mud, Clay, and Slag-Derived Catalysts

There are numerous studies on the in situ catalytic pyrolysis of different waste plastics over waste-derived catalysts into fuel oils; PET, polycarbonate (PC) and polyurethane (PU) over RM,^120^ LDPE, PP, PET, and PVC over slags from steel industries as catalysts,^209^ HDPE, LDPE, PP, PE and PS over fly ash,^210,211^ and PE, PP, and PS over biochar catalysts.^102,212^ Compared to the noncatalytic methods, industry-derived slags and RM catalysts significantly reduced the halogen content in WPPO and improved the fuel-range hydrocarbons.^209,213,214^ These research results support the use of industrial waste-derived catalysts to catalytically upcycle difficult waste plastics, making them a sustainable resource for catalysts from the perspective of the circular economy. However, this study focuses on the catalytic upgrading of WPPOs using low-cost, natural materials, waste-derived heterogeneous catalysts.

The chlorine content of steam cracker feedstock must be kept within the limit of 10 ppm.^215^ RM has exhibited excellent catalyst activity for the dehydrochlorination and cracking of chlorinated WPPOs.^121,216^ The upgraded WPPO exhibits characteristics like gasoline and diesel-like products with chlorine content less than 0.1 wt %.^121^ It can be deduced that the acidic characteristics of the zeolitic nature of the alumina-silicate Al_2_O_3_ and SiO_2_ components are responsible for the catalytic activity of RM. RM is a rich mixed oxide of Fe, Si, Al, Na, and Ca.

The catalytic activity of RM can be linked to compositional variance, which is determined by its bauxite ore origin. Transportation, acid, alkali, and water washing, as well as energy for drying and calcination, contribute to costs associated with RM synthesis and purification into catalysts. However, a magnetite-rich catalyst can be derived from RM through hydrogen reduction at temperatures greater than 300 °C. RM exhibited comparable catalytic activity with iron oxide (TR99701) and iron oxide–carbon composite (TR99300) catalysts and an excellent stability for 5 h time-on-stream operation (Figure 6a). Consequently, RM showed excellent dehydrochlorination activity of MWP-derived oil at 350 °C (Figure 6b). In the catalytic upgrading of mixed waste plastics (PE, PP, PS, PVC, PET, and ABS) oil with 600 ppm organic chlorine compounds in a fixed bed reactor at 300 °C, RM and iron oxide catalysts exhibited stable activity for 6 h time-on-stream, while the activity of zinc oxide (ZnO) and MgO catalysts drastically declined within 3 h.^216^ The liberated HCl gas poisons and rapidly deactivates ZnO and MgO catalysts compared to RM.

**Figure 6:**
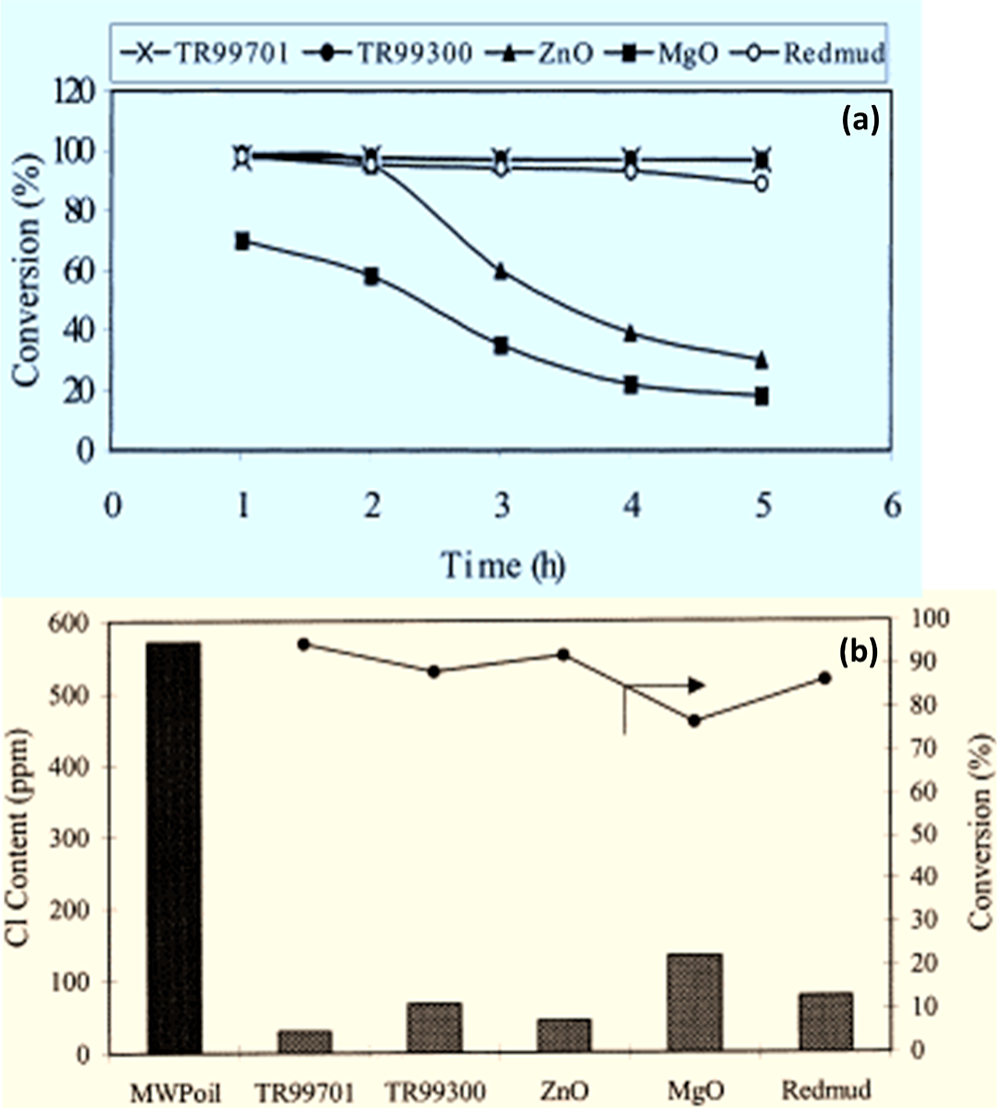
Catalytic performance of RM against conventional catalysts: (a) time-on-stream activity 300 °C and (b) steady–state activity in the dehydrochlorination of MWP-derived oil at 350 °C. Reprinted (adapted) with permission from ref 216. Copyright © 2001 Elsevier Science Ltd.

The iron oxide and RM catalysts showed superior performance and stability in the removal of organic chlorine compounds in the mixed waste plastic oil. The dechlorination activity of calcined, hydrogen-reduced, and HCl-treated RM, and Fe/SiO_2_ catalysts was studied using chlorobenzene, a model representative of typical chlorinated compounds in WPPO at a reaction temperature of 350 °C. Based on the results, RM produced superior conversion performance compared to the Fe/SiO_2_ catalyst for time-on-stream operations beyond 50 min, and similar chlorine adsorption capacity.^215^ The major elements of RM such as iron adsorbed chlorine and get converted to iron chloride (FeCl_*x*_). This indicates that other elements of RM such as alumina contribute to its catalytic activity and not only the major iron component. Consequently, RM was more effectively regenerated than Fe/SiO_2_, indicating its stability and reusability. The performance of RM makes it a plausible low-cost candidate for further investigation as a catalyst for the catalytic upgrading of WPPOs.

A comparative analysis revealed that ZSM-5 zeolite possesses a higher acidity and larger specific surface area (412.0 m^2^ g^–1^) compared to RM with moderate acidity and specific surface area of 27.49 m^2^ g^–1^.^217^ Hence, zeolite exhibited stronger catalytic activity than RM. However, RM offers cost-effective and sustainable catalytic upgrading compared to zeolite-based catalysts. The acid sites of waste-derived catalysts such as RM and kaolin clay are responsible for cracking reactions as well as oligomerization, aromatization, and coking of catalysts. However, compared to conventional zeolite-based catalysts, waste-derived catalysts like kaolin clay and RM have lower surface acidity because of their lower Brønsted acidity, which could be beneficial in minimizing secondary reactions leading to higher coke formation.^218^ The mild acidity of the RM material can be attributed to the presence of SiO_2_, Al_2_O_3_, and TiO_2_ component. Likewise, clay material montmorillonite possesses a weaker acidity compared to zeolites.^209^ In the case of catalytic upgrading of waste mineral oil, the produced oil based on catalytic cracking over RM at temperatures 400 and 425 °C contained higher contents of low boiling hydrocarbons than that achieved via thermal and catalytic treatment with hydrotreating catalysts NiMo/Al_2_O_3_ and SiO_2_-Al_2_O_3_.^219^ This reveals the hydrogenation and cracking activity of RM due to the mixed metal oxides, particularly reduced active Fe metal. It has been observed that the activity of RM as a catalyst is not significantly impacted by the reduction temperature.^215^ This implies that the sensitivity of RM dechlorination activity to the reduction of the iron oxide content is negligible. Furthermore, RM, montmorillonite clay, desulfurization slag, and Linz Donawitz slag were used to upgrade WPPO from a mixture of LDPE, PP, PS, PET, and PVC.^209^ The oil catalyzed by slag contained the most aliphatic hydrocarbons, whereas those catalyzed by montmorillonite clay and RM contained a somewhat larger fraction of aromatics. This is because montmorillonite clay and RM contain SiO_2_, Al_2_O_3_, and TiO_2_, which induce similar compositions and properties to zeolite (i.e., Brønsted acidity). The results showed that waste-derived catalysts (RM, montmorillonite clay, and slag) reduced halogen concentration to the same level as hydrotreatment catalysts Pd/Al_2_O_3_ and NiO/Al_2_O_3_.^209^

MCM-41 material has been hydrothermally synthesized from BFS and used as a catalyst support in the hydrocracking of WPPO into fuel using a fixed-bed reactor at 375–475 °C, 40–80 bar, and 1.2 h^–1^ liquid hourly space velocity.^194^ The Ni-W supported on MCM-41-derived from BFS by hydrothermal synthesis exhibited high hydrocracking activity and 97.7% hydrodesulfurization of TPO into fuel at 475 °C.^194^ The upgraded oil upon fractionation contained 35 wt % gasoline and 33% diesel. 5 wt % Ni loading on kaolin (5 wt % Ni/kaolin) catalyst utilized nickel-induced deoxygenation activity and kaolin’s moderate acidity to enhance cracking of WPPO at 280 °C.^220^ When a 5 wt % Ni/kaolin catalyst was used, a gasoline fraction of 28.2% and a middle fraction of 40.0% were obtained, which is slightly lower than the gasoline fraction of 30.0% achieved with a 5 wt % Ni/ZSM-5 catalyst and a middle fraction of 38.8% under identical operating conditions. Although the upgraded oil with a 5 wt % Ni/ZSM-5 catalyst produces a little greater gasoline fraction, it requires the more costly ZSM-5. The application of Ni/RM catalyst for catalytic upgrading of bio-oil improved hydrogenation and promoted moderate cracking and reduce coke formation compared to the commercial catalyst.^221^ The Ni/RM catalyst exhibited excellent catalytic activity for upgrading bio-oil, yielding 68.6% liquid and 4.2% coke relative to 41.8 and 7.3% obtained using the Ni/SiO_2_-Al_2_O_3_ catalyst. The impact of WPPO composition due to heterogeneity of waste plastic, upgrading temperature, and the nature of waste-derived catalysts on the yields of paraffins, olefins, aromatics, and deoxygenation is shown in Table 3.

**Table 3:** Waste-Derived Catalysts Performance Key Indicators (Deoxygenation Efficiency and Aromatic Hydrocarbon Yield)

waste-derived catalyst	paraffins (%)	olefins (%)	aromatics (%)	deoxygenation (%)	reference
RM at 400 °C	98.70	0.29	1.01		^222^
kaolin at 520 °C	43.3	30.8	4.5	21.4	^220^
5 wt % Ni/Kaolin at 280 °C	36.4	40.4	5.2	22	^220^
Ni-W/BFS at 375–475 °C			40–60	20–50	^194^
80% RM, 10% C-based, 10% kaolin at 350 °C	48	33.5	18.5		^223^
90% RM and 10% kaolin at 350 °C	45	35	20		^223^

The catalytic mechanism of multimetal oxides in RM, slag, or clay material is primarily driven by the synergistic effect of their major components, such as iron oxide (Fe_2_O_3_), alumina (Al_2_O_3_), and silica (SiO_2_) for RM, which function as active sites, supports, and promoters for various reactions. This synergy of the multimetal oxide composition can induce a range of reaction mechanisms: redox, oxygen vacancies, acid/basic sites, and support and textural effects.^224^ For instance, the iron oxide component based on the lattice oxygen on its surface can activate redox reaction, which facilitate the transfer and activation of oxygen species due to its ability to cycle between different oxidation states (Fe^3+^/Fe^2+^).^225^ The synergy among the different metal oxides can also create surface oxygen vacancies, which can act as active sites for adsorbing and activating reactant molecules. Additionally, the Al_2_O_3_ and SiO_2_ content in RM, slags, and clays provides acid sites, which facilitates C-C bond cleavages, cyclization, isomerization, and other reactions.^226^ The residual alkali/alkaline earth metals (CaO, MgO, etc.) provide base sites and facilitate the removal of S and Cl. Consequently, the presence of SiO_2_ and Al_2_O_3_ provides support and textural effects for these waste-derived catalyst materials. These components can primarily act as stable support materials for impregnation of suitable active metals to improve selectivity, contributing to a high specific surface area and porous structure. Thus, the catalytic activity of waste-derived multimetal oxide catalysts can be further optimized through modifications like acid/heat/hydrothermal treatments or metal doping to enhance specific active sites and tailor their composition and textural properties.

#### 3.3.2 Ash/Biochar-Derived Catalyst

MSW ash produced by incineration was calcined at 550 °C for 2 h, pelletized to a size 0.2–2 mm, and then used to support 30 wt % Ni for reforming of WPPO.^139^ The WPPO was obtained from the pyrolysis of a mixture of 70% PP, 24% HDPE, and 6% LDPE at 500 °C.^139^ The result showed that the produced oil contained gasoline-like (C_6_–C_12_), diesel-like (C_13_–C_19_), and heavier hydrocarbons (C_20_–C_35_) fractions. However, the calcination of MSW ash-derived catalyst slightly increased the gasoline-like and diesel-like fractions in the produced fuel oil. This indicates that the catalytic activities of waste-derived catalysts (e.g., ash) can be enhanced through pretreatment. However, MSW ash (raw and calcined) catalysts produced greater yields of the BTEX (i.e., benzene, toluene, ethylbenzene, and xylene) products relative to the thermal control experiment. The effect of doping the calcined MSW ash with Ni from 15 to 30% resulted in an increase in the yield of heavier hydrocarbons (C_20_–C_35_) and lower BTEX content due to an increase in the pore size, lower acidity, and hydrogen incorporation functionality of Ni-doped ash. This implies that a waste-derived catalyst with a smaller pore size may favor the yield of lighter hydrocarbons, while larger pores favor heavier hydrocarbons.^197^ On the other hand, the application of biochar derived from agricultural and biomass residue via pyrolysis for upgrading WPPO produced higher yields of gasoline and diesel carbon-range hydrocarbons C_9_ and C_12_.^227^ Biochar-based catalysts offer excellent pore structure, large surface area, rich surface functional groups, and good coking resistance.^29^ Consequently, the physicochemical properties and catalytic activity of biochar-based catalysts can be enhanced through physical activation (steam or carbon dioxide), chemical activation (acid or alkali), and functionalization.^29,158^ Table 4 summarizes the outcome from the application of low-cost, natural materials and waste-derived catalysts for the catalytic upgrading of WPPO. Notably, the product distribution is a function of the WPPO feedstock composition, the nature of the catalyst used, reactor type, and operating conditions.

**Table 4:** Selected Waste Catalysts for Upgrading of WPPOs

feedstock	catalytic upgrading conditions	outcomes	ref
WPPO from a mixture PE, PP, PS, PET, and PVC obtained at 500 °C, 1 atm, and 120 min	red mud (RM), batch reactor, 325 °C, 1 atm, and 30 min	(1) gasoline and diesel range hydrocarbon liquid produced	^121^
		(2) Upgraded oil chlorine content <0.1 wt %	
		(3) RM-derived catalyst demonstrated effectiveness for organic chloride removal	
WPPO obtained from the pyrolysis of PE, PP, PS, PVC, PET, and ABS at 410 °C	Fe_3_O_4_, γ-Fe_2_O_3_, MgO, ZnO, and RM, fixed bed, 300 and 350 °C	(1) iron oxide and RM catalysts exhibit a similar pattern of activity	^216^
		(2) the catalytic activity of ZnO and MgO rapidly decreased due to HCl	
WPPO obtained from HDPE, LDPE, and PP at a ratio of 1:1:1 at 600 °C	calcined spent FCC catalysts (HY zeolite and MCM-41), 300 °C, 90 min	(1) high yield of oil and low coke formation	^93^
		(2) produced diesel range hydrocarbons	
WPPO from waste electric and electronic equipment (WEEE) at 300–450 °C	Si–Al ash pellets, fixed bed, and 450 °C	(1) oil contains broad range of aromatic hydrocarbons	^228^
		(2) about 40% coke on the spent catalyst	
WPPO from (70% PP, 24% HDPE, and 6% LDPE) at 500 °C	Ni supported on MSW incineration ashes, fixed bed, and 500 °C	(1) selective activity toward benzene, toluene, ethylbenzene, and xylenes production	^139^
		(2) the produced oils contain 38–45% (gasoline range), 28–32% (diesel range), and 23–33% (heavy fraction) hydrocarbons	
waste plastic heavy distillate fraction	kaolin clay catalyst [SiO_2_ (54.8%), Al_2_O_3_ (41.12%), MgO (0.48%), K_2_O (0.26%), and P_2_O_5_ (0.09%) and 3.22% trace elements], batch reactor, 350–400 °C, and catalyst loading 5–15 wt %	(1) high yield of paraffins and olefins with diesel-range (C_6_–C_23_) carbon	^218^
		(2) kaolin improved the fuel properties of the upgraded liquid compared to thermal	

## 4 Application of Waste-Derivative Catalysts in Upgrading Biomass Pyrolysis Bio-Oils

Pyrolysis and hydrothermal liquefaction (HTL) are utilized to produce bio-oil from biomass. The complexity of biomass feedstock, just like mixed waste plastics, results in variation in the composition of bio-oil produced. These thermochemical processes convert a variety of biomass, including wood, agricultural residue, organic materials, etc., in the temperature range of 200–600 °C.^229^ Under the reaction conditions, the large and complex components in biomass such as cellulose, hemicellulose, and lignin break down to smaller components in the form of oil (bio-oil), gas, and solid char. While WPPO contains a wide range of heteroatoms (e.g., N, S, O, Cl, etc.) and other contaminants, bio-oil obtained via pyrolysis or HTL of biomass is a viscous liquid, rich in oxygenated compounds (e.g., phenols, aldehydes, alcohols, ketones, carboxylic acids, furans, water, etc.). The high oxygen content (35–40 wt %) compounds induced corrosiveness (i.e., acidity), high viscosity, poor miscibility with conventional fossil fuels, and low calorific value, limiting direct applications of bio-oils as substitute transportation fuels.^230^ It therefore requires further upgrade to enhance its stability and energy content, making it a promising alternative to conventional fossil fuels. Consequently, the carbon numbers of the majority of oxygenate organic compounds present in bio-oil are predominantly C_2_ to C_9_, which is shorter than the range of carbon chain found in WPPO (WPPO). Hence, catalytic upgrading involves removing oxygen to improve the physicochemical properties of the bio-oil. The carbon neutrality of biomass-to-biofuel alternative to fossil fuels, would play a crucial role in climate change mitigation by decarbonizing the transport sector. Upgrading bio-oil into biofuel offers greenhouse gas (GHG) emission reductions of 65–85% compared to fossil fuels.^231^

Bio-oil from lignocellulosic biomass pyrolysis is a multicomponent mixture of organic compounds and water, with major components being oxygenated organic compounds such as phenolics, carboxylic acids, esters, alcohols, ketones, furans, aldehydes, and carbohydrates and heavier lignin-derived polymers. The physicochemical properties and element composition of bio-oils are shown in Table 5. Its compositions are governed by feedstock, pyrolysis conditions, and catalysts.^232^ Bio-oils are dark, viscous, and acidic with a low heating value, high oxygen content, and carbon number in the range of C_2_ to C_18_.^233^ Compared to WPPO, bio-oils are unstable and highly acidic and have higher oxygen content and lower carbon and hydrogen content. As a result of these properties, bio-oils require upgrading to improve the fuel properties.

**Table 5:** Typical Physicochemical Properties and Element Composition of Bio-Oils^234^

property	value
calorific value (MJ kg^–1^)	13–18
density at 15 °C (g cm^–3^)	1.10–1.30
viscosity at 40 °C (mPa s)	15–35
water content (wt %)	15–30
acidity (pH)	2–3.7
flash point (°C)	40–110
ash (wt %)	0.004–0.3
pour point (°C)	–36 to −9
C (wt %)	50–60
H (wt %)	5.5–7
O (wt %)	35–40
S (wt %)	<0.05
N (wt %)	0–0.2

### 4.1 Role of Catalysts in Improving Bio-Oil Quality through Hydrotreating

Unlike WPPO, where catalysts are expected to withstand a range of contaminants as well as olefin and aromatic contents, biomass pyrolysis bio-oils require catalysts with high deoxygenation activities and tolerance for in situ produced water during upgrading. To facilitate the removal of oxygen and other heteroatoms, catalysts would play a crucial role in improving bio-oil quality through hydrotreating. However, the complexity of bio-oil results in a broad spectrum of reactions during catalytic upgrading, which includes cracking, decarbonylation, decarboxylation, hydrocracking, hydrodeoxygenation (HDO), hydrogenation, and polymerization.^106^ This leads to the removal of oxygen in the forms of water (H_2_O), carbon dioxide (CO_2_), and carbon monoxide (CO). Therefore, low-cost catalysts with robust activity and stability to facilitate these target reactions are appropriate for the catalytic upgrading of bio-oil. However, to catalyze deoxygenation, the catalysts for bio-oil upgrading can be categorized into zeolite-based and metal-based (e.g., metal sulfide, metal oxides, metal phosphides, etc.) catalysts.

Catalysts, designed out of various noble and transition metals supported on metals and/or carbon-based support, have been used to promote HDO, hydrodenitrogenation (HDN), and HDS. These reactions, usually performed under H_2_ pressure (>20 bar) and temperature (200–400 °C), convert reactive oxygenated compounds into more stable hydrocarbons, while reducing acidity and increasing energy density. Many catalysts were reported to have excellent activity in improving the bio-oil quality; at the same time, limitations, including low stability, limited selectivity, and inconsistent performance, remain an existing concern. Moreover, noble metal-based catalysts could be expensive, making the overall industrial scale-up and economy of the process less feasible. In addition to being costly, these conventional catalysts suffer rapid deactivation due to char formation and deposition, leading to reduced efficiency and lifetime. Therefore, waste-derived catalysts can promote the technoeconomic viability of the multistage upgrading processes. The wide range of mixed metal oxides of waste-derived catalysts could promote stability in the aqueous, acidic, and reactive environment of bio-oil during upgrading. As a result, waste-derived catalysts offer sustainable and economic solutions to address the high-cost associated with the synthetic catalysts.^229,235–237^ Moreover, these catalysts can be synthesized and tuned to have catalytic activity, selectivity, and stability comparable to those of similar expensive synthetic catalysts.

### 4.2 Waste Catalysts for Bio-Oil Hydrotreating

#### 4.2.1 RM-Derived Catalysts

A variety of waste-derived catalysts such as metal- and carbon-based catalysts have been reported for bio-oil upgrading through hydrotreatment (Table 6). One of the waste-derived catalysts is RM, a byproduct of alumina production, which offers a rich matrix of Fe_2_O_3_ and other metal oxides that can be modified through active metal impregnation to enhance its catalytic activity. RM has been utilized to prepare various catalysts for the bio-oil hydrotreating process.^238–246^ An extensive study on catalysts with Ni-doping on RM and their applications in the hydrotreatment process has been performed so far. The incorporation of Ni improves the dispersion of active sites and enhances hydrogenation and deoxygenation efficiency, making Ni-doped RM a cost-effective and sustainable alternative to conventional catalysts.

**Table 6:** Various Waste-Derived Catalysts for Bio-Oil Upgrading Through the Hydrotreatment Process

catalyst	feedstock	temp. (°C)	important active sites in the catalyst	yield/selectivity	ref
Red mud catalysts					
Ni/RM	palmitic acid	260–300 °C	multiple valance Ni (Ni^0^, Ni^2+^) and iron (Fe^0^, Fe^2+^, Fe^3+^) on RM	92.54% fatty alcohol and 100% alkane content	^239^
Ni-Mo/RM	palmitic acid	250 °C	Ni^0^, Mo^0,^ and Fe^0^ species alongside SiO_2,_ Al_2_O_3,_ and Fe_2_O_3_	100% conversion efficiency with enhanced deoxygenated components	^240^
Ni/acid treated RM	palmitic acid	300 °C	Ni species and Fe_2_O_3_ (Fe^3+^) species	81% cetane selectivity	^241^
Ni-RM-500	palmitic acid	250 °C	Ni^0^ and Fe^0^ species	100% palmitic acid conversion with hexadecanol as the dominant product	^242^
Ni (nitrate/carbonate)/RM	palmitic acid	320 °C	Ni (nitrate/carbonate) species	81% selectivity to cetane (*n*-hexadecane) using nickel nitrate on RM. 85% selectivity to pentadecane using nickel carbonate on RM	^244^
Co/ARM	kraft lignin	350 °C	synergistic effects between the Co metal and the acid sites	>90% yield of catechols	^245^
Mo–Ce/RM	alkali lignin	270 °C	synergistic effect of Mo with Ce with metal oxides of RM	91.6% conversion, 81.8 wt % bio-oil yield, and 65.4 area % selectivity of vanillin	^246^
Fly Ash catalysts					
coal fly ash	waste chicken fat	444.4 °C	NA	76.2 wt % bioil yield, including 24.0 wt % gasoline-range hydrocarbons	^247^
Slag catalyst					
Ni/coal gasification slag	benzyl phenyl ether	180 °C	Ni^0^, Ni^2+^, and Ni^3+^ sites	>99% selectivity to monocyclic products	^248^
steel slag	*Pteris vittata* L.	300 °C	CaO, MgO, MnO, Al_2_O_3_, and Fe_2_O_3_	25.35% bio-oil yield with a HHV of 30.27 MJ kg^–1^	^249^
Carbon catalysts					
Ni/coconut lumber sawdust-derived hierarchical porous carbon	*Callophyllum inophyllum* oil	550 °C	Ni metal and active sites of carbon support	74 wt % liquid product yield, with 65 wt % hydrocarbons	^250^
Ni-Mo/phosphorus-rich activated carbons	lignin	350 °C	Ni_12_P_5_ and MoNiP	oil yields of ∼70 wt % and monomer production of >10 wt %	^251^
Ni and Zn/rice husk synthesized Y zeolite	oak wood biocrude	330 °C	Zn^0^, Fe^0^, and Ni^0^ sites	oil yield of ∼80% with an enhanced H/C ratio of 1.08	^252^
Sulfided CoMo/coconut shell-derived activated carbon	4-methylacetophenone	280 °C	sulfides of CoMo	100% conversion with almost 100% selectivity to ethyl methylbenzene	^253^
Ni-Mo/*Jatropha curcas* leaves-derived carbon	gas oil and Jatropha oil	370 °C	NA	sulfur content reduction to 52 ppm was observed	^254^
Ni-Mo/pea pod (*Pisum sativum* L)-derived carbon	gas oil and Jatropha oil	370 °C	NA	sulfur content decreased to 20 ppm	^255^
Pd/biomorphic carbon	sunflower oil	≤300 °C	Pd and PdO species	4% residual oxygen content and HHV of 47.53 MJ kg^–1^	^256^
CoNO_3_/DF	carinata oil	400 °C	CoNO_3_	89.3% liquid yield, ∼44 MJ Kg^–1^ HHV, and a decreased viscosity of ∼6.1 cP	^257^
Pt/CSACN_3_-700	*Calophyllum inophyllum* oil	550 °C	highly dispersed Pt species along with a high surface area (271.5 m^2^ g^–1^)	84.77 wt % liquid yield with 91 wt % hydrocarbon selectivity	^258^
W/Mo_2_C	canola oil	250 °C	W_2_C, WC, and Mo_2_C phases	>95% conversion and 76% hydrocarbon yields	^259^
CoMo/DF	eucalyptus pyrolysis bio-oil and poultry fat	400 °C	Co_3_O_4_ and Co(OH)_2_ and MoO_3_ species	74.6 wt % liquid yield, 42.1 HHV (MJ kg^–1^), and a reduced O content of 2.5 wt %	^260^
MoNi/Biochar	used cooking oil (UCO)	310 °C	well-dispersed Mo and Ni species, high porosity, and enhanced acidic sites	complete conversion of UCO and 32 wt % green diesel yield	^261^

Waste animal fats and vegetable oils offer a sustainable feedstock for renewable fuel and chemical synthesis. Palmitic acid is a fatty acid that can be converted to higher fatty alcohols and alkanes using the HDO process. In a recent study, catalysts were developed from RM after modifying its surface with Ni as an active metal for the HDO of palmitic acid.^239^ Experiments performed on the effect of Ni loading on RM and reaction temperature suggested 7 wt % Ni loading as optimum to achieve the highest efficiency. Fatty alcohol and alkane content of 92.54 and 100% was achieved, which was found to be better than commercial catalysts, including Ni-SiO_2_ and Ni/γ-Al_2_O_3_. The activity of the Ni-RM catalyst was attributed to the coexistence of multiple valence Ni (Ni^0^ and Ni^2+^) and iron (Fe^0^, Fe^2+^, and Fe^3+^) on its surface, promoting the hydrodeoxygenation, decarbonylation, and decarboxylation reactions. Deoxygenation through decarboxylation and decarbonylation often leads to loss of carbon atoms and reduced fuel yield compared to direct deoxygenation. Developing a catalyst that can predominantly show high selectivity toward direct deoxygenation is highly desired. Therefore, tailoring the catalyst’s oxophilicity and surface structure suitable for direct deoxygenation is highly important. Ni-red mud catalysts though possessed suitable activity in the HDO of palmitic acid, the reaction pathway was dominated by decarbonylation and decarboxylation.^240^ To improve the direct deoxygenation selectivity through C–O bond cleavage of the Ni-RM catalyst, it was further modified with MoO_X_ to obtain a microstructure Ni-Mo/RM catalyst. Defective MoOx and FeOx species formed near metallic Ni in the catalyst promoted C–O bond cleavage, increasing the hexadecane formation through direct deoxygenation. Various experiments performed with varying MO loading revealed a low (7 wt %) loading on RM as more suitable to carry our palmitic acid HDO with 100% conversion at 250 °C and higher selectivity to direct deoxygenation. Components of RM such as SiO_2_ and Al_2_O_3_ helped in improving surface area and metal dispersion, while Fe_2_O_3_, due to its reductivity, enhanced oxophilic character, and oxygen vacancy, promoted the direct deoxygenation.

The presence of alkali metals such as Na_2_O and CaO in RM has been found useful in many reactions.^229^ However, these metal oxides, when not active for a particular reaction, could limit the catalytic efficiency of a reaction by blocking the pore channel to active sites. To study these effects on HDO of palmitic acid, Duan et al.^241^ designed a Ni-doped acid-treating RM catalyst. Acid treatment using acetic acid was done to remove the soluble alkali (Na_2_O and CaO) components from the RM. It led to increased surface area and pore structure in RM, promoting better Ni dispersion. Moreover, an increased Fe_2_O_3_ content contributed to higher oxygen vacancies and Fe^3+^ species, suitable for the redox characteristics and stability of the catalyst. The Ni/RM-HAC catalyst obtained by acetic acid treatment and Ni doping could achieve almost 81% cetane selectivity, rather than around 65% selectivity for the catalyst without acid treatment. Enhanced C-C cleavage over metallic Ni sites produced C_15_ products, while the oxygen vacancy in the catalyst facilitated HDO followed by dehydration and hydrogenation to produce C_16_ alkanes (Figure 7).

**Figure 7:**
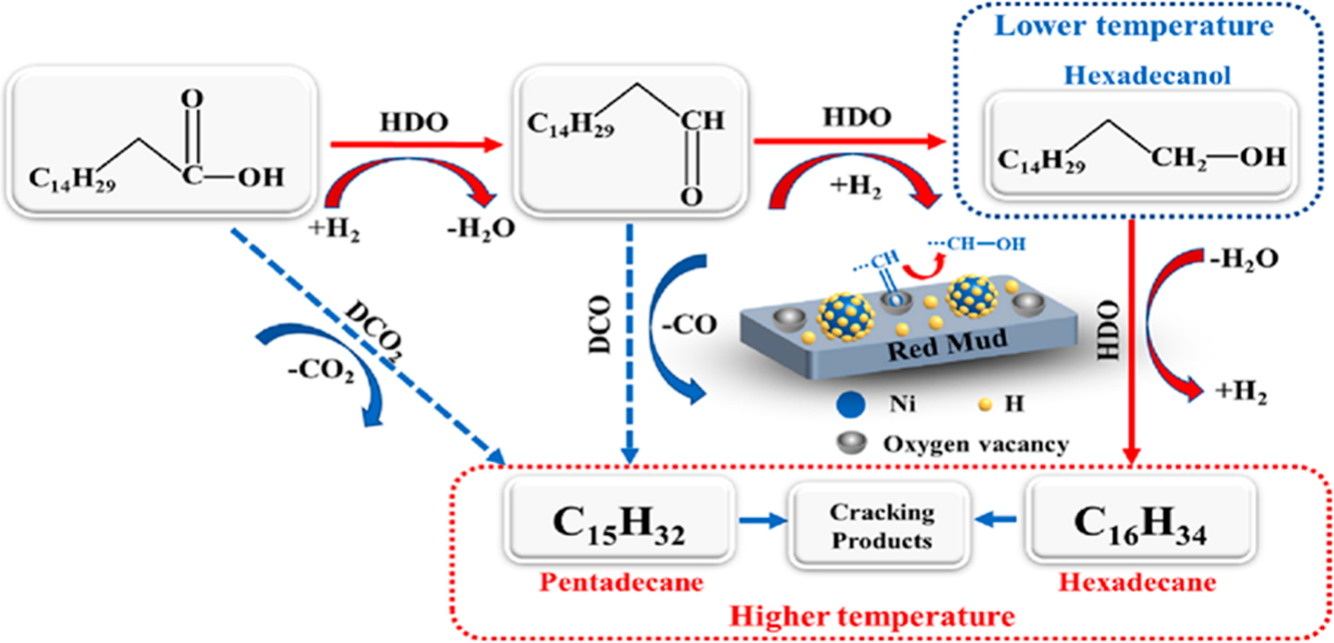
Hydrodeoxygenation mechanism of palmitate acid over the Ni/RM-HAC catalyst, reproduced with permission from Duan et al.^241^ Copyright © 2023 Elsevier Science Ltd.

The effect of calcination temperature on the activity of Ni modified RM catalyst for the HDO of palmitic acid was investigated by Wang et al.^242^ Low calcination temperature in the range of 400–600 °C induced high surface area and better pore volume, leading to better Ni dispersion. Increased calcination temperature in the range of 700–900 °C resulted in metal oxide sintering (Figure 8), decreased surface area and pore volume, contributing to low reduction capacity and CO_2_ desorption. The highest surface area of 77 m^2^ g^–1^ was observed at 500 °C calcination temperature with an enhanced surface Ni and Fe species, smaller particle sizes, and stronger basicity. The Ni-RM-500 catalyst could achieve 100% palmitic acid conversion with hexadecanol as the dominant product at 250 °C, while at 300 °C, the product selectivity shifted entirely to alkane.

**Figure 8:**
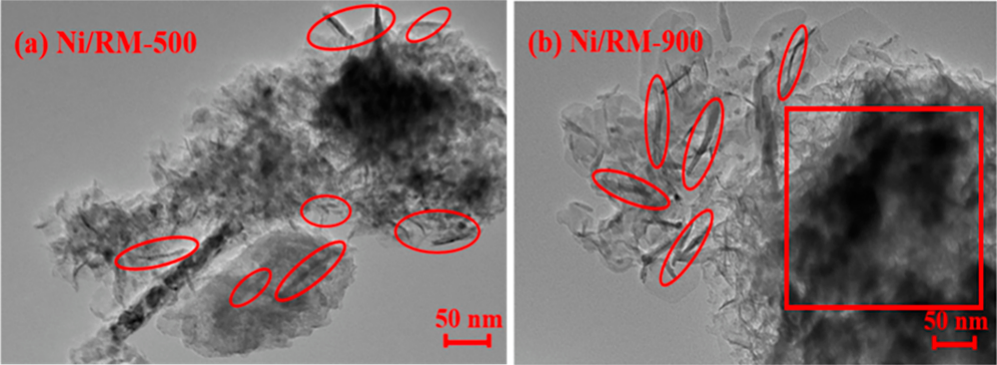
TEM images of (a) Ni-RM-500 and (b) Ni-RM-900 reproduced with permission from Wang et al.^242^ Copyright © 2022 Elsevier Science Ltd.

As discussed in the above paragraphs, Ni on RM catalysts exhibited suitable activity in the HDO of fatty acids. However, the nature of the nickel precursor could influence the catalytic activity, product distribution, and stability to a significant extent. To study these effects, Duan et al.^244^ evaluated six different Ni precursors such as nickel acetylacetonate, nickel acetate, nickel chloride, nickel carbonate, nickel nitrate, and nickel oxide, supported on RM for the palmitic acid HDO. Nickel nitrate on RM favored the HDO pathway (C-O bond cleavage), achieving up to 81% selectivity to cetane (*n*-hexadecane). In contrast, nickel carbonate on RM promoted the decarbonylation/decarboxylation (C-C bond cleavage) reactions, hence, resulting in 85% selectivity to pentadecane. Although nickel chloride on RM also favored the direct deoxygenation reaction, it showed less stability than nickel nitrite on RM. Efficient and selective depolymerization of lignin into valuable monomers remains a significant challenge. Acid (HCl)-treated RM (ARM) doped with transition metals such as Ni, Cu, and Co as catalysts was explored for the hydrogenolysis and hydrogenation of kraft lignin.^245^ Among these transition metals, Co-doped ARM exhibited the highest activity with >90% yield of catechol and alkyl-catechols at 350 °C reaction temperature. A synergistic interaction between Co metal and acidic sites in ARM support, enabling hydrogenolysis, demethylation, and hydrolysis, could be the reason for the higher catalytic performance of Co-ARM catalyst. Effective cleavage of β-O-4′, β–β, and α-O-4′ linkages by Co-ARM led to a reduction in molecular weight from 1721 to 582 g mol^–1^ and produced monomers such as catechol, 4-methylcatechol, 3-methylcatechol, and 4-ethylcatechol (Figure 9).

**Figure 9:**
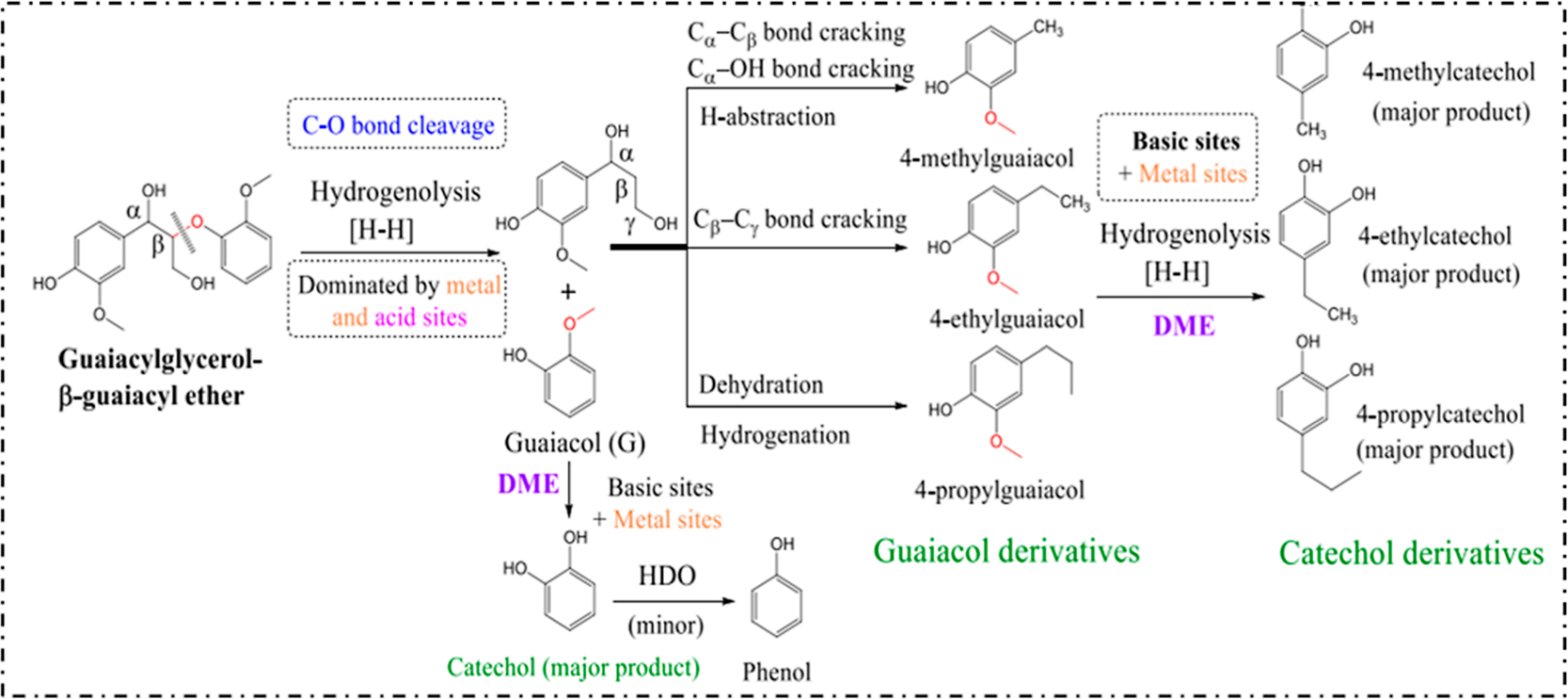
Possible reaction routes for the formation of different monomers during KL depolymerization over a Co/ARM catalyst, adapted from ref 245. Copyright © 2025 Elsevier Science Ltd.

The mechanism over various multi-oxide catalysts derived from industrial solid waste like slags and RM is based on the analysis of reaction pathway using techniques such as GC–MS, NMR, and acidity/basicity analyses. A combination of Ni and Mo metals supported on RM catalysts could enhance hydrodeoxygenation of bio-oil by promoting C–O bond cleavage and exhibit high hydrogen consumption due to Fe_2_O_3_ reduction to FeO and Fe^0^ species. The presence of Al_2_O_3_ in RM was found to generate strong acid sites, which promoted a direct deoxygenation reaction. Alkane, particularly hexadecane, was found to dominate in the Ni-Mo/RM catalyst having Fe_2_O_3_ than Ni-Mo supported on Al_2_O_3_ and SiO_2_ only. RM showed an advantage due to its combined surface properties (SiO_2_/Al_2_O_3_) and the oxophilicity of reduced Fe species that promoted effective hydrodeoxygenation.^240^ In the case of Ni-N/RM catalyst, Ni in Ni^0^ and Ni^2+^ was found to promote medium-strong acidic sites density and oxygen vacancy, promoting the HDO by producing protons, helping in the activation and hydrogenation of palmitic acid.^244^ This pathway enhanced oxygen removal and converted palmitic acid to alcohols, which were further dehydrogenated and deoxygenated to form oxygen-free alkanes. Among other mechanism studies over the Ni-RM catalyst, iron species in the oxophilic FeOx (Fe^0^ and Fe^2+^) form was found to activate the C–O bond by chemisorbing oxygen at vacancy sites.^242^ It followed proton donation from adjacent hydroxyl groups and subsequent desorption. Ni species in the catalyst could dissociate H_2_ to produce hydrides, which promoted oxygen removal from the C–O bond via dehydration. This suggests that activated RM is a potential catalyst support for HDO and hydrotreating of bio-oil into fuel-range hydrocarbons.

#### 4.2.2 Fly Ash-Derived Catalysts

Despite the abundance and solid chemicals (e.g., alumina, silica, and iron oxides) richness of fly ash, very limited research has been conducted on its use as a catalyst or catalyst support for hydrotreatment of bio-oil.^247^ Its catalytic potential remains largely underexplored compared to other industrial wastes, such as RM. Liquid biofuel production through thermal catalytic cracking of waste chicken fat performed under H_2_ pressure (100 kPa) using CFA as a heterogeneous catalyst was explored by Suchamalawong et al.^247^ A maximum bio-oil yield of 76.2 wt %, including 24.0 wt % gasoline-range hydrocarbons, was achieved under optimized conditions. The CFA catalyst promoted both deoxygenation and cracking, improving bio-oil quality through (i) reduction in O content and acid value (3.9-fold decrease), (ii) increase in the heating value to 42.92 MJ kg^–1^, and (iii) increase in the H/C ratio (indicating higher saturation and energy density). Initial reaction pathway on the CFA catalyst involved the hydrolysis of triglycerides to heavy oxygenated intermediates, which, after carboxylation, carbonylation, and hydrogenation, produced heavy linear hydrocarbons. These hydrocarbons were subsequently cracked into paraffins and olefins. There are highly limited studies on using fly ash as a hydrotreatment catalyst.

#### 4.2.3 Slag-Derived Catalysts

Despite their promising chemical composition and availability, studies on slags as catalysts or catalyst support are very limited in the context of hydrotreatment of bio-oil upgrading. Slags, including BFS, steel slag, and copper slag, contain a range of metal oxides (e.g., CaO, Fe_2_O_3_, SiO_2_, Al_2_O_3_, and MgO) that could serve as active components or robust supports for catalytic systems. However, only a few studies have explored their potential after suitable modifications, such as metal doping, to create active sites for improved hydrotreatment catalytic activity.^248,249^ Catalytic hydrogenolysis of lignin using Ni-supported coal gasification slag (Ni/CGS) was reported by Xie et al.^248^ Catalyst prepared with 10 wt % Ni on coal gasification slag was active in promoting selective cleavage of the C aliphatic–O bond, producing a complete conversion of benzyl phenyl ether (a lignin model compound). The results could be obtained under mild reaction conditions of 180 °C and 2 h. A phenolic-rich product with >99% selectivity to monocyclic products demonstrated the high catalytic efficiency of the catalyst in C-O bond cleavage in lignin. Hydrothermal liquefaction (HTL) offers an efficient route for converting hyperaccumulator plants into energy-rich products such as bio-oil and hydrochar. However, the aqueous phase of HTL frequently contains elevated levels of heavy metals, particularly when using metallophyte biomass such as *Pteris vittata*L. (PVL), which poses significant environmental and regulatory challenges. Steel slag was utilized as a catalyst to enhance metallophyte biomass *P. vittata* L. bio-oil quality through hydrothermal liquefaction.^249^ Steel slag as a catalyst was observed to enhance the deoxygenation reaction during HTL and produced a maximum bio-oil yield of 25.35% with a higher heating value (HHV) of 30.27 MJ kg^–1^.

#### 4.2.4 Waste-Derived Carbon-Based Catalysts

Carbonaceous materials, particularly those derived from waste streams including biomass residues, plastics, or agro-industrial byproducts, have been increasingly recognized for their potential as catalysts or catalyst supports for bio-oil upgrading via hydrotreatment. These carbonaceous materials have advantages including their low cost, abundance in nature, and environmental friendliness. In addition, the existing preparation methods through physical and chemical activation can produce carbonaceous materials with high surface area, tuneable porosity, and surface functionalities, making them excellent support for noble and transition active metals doping (Figure 10).

**Figure 10:**
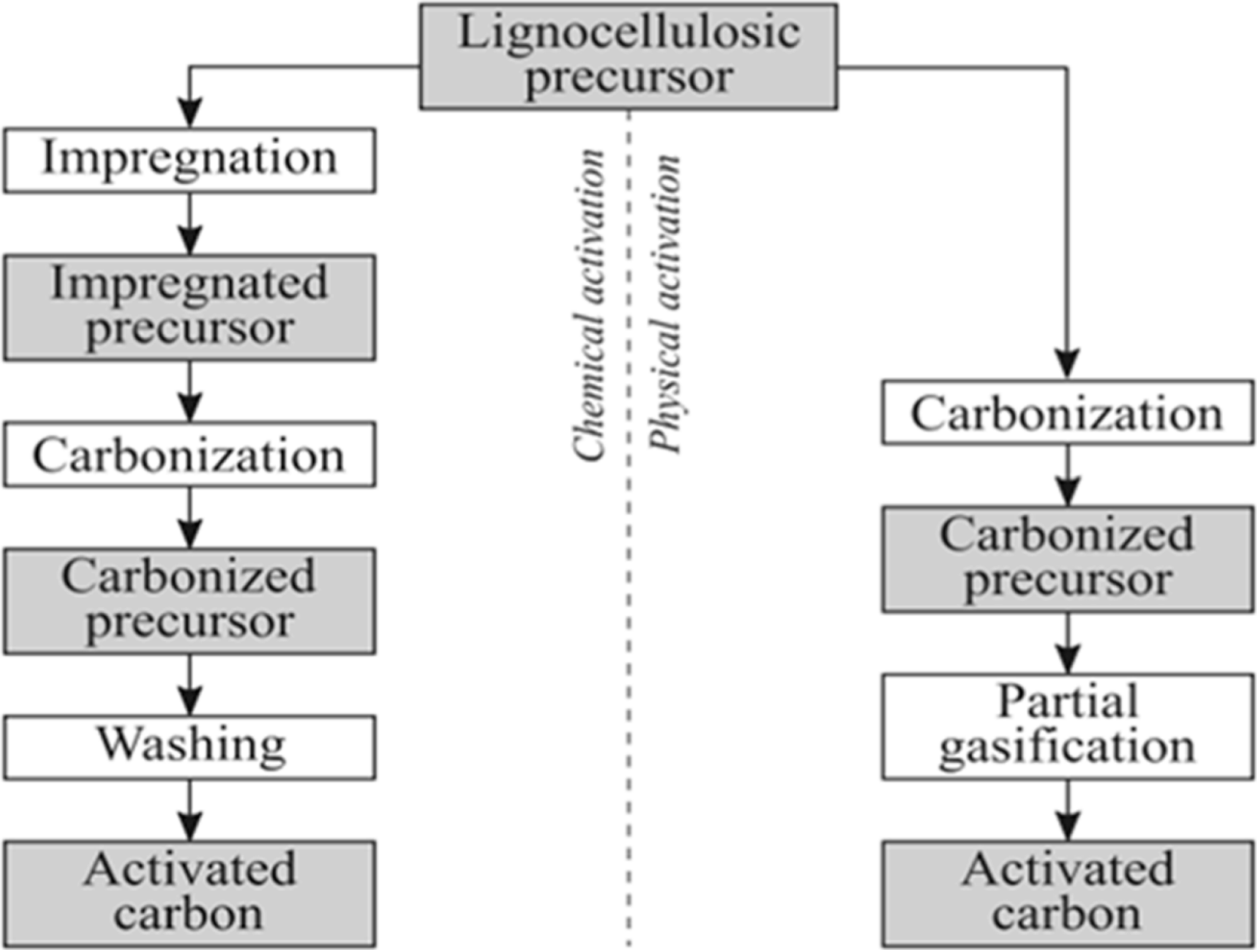
Routes for the preparation of AC reproduced with permission.^262^ Copyright © 2021 American Chemical Society.

Moreover, their tunable surface chemistry can promote hydrodeoxygenation, which is crucial for improving the stability and energy content of bio-oil.^250–266^ Thus, the use of waste-derived carbonaceous material as catalysts or catalyst support could improve the efficiency and sustainability of bio-oil upgrading and simultaneously address waste management and resource utilization challenges.

In a recent study by Trisunaryanti et al.^250^ hierarchical porous carbon (HPC) synthesized from lignocellulosic waste, coconut lumber sawdust was used as a support material for Ni incorporation. HPC prepared using K_2_CO_3_ as a green activating agent and at 700 °C, demonstrated appreciable porosity and adsorptive capacity (surface area, 96.61 m^2^ g^–1^ and average pore diameter, 2.73 nm). When impregnated with 2.76 wt % Ni, the resulting Ni/HPC3-700 catalyst exhibited a significant increase in the surface area of 400.6 m^2^ g^–1^ and enhanced acidity (1.076 mmol g^–1^). These properties of the catalyst contributed highly toward its catalytic performance for the hydrotreatment of *Callophyllum inophyllum* oil. The catalyst achieved over 74 wt % liquid product yield, with 65 wt % hydrocarbons, predominantly in the kerosene and diesel range at 550 °C. Importantly, Ni/HPC3-700 required five times less Ni than conventional supports to achieve comparable conversion, highlighting the potential of the catalyst for biofuel production from nonedible oils. A similar effort in lignin valorization highlighted the synthesis of phosphorus-rich activated carbons utilizing lignin via a straightforward chemical activation with H_3_PO_4_.^251^ Carbon supports with high mesoporosity (Vmes >1.0 cm^3^ g^–1^) and surface areas exceeding 1000 m^2^ g^–1^ were obtained using the proposed technique. When doped with Ni and Mo, the NiMo-carbon catalyst served as high active catalyst for the solvent-free reductive fractionation of organosolv lignin. The formation of active species in the form Ni_12_P_5_ and MoNiP resulted in high activity of the catalyst with oil yields (∼70 wt %) and monomer production (>10 wt %) comparable to commercial Ru/C catalysts. The high activity of the NiMo-carbon catalyst for sulfur-rich technical lignin hydrotreatment, along with the effective deoxygenation reflected in its atomic O/C ratio, comparable to those reported for other NiMo-based catalysts, underscored the robustness and versatility of this waste-derived catalytic system (Figure 11).

**Figure 11:**
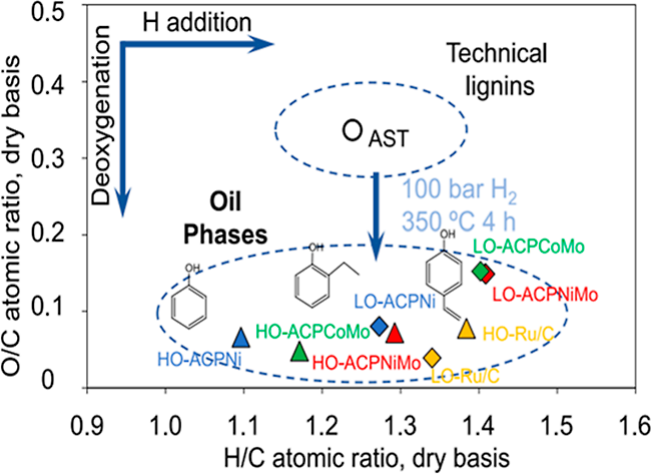
Van Krevelen plot for product oil obtained by the catalytic reductive fractionation of AST lignin using different catalysts. Reaction conditions: 15 g of AST lignin, 0.75 g of catalyst, initial H_2_ pressure 100 bar, and 4 h at 350 °C. Light Oil, lighter oily fraction, and HO (Heavy Oil), heaviest oily fraction, reproduced with permission.^251^ Copyright © 2025 Elsevier Science Ltd.

Biocrude upgrading under in situ hydrogen generation using waste-derived catalysts offers significant economic and environmental advantages over the conventional hydrotreatment process. In a study by Hamidi et al.^252^ the use of Ni and Zn supported on Y zeolite synthesized from rice husk was used for the hydrothermal upgrading of oak wood biocrude. The catalyst exhibited significantly higher acidity (24–28% increase) and enhanced mesoporosity compared to commercial counterparts, leading to improved catalytic performance. An upgraded oil yield of ∼80% with an enhanced H/C ratio from 1.02 to 1.08 indicated the effective deoxygenation and hydrodeoxygenation potential of the catalyst. Zn doping in the catalyst-mediated hydrogen generation and helped to reduce coke formation on the catalyst. Improved bio-oil quality through the upgrading of furan derivatives to cyclopentanones and the hydrogenation of lignin-derived compounds was obtained in the presence of the catalyst.

The influence of surface chemistry modifications of coconut shell-derived activated carbon (CSAC) on the performance of CoMo-based sulfided catalysts for the hydrodeoxygenation of bio-oil model compounds was evaluated.^253^ The AC support was treated with HNO_3_ at different temperatures to introduce varying levels of surface oxygen species. These modified supports, when doped with CoMo, demonstrated excellent performance with complete conversion of 4-methylacetophenone at 280 °C. Strong interaction between oxygen-containing surface groups and the metal precursors resulted in variations in the nature of the metal sulfide phases formed during activation steps. These sulfide phases modulate catalytic selectivity when used for different bio-oil model compounds. Supports with higher oxygen content enhanced decarboxylation selectivity during the conversion of ethyl decanoate and shifted the phenolic product distribution from phenol to catechol in the conversion of guaiacol. Metal precursor types and methods of their incorporation on biochar supports could influence the hydrotreating potential of biochar-derived catalysts. Douglas Fir (DF) waste biomass-derived biochar was employed as support for Ni and Co incorporation via wetness impregnation using nitrates and aqueous dispersion using hydroxides precursors.^257^ Metal dispersions within biochar pores were found for nitrate-derived metals, while hydroxide-derived metals resided mainly on the external surface. The metal dispersion in the catalysts influenced the carinata oil cracking-hydrotreatment (CC-HYD) reaction process. For instance, predominant methanation and aromatization over nitrate-based catalysts indicated that these reactions were favored within the porous structure, whereas metals on the external surface promoted decarboxylation, hydrogenation, and cracking of triglycerides. The upgraded bio-oil exhibited superior fuel quality with 89.3% liquid yield, ∼44 MJ Kg^–1^ HHV, and a decreased viscosity of ∼6.1 cP in the presence of CoNO_3_/DF catalyst. CSACs prepared through alkali (NaOH/KOH) activation were reported by Trisunaryanti et al.^258^ NaOH-activated CSACs (CSACN3-700) and KOH-activated CSACs (CSACK2–700) were subsequently impregnated with Pt and Ni, respectively, to evaluate their hydrotreatment performance with *Calophyllum inophyllum* oil (ClO). A high Pt dispersion along with a high surface area (271.5 m^2^ g^–1^) in Pt/CSACN3-700 led to superior catalytic performance, yielding an 84.77 wt % liquid product with 91 wt % hydrocarbon selectivity. Meanwhile, Ni/CSACK2-700 achieved slightly lower yields (78.83 wt %, 86.2 wt % hydrocarbon selectivity) owing to its lower surface area of 138.6 m^2^ g^–1^.

A systematic exploration of the impact of carburization temperature and metal loading on the deoxygenation potential of catalysts was performed by Tran et al.^259^ Catalysts consisting of bimetallic molybdenum–tungsten carbides supported on spruce-, fir-, and pine-derived biochar were developed for the hydrotreatment of canola oil. A detail characterization of catalysts revealed a consistent Mo_2_C formation in the temperature range (550–700 °C). For the tungsten phase, a transition from metallic W and W_2_C at ≤600 °C to WC at 700 °C was observed. The WC phase existed at higher temperature and exhibited an increased density of hydrogen-activating sites and favored HDO (Figure 12). Nonetheless, lower temperature phases promoted decarboxylation/decarbonylation (DCO) reactions through greater CO adsorption. A significant improvement in bio-oil upgrading efficiency was observed when W was incorporated into Mo_2_C, with >95% conversion and 76% hydrocarbon yields, indicating a synergistic effect in the bimetallic catalyst.

**Figure 12:**
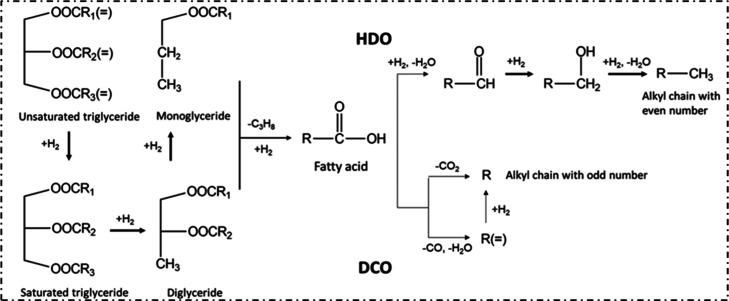
Proposed mechanism of HDO of canola oil over metal carbides, reproduced with permission.^259^ Copyright © 1999 Royal Society of Chemistry.

### 4.3 Catalyst Stability and Deactivation Mechanism

The stability of catalysts and their resistance to deactivation are important factors that can directly influence operational efficiency and economic viability. In addition to performance (i.e., activity, selectivity, stability, and lifespan), regenerability, reusability, and scalability of the catalyst all affect its cost, which is crucial to the overall economics of a catalytic upgrading process on an industrial scale. Catalysts are subject to a harsh reaction environment (high H_2_ pressure and temperature) and a broad range of contaminants and oxygenate compounds during the catalytic upgrading process, which can lead to deactivation through mechanisms such as coke deposition, sintering, and poisoning. For instance, crude WPPOs have a fouling impact on catalysts and accelerate the formation of coke because they contain more and different contaminants than fossil feedstocks.^181^ In catalytic cracking of WPPO into light olefins (C_2_-C_4_) using ZSM-5 catalyst, coke yield was found to be 1.8 wt % compared to 0.2 wt % from petroleum naphtha feedstock.^267^ For bio-oil, char-forming intermediates such as polyaromatics can undergo polymerization and condensation into coke on the acidic/basic sites of the catalysts. The polymerization of reactive compounds in bio-oil can also lead to pore blockage and active site coverage. Furthermore, the acidic nature of bio-oil can cause catalysts to deactivate due to structural phase changes. Therefore, the stability of various natural materials and waste-derived catalysts and their deactivation mechanism during the hydrotreatment of bio-oil are explored. Various RM-derived catalysts used so far for the hydrotreating of bio-oil showed appreciable stability. For instance, under a continuous-flow fixed-bed reactor, the Ni-Mo/RM showed good stability with 100% selectivity to alkanes up to 100 h,^240^ after which a decline in activity was observed (Figure 13a). A decreased conversion and hexadecane selectivity and enhanced olefin and fatty alcohols in the product indicated a reduced hydrogenation efficiency of the catalyst over 100 h. The reason for the reduced activity could be attributed to the reduced synergy between Mo and Ni species due to carbon deposition on the Mo species. Similarly, the Ni-RM/HAC catalyst showed appreciable stability with 99% conversion and C_15–19_ product selectivity up to 240 h (Figure 13b).^241^ A decline in the conversion and products mostly composed of alcohols and pentadecanes indicated the decreased hydrogenation efficiency of the catalyst over 240 h. Oxygen adsorption on catalytic sites, causing Ni site oxidation and formation of Ni-Fe_2_O_4_ species over reaction time, was an important reason for the reduced catalytic activity of Ni-RM/HAC.

**Figure 13:**
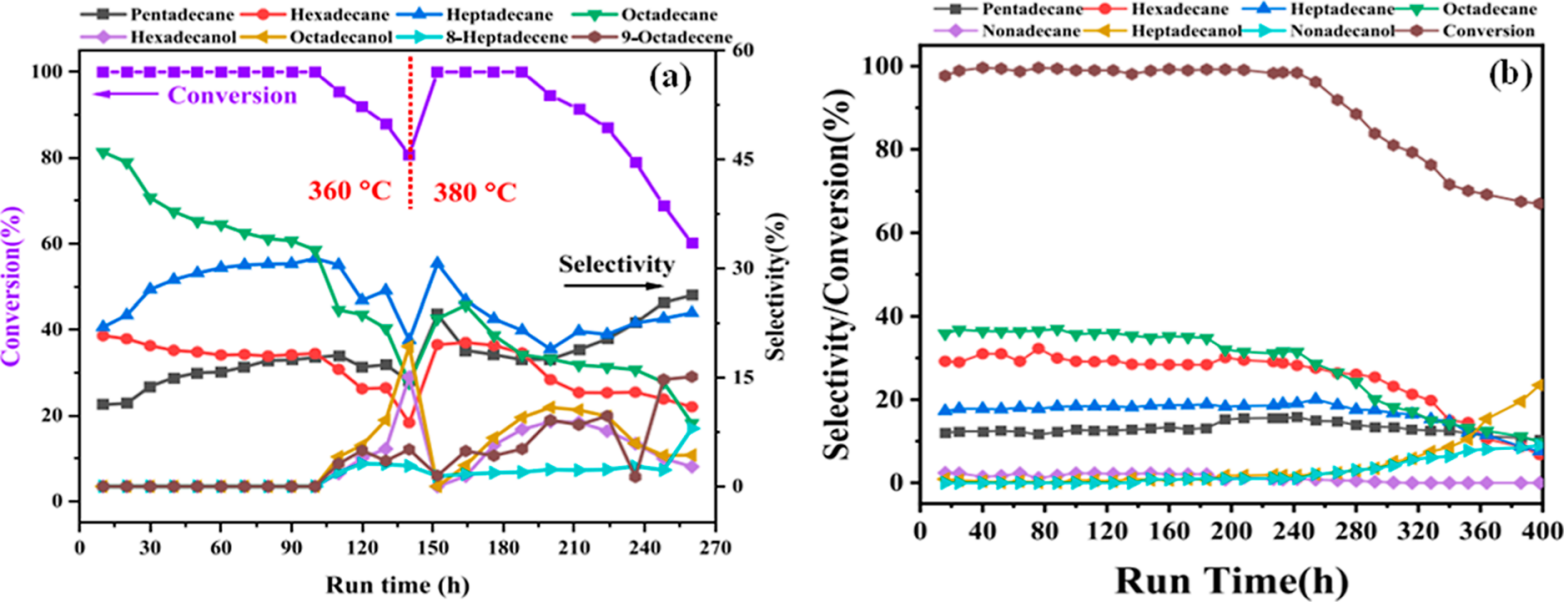
(a) Stability test of Ni-Mo/RM. Copyright © 2022 Elsevier Science Ltd. All rights reserved. (b) Ni/RM-HAC (reaction conditions: H_2_ pressure = 4 MPa, WHSV = 0.6 h^–1^ and H/O ratio = 1000 N mL mL^–1^), reproduced with permission.^240,241^ Copyright © 2023 Elsevier Science Ltd.

In the case of Ni-RM-500 catalyst (Figure 14a–c),^242^ deactivation due to pore blockage by carbon deposition, decreased surface area, Ni^0^ and Fe^0^ oxidation, and sintering over a reaction period of 135 h was observed. Similarly, particle agglomeration due to sintering was observed in the case of various RM-derived catalysts (Ni-N/RM, Ni-Cl/RM, Ni-AC/RM, and Ni-C/RM). The Ni-N/RM catalyst with well-dispersed Ni showed the highest activity and stability among others due to low sintering and agglomeration.^244^ In the case of slag-derived catalyst, the Ni/CGS catalyst used for the hydrotreatment of benzyl phenyl ether (a lignin model compound) showed a decrease in the catalytic activity after three consecutive cycles (Figure 14f) due to Ni particle agglomeration during the reaction (Figure 14d,e).^248^

**Figure 14:**
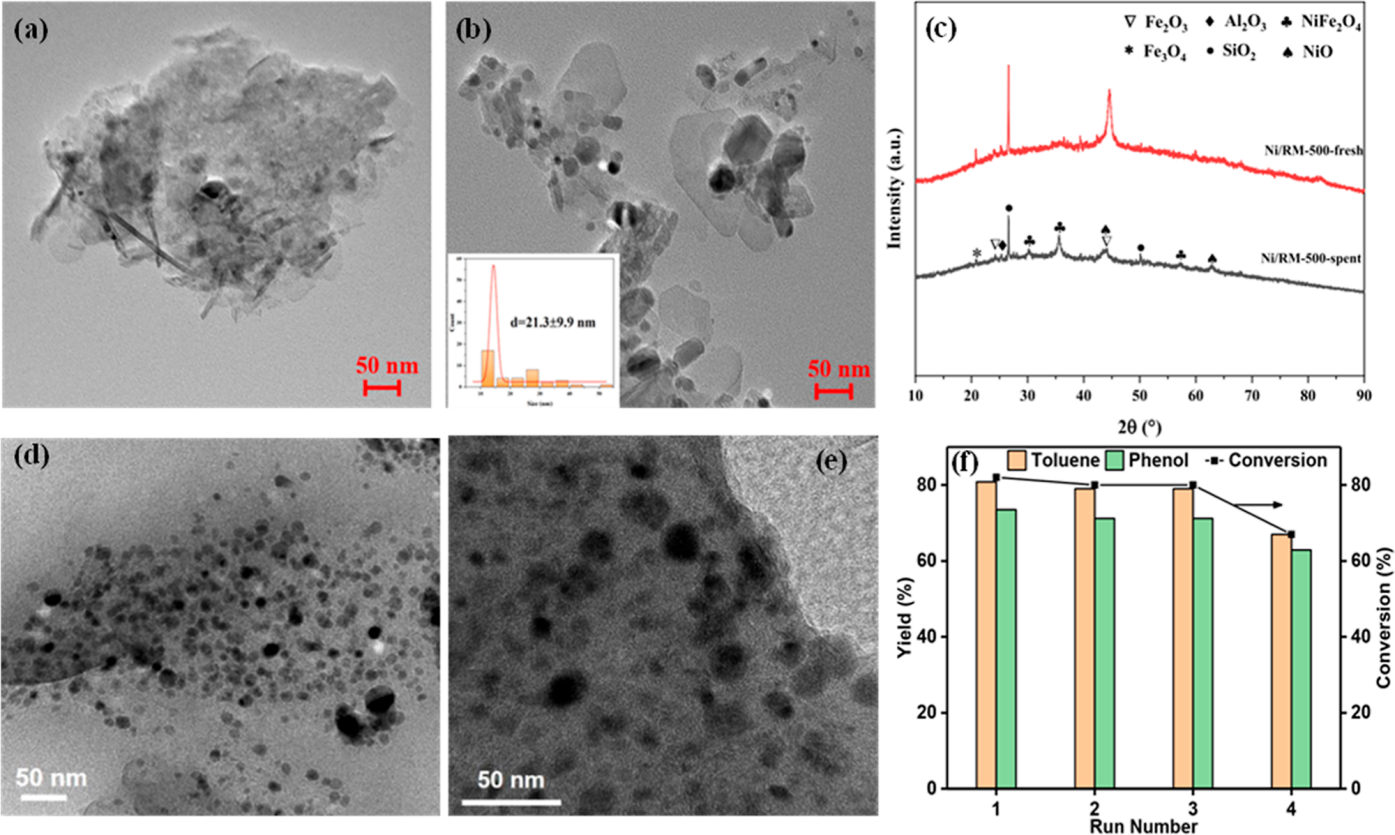
(a) TEM of Ni/RM-500-fresh and (b) Ni/RM-500-spent catalysts. (c) XRD patterns of fresh and spent Ni/RM-500, Copyright © 2022 Elsevier Science Ltd. All rights reserved. (d) TEM images of 10%Ni/CGS and (e) spent 10%Ni/CGS, and (f) reusability of 10%Ni/CGS on BPE conversion, reproduced with permission.^242,248^ Copyright © 2024 Elsevier Science Ltd.

Various metals supported by carbon were reported to have appreciable stability during the hydrotreatment of the bio-oil. The Ni/HPC3-700 catalyst showed a consistent bio-oil yield over three consecutive cycles. A sharp decrease in hydrocarbon content from ∼39% to ∼14% in the product demonstrated its continuous decline in HDO activity.^250^ The decreased activity over numbers of reuses was attributed to coke accumulation in the porous catalyst structure, blocking the active site access to reactants. Similar observations on catalyst deactivation due to pyrolytic carbon deposition on Ni sites for the NiMo-carbon catalyst,^251^ leading to decreased activity was also reported by García-Rollan et al.^251^ Coke deposition (Figure 15a,b) leading to catalyst deactivation and decreased performance (Figure 15 c) was also observed in the case of Pt-carbon catalyst (Pt/CSACN3-700),^258^ and Co-Mo supported on DF biochar.^263^

**Figure 15:**
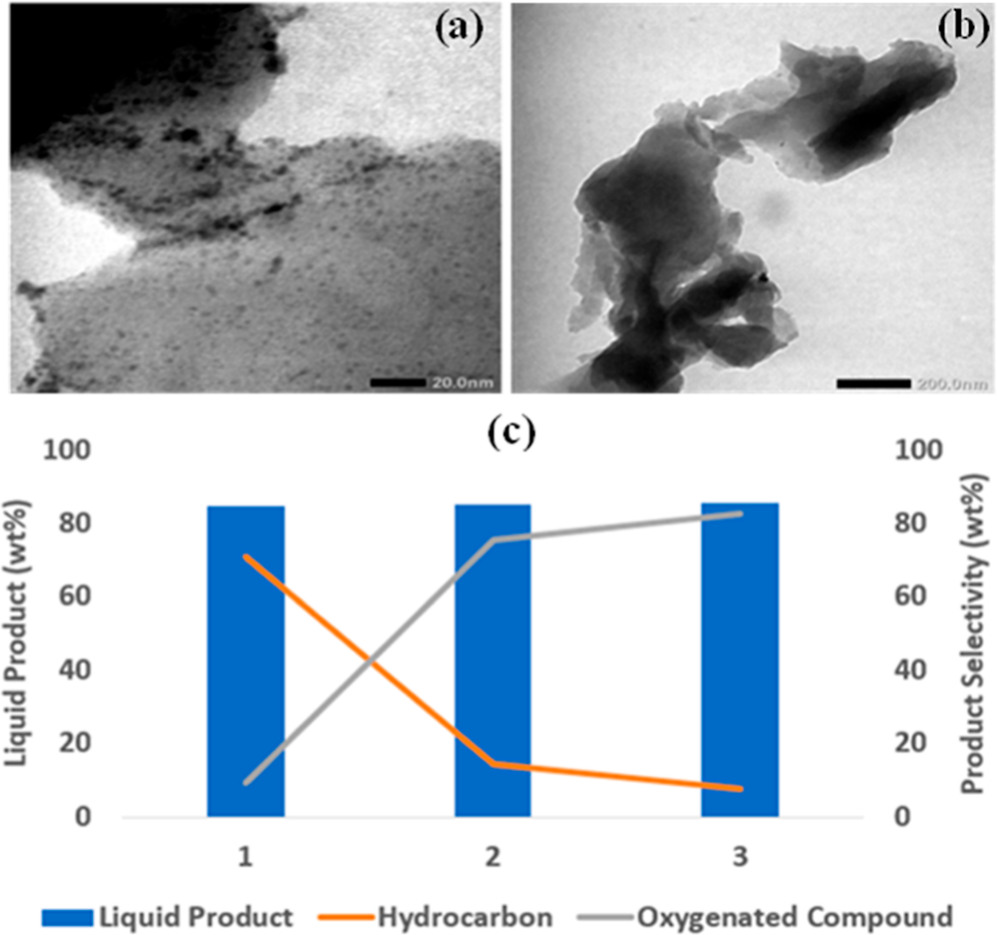
(a) Pore images of Pt/CSACN3-700 catalysts before and (b) after being used in the hydrotreatment of CIO and (c) the result of the liquid product obtained in the hydrotreatment of CIO over used Pt/CSACN3-700, reproduced with permission.^258^ Copyright © 2022 Elsevier Science Ltd.

On the other hand, the organic sulfides, nitrides, oxides, and metal impurities in WPPO are like those in crude oil.^268^ However, when chlorinated plastics like PVC are part of the feedstock, the chlorine content of WPPO is significantly higher than that of crude oil. Also, a significant portion of these contaminants in WPPOs can be partly linked to the additives used in plastic manufacture. A model oil of WPPO containing different impurities (dibenzothiophene, quinolone, chlorobenzene, ferric naphthenate, polymethylhydrosiloxane, and naphthenic acid) was used to study the deactivation mechanism of the Ni-based catalyst (5.0 wt % NiO and 26 wt % MoO_3_ supported on γ-Al_2_O_3_) via the hydrotreatment process in a fixed-bed at 320 °C and 4 MPa.^268^ The results showed that the presence of chlorides, nitrides, and sulfides in WPPO was associated with catalyst deactivation. It was found that during the upgrading process, the chloride compound conversion resulted in HCl gas release, which then reacted with the Ni active sites. This caused the size of the Ni nanoparticles to increase from 52.62 to 139.63 nm and the loss of 3–8% of the Ni active components, which specifically inhibited the catalyst’s hydrodesulfurization activity. This leads to catalyst deactivation due to chlorine poisoning,^268–270^ as illustrated in Figure 16. As a result of the facile mobility of Ni-Cl species, the NiCl_2_ aggregates easily, which consequently leads to loss of active metal Ni on the surface of the catalyst and reduction in catalytic activity. A similar deactivation due to restructuring of the Ni sites has also been reported for the Ni/SiO_2_ catalyst.^269^ However, it has been observed that in a bimetallic NiMg/Al_2_O_3_ catalyst, the basic MgO interacting preferentially with HCl delayed catalytic deactivation relative to monometallic Ni/Al_2_O_3_.^270^ Thus, MgO in most waste-derived catalysts could play a role in maintaining stability during upgrading reactions. As a result, the stability of waste-derived catalysts varies significantly based on the mixed metal oxide composition (e.g., alkali, rare earth metals, etc.), pore network and structure, contaminants in the bio-oil and WPPO, and reaction conditions.

**Figure 16:**
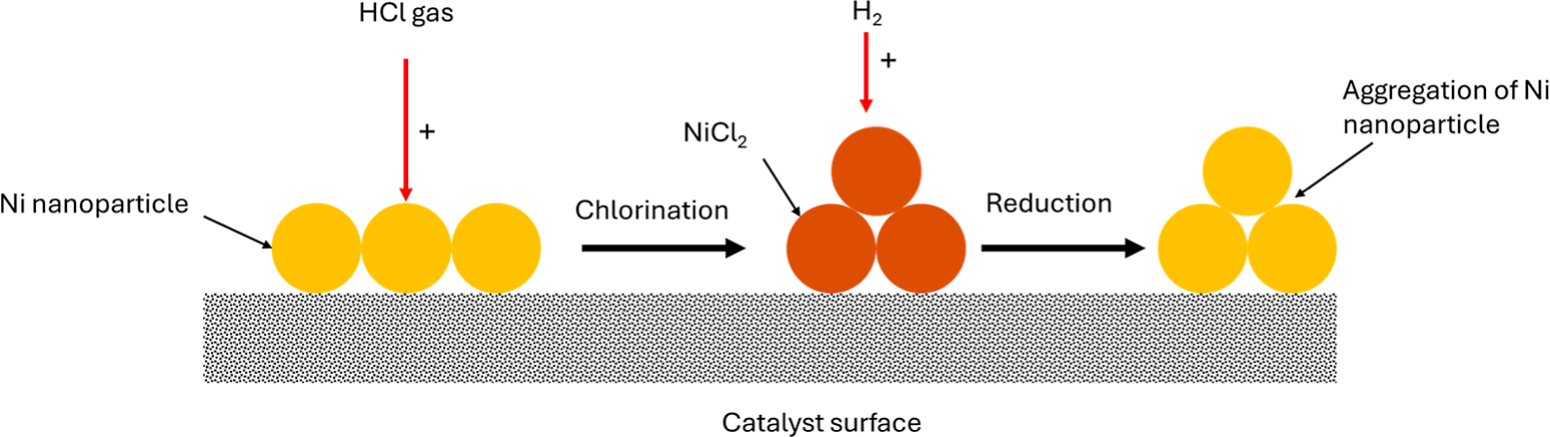
Deactivation due to chlorine poisoning of the catalyst. Adapted from ref 268, Copyright © 2022 Elsevier Science Ltd.

The following catalysts rapidly deactivate in the early hours during catalytic upgrading of chlorinated WPPOs ZnO and MgO in 5 h^216^ and Ni/SiO_2_ in 26 h.^271^ Additionally, the released HCl gas can potentially react with NH_3_ produced from the conversion of a nitride compound to generate NH_4_Cl, which could result in ammonium salts being crystallized in the catalyst bed. This contributes to the rapid deactivation of the catalyst. This implies that chloride compounds can cause rapid deactivation of catalysts. So, in addition to coke formation and deposition on the active metal sites, surface poisoning by HCl and/or metal sintering could occur. In a comparative study of the deactivation of the Ni/SiO_2_ and Ni_2_P/SiO_2_ via hydrodechlorination of chlorobenzene in a fixed bed at 300 °C, it was found that Ni_2_P particles exhibited good resistance to HCl poisoning and sintering.^271^ The trend in conversion of chlorobenzene with Ni_2_P/SiO_2_ declined as follows 94% (10 h), 90% (20 h), 41% (100 h), and 30% (200 h), whereas Ni/SiO_2_ was 30% (10 h), 40% (20 h), and 26% (26 h). This implies the phosphorus dopant improved the stability and resistance of the catalyst to chlorine poisoning due to the electron-deficiency of Ni^δ+^ (0 < δ < 1) site in Ni_2_P.^271^ HCl, the byproduct of chlorinated WPPO during catalytic upgrading, is a catalyst poison, reacting with metallic sites to form metal chloride. This decreases the electron density as an electron acceptor and restructures the metal.^269,271^ The presence of organic chloride in WPPOs strongly inhibited the HDS and HDN activity due to deactivation caused by chlorine poisoning.^268^ Therefore, for WPPO containing sulfide, nitride, and chloride, the competitive adsorption on the catalyst surface can rapidly deactivate the catalyst. For a feedstock containing nitride and chloride, the formation of ammonium salts (NH_4_Cl) strongly deactivates the catalyst.

The redox cycle of Fe_2_O_3_ found in RM promotes dechlorination reactions by acting as an electron shuttle, where the cycle between oxidation states Fe^2+^/Fe^3+^ facilitates the cleavage of chlorinated hydrocarbons. Consequently, differences in the structural properties of iron oxide phases present in the waste-derived catalyst can lead to different catalytic activity.^272^ For instance, hematite exhibited a higher catalytic dechlorination activity than ferrihydrite.^272^ During catalytic upgrading of WPPO with RM, most of the hematite (Fe_2_O_3_) structure could be converted to the magnetite (Fe_3_O_4_) structure, which exhibits superior affinity for Cl_2_ adsorption, leading to the formation of solid metal chlorides (FeCl_3_).^273^ Likewise, the alkaline components (Na, Ca, Mg, etc.) in the waste-derived catalyst can also react with the HCl gas to form solid metal chlorides. Additionally, RM-derived catalyst can effectively remove sulfur from WPPO by acting as a desulfurization catalyst with synergistic effects from its main active phase of Fe_2_O_3_ and other components, enhancing performance, but its active sites can be poisoned by S, causing catalytic performance decline.^274,275^

The regeneration and reusability of spent catalysts laden with formed and deposited coke and contaminants (e.g., Cl, N, NH_4_Cl, S, Si, etc.) will benefit industrial scale application and overall process economics. The regeneration of the spent 20 wt % α-Fe_2_O_3_/HY zeolite catalyst containing 3500 ppm Cl and about 6.8 wt % deposition of soft coke by combustion in air at 700 °C for 4 h showed that chlorine and coke contents were completely removed and activity restored.^196^ The performance of the regenerated 20 wt % α-Fe_2_O_3_/HY zeolite catalyst in upgrading WPPO in a fixed bed reactor at 450 °C produced 16.7 wt % (gasoline) and 53.0 wt % (kerosene/diesel) against the fresh counterpart 20.3 wt % (gasoline) and 52.3 wt % (kerosene/diesel). Consequently, the evaluation of different regeneration methods for spent zeolites used for the dechlorination of WPPO showed that oxidative regeneration at 600 °C restored the initial activity of the catalyst relative to thermal desorption under an inert atmosphere at 500 °C and air combustion at 450 °C.^276^ Moreover, oxidative regeneration of spent 12.5 wt % WO_*x*_/Al_2_O_3_ catalyst at 550 °C for 15 h demonstrated a performance comparable to the fresh counterpart with 95.1% a reduction in Cl content, matching that of fresh catalyst (96.5%) for catalytic upgrading of WPPO containing 428 ppm of Cl.^197,200^ Thus, the oxidative regenerated catalyst can perform comparatively better than a fresh catalyst. However, scaling up biochar-based catalytic upgrading of bio-oil and WPPO to industrial levels poses a regeneration challenge of heating to combust contaminants or deposited coke. Other regeneration techniques like steam, supercritical fluid extraction, and chemical face the challenges of solvent cost and being energy-intensive.

Scaling catalytic upgrading of bio-oil and WPPO to industrial levels poses several challenges and requires robust and low-cost catalysts capable of handling the heterogeneity of biomass/waste plastic feedstocks and varied operational conditions. Thus, the utilization of readily available industrial solid waste materials can reduce the need for costly chemical reagents for the fabrication of catalysts. Though this reduces, reuses, and recycles solid waste for both economic and environmental benefits, the industrial scale-up of waste-derived catalysts has challenges associated with safety risks, solvent/chemical, energy, and solid waste pretreatment costs. Consequently, wastewater from the synthesis of waste-derived catalysts could contain heavy metals and other pollutants that, if not treated before disposal, can cause major environmental pollution. However, in a comparative cost analysis between commercial zeolite and industrial solid waste-derived zeolites, it was shown that the cost of a waste-derived catalyst can be significantly lower than its commercial counterpart.^193^ Thus, moving from lab-scale to industrial production can be challenging and requires optimization of the pretreatment and synthesis processes to maintain economic viability. Additionally, technoeconomic analysis will play a crucial role in the industrial production and application of waste-derived catalysts. Although the performance and regeneration techniques of waste-derived catalysts vary greatly depending on the waste material and application, they can exhibit regeneration comparable to that of commercial catalysts. However, the pivotal differences typically lie in the regeneration process, energy costs, and presence of impurities.

## 5 Future Perspectives

The highlighted progress includes frontiers in the synthesis of heterogeneous catalysts from a broad range of industrial and geological waste materials and applications in the catalytic upgrading of biomass pyrolysis bio-oils and PPOs. The key challenges associated with waste-derived catalysts include raw material heterogeneity, scalability, and long-term stability.^277,278^ The impurities in natural materials and waste-derived heterogeneous catalysts can significantly affect their catalytic activity, selectivity, and lifespan, leading to reduced efficiency. These impurities have varying effects on the activities of the waste-derived catalyst and can be categorized as chemical, physical, or structural. For the efficient recycling of industrial waste in heterogeneous catalysts and waste management, it is crucial to understand, control, and remove undesirable impurities. Hence, methods of purification and removal of noncatalytically active impurities from the heterogeneously mixed solid waste that will improve performance need to be explored. As a result, future research should include multifunctional catalyst design. Instead of single-function materials, research should focus on designing catalysts from waste streams that can perform multiple tasks simultaneously, for example, using the inherent Fe-Ca-Al composition of RM for combined deoxygenation, deacidification, and tar cracking. To reduce carbon footprints and minimize environmental pollution, future research should focus on low-energy modification procedures and green preparation techniques. Optimization of pretreatment processes, such as acid modification to tailor composition, calcination, and reduction conditions, can be considered in transforming the parent waste material into a more effective catalyst. In addition, further research is required on waste-derived catalyst activation and deactivation mechanisms as well as long-term stability and regeneration processes.

Solid waste and natural clay materials are multicomponent in nature, with inert materials contributing to low crystallinity and impurity concentration (e.g., CaO, MgO, etc.), which can have a substantial impact on waste-derived catalyst synthesis, structure, physicochemical properties, and catalytic performance. Based on the unpredictability in the composition of solid waste materials as well as inconsistencies in bio-oil and WPPO compositions, future research should focus on techniques and strategies for reducing the impact of composition variability on waste-derived catalyst activity for process reliability and scalability. A standardized pretreatment of industrial solid waste materials (e.g., calcination at 600–1000 °C, acid washing, hydrothermal treatment with alkaline solution, etc.) can be investigated to reduce the impact of impurities and concentrate active components for a sustainable recycling strategy of waste-derived catalysts for industrial-scale applications. Pretreatment of raw waste materials with chemicals (acid or alkali) can leach certain impurities, improve the pore structure, and create active sites. In the formulation of waste-derived catalysts, alloying with other materials or impregnating with other metals can be standardized to improve their crystal structure, as well as activity and stability. Currently, waste-derived catalysts are prepared using trial-and-error techniques that must be optimized and standardized into a more cost-effective, green, and customized synthesis approach from raw solid waste composition. Hence, artificial intelligence (AI) and machine learning (ML) techniques can be useful tools in optimizing the pretreatment phase and standardizing the synthesis of catalysts from industrial solid waste and clay for industrial applications. Furthermore, advanced manufacturing and structuring aimed at bridging the lab-to-industry gap should be explored. Thus, transforming waste-derived catalysts into industrially relevant forms, such as pellets, extrudates, or monoliths, will promote industrial scale applications. This will improve the mechanical strength and reactor fluid dynamics, which are critical for large-scale operations. The application of predictive modeling and AI tools would facilitate the effect of the waste composition and treatment/purification processes on the catalyst products. The application of ML techniques would facilitate the development of synthesis–property–structure–function relationships. Also, to tackle feedstock heterogeneity, ML models will predict catalytic performance based on the variable composition of waste streams, enabling rapid screening and the custom formulation of catalysts for specific pyrolysis feeds.^105^ Consequently, research should explore the synergy between waste catalysts and novel reactor technologies. For instance, using microwave or induction heating to selectively heat magnetic catalysts (e.g., from RM or e-waste) could dramatically improve energy efficiency and unlock new reaction pathways.^279^

Biomass pyrolysis oil and WPPO can reduce the demand for crude oil and other fossil fuels, contributing to energy security and a lower carbon footprint. Catalytic upgrading of this pyrolysis oil would play a crucial role in closing the circular economy loop for plastics and biomass, minimizing waste, and promoting resource efficiency. Catalysts play a pivotal role in influencing the selectivity and quality of products, and future research should explore new catalysts and reaction conditions. For process simulation and optimization, reactor design and configuration, product distribution, efficient production, and evaluation of catalyst deactivation due to coke, research on the kinetic modeling of the catalytic upgrading of bio-oil and WPPO will be crucial. A study on the kinetic modeling of coprocessing of WPPO and vacuum gas oil will facilitate the integration into the existing refinery process. In addition, considerable efforts should be devoted to developing more efficient and cost-effective technologies for upgrading pyrolysis oil. The future of WPPO catalytic upgrading relies on enhancing their quality and expanding the development and applications of low-cost and sustainable catalysts. The detrimental effects of impurities on waste-derived catalysts can be reduced by understanding their types, sources, and impacts on the catalytic upgrading of WPPOs. This is pivotal in the development of appropriate catalysts and strategic purification techniques. Generally, this will result in more effective and sustainable solid waste recycling and management. The applications of ML techniques in further research will accelerate optimization of upgrading processes to ensure consistent product quality and address challenges related to scaling up production and management of feedstock variability. The synergistic effects of co-processing of biomass pyrolysis bio-oil and WPPO require investigation in terms of improving oil quality and reducing the need for external hydrogen. Practical economic benefits for chemically recycling waste plastics into WPPO and value-added chemicals can be obtained via catalytic upgrading conditions optimization and carrying out an extensive analysis of factors like feedstock, energy consumption, and the economic value of products.^28^ Consequently, to prove that catalytic upgrading of WPPO is feasible and economically viable, pilot-scale and industrial-scale production facilities and technoeconomic analysis are required.

Waste-derived catalysts from RM, slag, fly ash, and various carbon-based substances offer sustainable and cost-effective alternatives to conventional catalysts for bio-oil and WPPO upgrading. While notable advancements, particularly with Ni, Co, and Mo-based catalysts supported on these wastes, have been made, several critical concerns exist with them. For instance, the catalyst deactivation mechanisms of a few red-mud-based catalysts have not been explored in sufficient detail. Studies on slag and fly ash-derived catalysts in the context of bio-oil and WPPO hydrotreatment are highly limited, and a few studies involving fly ash and slag lack stability and regeneration investigation. Research on waste-derived carbon-based catalysts is reasonable; still, investigations on their long-term performance and reusability remain unexplored in some cases. Furthermore, while Ni as an active metal on various waste catalytic support has been studied extensively, the catalytic potential of other transition metals on these supports requires exploration, along with a deeper investigation of their activity and stability. In addition, research focusing on understanding and enhancing catalyst activity and stability through advanced synthesis and surface modification techniques is essential to advance waste-derived catalysts toward scalable industrial bio-oil upgrading applications.

Scaling-up the waste-derived catalyst production and applications for oil upgrading can face various limitations. For instance, impurity (e.g., Cl^–^, S^2–^, Na^+^, Ca^2+^, etc.) pretreatment in wastes may increase operating cost due to multiple washing, acidification, and drying steps. Robust catalyst design with high resistance potential to contaminants in WPPO and oxygenates in bio-oil through incorporating metals other than Ni, Co, and Mo, which are underexplored, could be considered in further research. Moreover, contaminants/oxygenates, coke deposition, and sintering reduce the catalyst stability through structural collapse of multimetal oxides waste-derived catalysts due to interaction with inherent impurities. Tailoring catalysts with mesoporous structure to promote coke burnoff and stable mixed oxides to prevent sintering could help minimize the issue. Industrial solid waste heterogeneity could also create reproducibility issues when applied to upgrading processes, such as hydrotreating. Hence, developing standardized pretreatment protocols, contaminants, and coke resistant catalysts synthesis methods along with their techno-economic evaluations would be crucial to make industrial waste catalysts feasible in the future.

## 6 Conclusion

This study addresses the increasing environmental and ecological problems caused by industrial and plastic waste, in line with global programs promoting sustainable waste and energy management. It promotes a paradigm shift toward a more sustainable and resource-efficient future by converting natural materials, industrial and mining waste into active catalysts, mixed waste plastics into oil, and agricultural and forestry residues into bio-oil. The review advances fuel recovery through catalytic upgrading of plastic and biomass pyrolysis oil and bio-oil utilizing waste-derived heterogeneous catalysts. The presence of several valuable metals in industrial wastes, such as Si, Al, Fe, and Ti, makes them a suitable source of catalysts for fuel production. Recycling industrial and geological waste materials through the synthesis of catalysts promotes cost-effectiveness and sustainability while addressing solid waste management strategies and environmental pollution.

First, the potential of waste-derived heterogeneous catalysts, offering a pathway to a sustainable and cost-effective approach to catalyst development, particularly in catalytic upgrading of biomass pyrolysis bio-oils and WPPOs, has been proven. The cost of commercialization and industrial applications could be decreased by using a combination of simple post-treatment techniques to substitute synthetic heterogeneous catalysts with recyclable and renewable solid waste materials. It was found that waste-derived catalysts have played a crucial role in improving the quality of pyrolysis products and yields (i.e., plastic oil and bio-oil) and process efficiency. Catalytic upgrading converts crude bio-oil and plastic oil, produced from biomass or waste plastic pyrolysis, into quality fuels by reducing the oxygen content and other contaminants and improving their stability and calorific value. Waste-derived heterogeneous catalysts were found to function similarly to traditional industrial catalysts, such as zeolite-based and hydrotreating catalysts, in the upgrading of bio-oil and PPOs. Hence, waste plastic has the potential to become a significant resource for producing alternative fuel. However, the development of waste-derived heterogeneous catalysts relies on experimental trial and error, because it involves complex interactions of multiple components. The heterogeneity of their structure due to multiple components makes it difficult to predict the activity. Therefore, the application of ML could systematize and standardize pretreatment and synthesis processes and accelerate catalyst development from industrial solid waste and low-cost natural materials. This would be in the form of a compositional structure–activity model to predict functional outcomes.

Upgraded bio-oil or plastic oil can then be used as a blend or direct substitute for petroleum-derived fuels in the existing infrastructure. This work promotes several UN SDGs, including SDG-7 (affordable and clean energy), SDG-12 (responsible consumption and production), and SDG-13 (climate action). However, the challenges in feedstock heterogeneity and scalability remain; the path forward involves embracing the complexity of industrial waste materials. Thus, for large-scale applications, the raw material compositions, impurities, and purification challenges need to be addressed. Thus, to lessen the environmental impact of plastics and solid waste from industrial and geological processes, pyrolysis for fuel or petrochemical feedstock must be scaled up, as should the use of waste-derived heterogeneous catalysts. However, advanced characterization techniques, process optimization, and developing a regeneration strategy can offer tailored solutions that address specific deactivation issues with waste-derived catalysts during the upgrading of bio-oil or plastic oil.

## References

[1] Jovičević-Klug M., Souza Filho I. R., Springer H., Adam C., Raabe D. (2024). Green Steel
from Red Mud through Climate-Neutral Hydrogen Plasma Reduction. Nature.

[2] Karimi Z., Rahbar-Kelishami A. (2023). Efficient Utilization of Red Mud
Waste via Stepwise
Leaching to Obtain α-Hematite and Mesoporous γ-Alumina. Sci Rep.

[3] Lanzerstorfer C. (2018). Fly Ash from
Coal Combustion: Dependence of the Concentration of Various Elements
on the Particle Size. Fuel.

[4] Pineda-Vásquez T., Rendón-Castrillón L., Ramírez-Carmona M., Ocampo-López C. (2024). From E-Waste
to High-Value Materials: Sustainable Synthesis
of Metal, Metal Oxide, and MOF Nanoparticles from Waste Printed Circuit
Boards. Nanomaterials.

[5] WorldSteel . Steel Industry Co-Products; World Steel Association, 2021. https://worldsteel.org/wp-content/uploads/Fact-sheet-Steel-industry-co-products.pdf.

[6] Ryabchuk P., Anwar M., Dastgir S., Junge K., Beller M. (2021). From Mobile
Phones to Catalysts: E-Waste-Derived Heterogeneous Copper Catalysts
for Hydrogenation Reactions. ACS Sustain Chem
Eng.

[7] Bennett J. A., Wilson K., Lee A. F. (2016). Catalytic
Applications of Waste Derived
Materials. J Mater Chem A Mater.

[8] Kabeyi M. J. B., Olanrewaju O. A. (2023). Review
and Design Overview of Plastic
Waste-to-Pyrolysis Oil Conversion with Implications on the Energy
Transition. Journal of Energy.

[9] Plastics Europe . Plastics-The Facts 2022; Brussels, 2022. https://plasticseurope.org/wp-content/uploads/2023/03/PE-PLASTICS-THE-FACTS_FINAL_DIGITAL-1.pdf (accessed Oct 7, 2024).

[10] OECD . Global Plastics Outlook Economic Drivers, Environmental Impacts and Policy Options, 2022. https://www.oecd.org/content/dam/oecd/en/publications/support-materials/2022/02/global-plastics-outlook_a653d1c9/Global%20Plastics%20Outlook%20I.pdf.

[11] Nayanathara
Thathsarani Pilapitiya P. G. C., Ratnayake A. S. (2024). The World
of Plastic Waste: A Review. Cleaner Mater..

[12] Vityuk, A. ; Norouzi, S. Purifying and Upgrading of Waste Plastics Pyrolysis Oils, 2023. https://www.digitalrefining.com/article/1002944/purifying-and-upgrading-of-waste-plastics-pyrolysis-oils.

[13] Faisal F., Rasul M. G., Jahirul M. I., Schaller D. (2023). Pyrolytic Conversion
of Waste Plastics to Energy Products: A Review on Yields, Properties,
and Production Costs. Sci. Total Environ..

[14] Peng Y., Wang Y., Ke L., Dai L., Wu Q., Cobb K., Zeng Y., Zou R., Liu Y., Ruan R. (2022). A Review on Catalytic Pyrolysis of Plastic Wastes to
High-Value Products. Energy Convers Manag.

[15] Kusenberg M., Zayoud A., Roosen M., Thi H. D., Abbas-Abadi M. S., Eschenbacher A., Kresovic U., De Meester S., Van Geem K. M. (2022). A Comprehensive
Experimental Investigation of Plastic
Waste Pyrolysis Oil Quality and Its Dependence on the Plastic Waste
Composition. Fuel Process. Technol..

[16] Miandad R., Barakat M. A., Aburiazaiza A. S., Rehan M., Ismail I. M. I., Nizami A. S. (2017). Effect of Plastic
Waste Types on Pyrolysis Liquid Oil. Int Biodeterior
Biodegradation.

[17] Mujtaba M., Fernandes Fraceto L., Fazeli M., Mukherjee S., Savassa S. M., Araujo
de Medeiros G., do Espírito Santo
Pereira A., Mancini S. D., Lipponen J., Vilaplana F. (2023). Lignocellulosic
Biomass from Agricultural Waste to the Circular Economy: A Review
with Focus on Biofuels, Biocomposites and Bioplastics. J Clean Prod.

[18] Mishra R., Kumar A., Singh E., Kumar S. (2023). Recent Research Advancements
in Catalytic Pyrolysis of Plastic Waste. ACS
Sustain Chem Eng.

[19] Hart A., Shah A., Leeke G., Greaves M., Wood J. (2013). Optimization
of the CAPRI Process for Heavy Oil Upgrading: Effect of Hydrogen and
Guard Bed. Ind. Eng. Chem. Res..

[20] Hart A., Onwudili J. A. (2025). Tuning the Activity of NbOPO4 with NiO for the Selective
Conversion of Cyclohexanone as a Model Intermediate of Lignin Pyrolysis
Bio-Oils. Energies (Basel).

[21] Das P. (2025). A Review on
the Catalytic Upgradation of Vegetable/Pyrolysis Bio-Oil from Renewable
Sources: Kinetic Studies and Environmental Impact Assessment. Catal. Sci. Technol..

[22] Jeswani H., Krüger C., Russ M., Horlacher M., Antony F., Hann S., Azapagic A. (2021). Life Cycle Environmental
Impacts of Chemical Recycling via Pyrolysis of Mixed Plastic Waste
in Comparison with Mechanical Recycling and Energy Recovery. Sci. Total Environ..

[23] Anwar M., Konnova M. E., Dastgir S. (2025). Circular Plastic
Economy for Sustainable
Development: Current Advances and Future Perspectives. RSC Sustainability.

[24] Makepa D. C., Chihobo C. H. (2024). Barriers to Commercial
Deployment of Biorefineries:
A Multi-Faceted Review of Obstacles across the Innovation Chain. Heliyon.

[25] Liaqat S., Sun Z., Zeng Y., Maeda N., Liu J. (2024). Technical Challenges
and Corrosion Research Progress in Bio-Crude Co-Processing. Chemical Engineering Journal.

[26] Sharifzadeh M., Sadeqzadeh M., Guo M., Borhani T. N., Murthy
Konda N. V. S. N., Garcia M. C., Wang L., Hallett J., Shah N. (2019). The Multi-Scale Challenges of Biomass Fast Pyrolysis and Bio-Oil
Upgrading: Review of the State of Art and Future Research Directions. Prog. Energy Combust. Sci..

[27] Zulkafli A. H., Hassan H., Ahmad M. A., Mohd
Din A. T., Wasli S. M. (2023). Co-Pyrolysis
of Biomass and Waste Plastics for Production of Chemicals and Liquid
Fuel: A Review on the Role of Plastics and Catalyst Types. Arabian J. Chem..

[28] Li S., Li Z., Zhang F., Chen J. (2024). Upgrading Waste Plastics to Value-Added
Aromatics. Chem Catal..

[29] Li F., Wang N., He X., Deng M., Yuan X., Zhang H., Nzihou A., Tsang D. C. W., Wang C.-H., Ok Y. S. (2025). Biochar-Based Catalytic Upgrading of Plastic Waste into Liquid Fuels
towards Sustainability. Commun Earth Environ.

[30] Qiu B., Yang C., Shao Q., Liu Y., Chu H. (2022). Recent Advances
on Industrial Solid Waste Catalysts for Improving the Quality of Bio-Oil
from Biomass Catalytic Cracking: A Review. Fuel.

[31] Biakhmetov B., Dostiyarov A., Ok Y. S., You S. (2023). A Review on
Catalytic
Pyrolysis of Municipal Plastic Waste. WIREs
Energy and Environment.

[32] Miandad R., Barakat M. A., Aburiazaiza A. S., Rehan M., Nizami A. S. (2016). Catalytic
Pyrolysis of Plastic Waste: A Review. Process
Saf. Environ. Prot..

[33] Palanivelrajan A. R., Feroskhan M. (2023). Comparison
of Various Catalysts in Pyrolysis Process:
A Review. Mater. Today: Proc..

[34] Fadillah G., Fatimah I., Sahroni I., Musawwa M. M., Mahlia T. M. I., Muraza O. (2021). Recent Progress in Low-Cost Catalysts
for Pyrolysis
of Plastic Waste to Fuels. Catalysts.

[35] Qiu B., Deng N., Zhang Y., Wan H. (2018). Application of Industrial
Solid Wastes in Catalytic Pyrolysis. Asia-Pac.
J. Chem. Eng..

[36] Lahijani P., Mohammadi M., Mohamed A. R., Ismail F., Lee K. T., Amini G. (2022). Upgrading
Biomass-Derived Pyrolysis Bio-Oil to Bio-Jet Fuel through
Catalytic Cracking and Hydrodeoxygenation: A Review of Recent Progress. Energy Convers Manag.

[37] Vuppaladadiyam A. K., Vuppaladadiyam S. S. V., Awasthi A., Sahoo A., Rehman S., Pant K. K., Murugavelh S., Huang Q., Anthony E., Fennel P., Bhattacharya S., Leu S.-Y. (2022). Biomass Pyrolysis:
A Review on Recent Advancements and Green Hydrogen Production. Bioresour. Technol..

[38] Yang S., Qian B., Wang Y., Taira K., Zhou Q., Wilson K., Lee A. F., Zhang L. (2023). Fly Ash Waste-Derived
Fe@Fe3O4 Core-Shell Nanoparticles for Acetic Acid Ketonization. Appl. Catal., B.

[39] Yang X., Zhang J., Zheng J., Liu Z., Liu J., Li S., Ye Y., Xie W., Fan J., Lan H., Wang D., Zheng Z. (2023). In-Situ and Ex-Situ
Catalytic Pyrolysis
of Cellulose to Produce Furans over Red Mud-Supported Transition Metal
Catalysts. J. Anal. Appl. Pyrolysis.

[40] Bhatt A., Priyadarshini S., Acharath Mohanakrishnan A., Abri A., Sattler M., Techapaphawit S. (2019). Physical,
Chemical, and Geotechnical
Properties of Coal Fly Ash: A Global Review. Case Studies in Construction Materials.

[41] Du P., Wang P., Zhang X., Wen G., Wang Y. (2024). Properties,
Hazards and Valuable Metal Recovery Technologies of Red Mud: A Review. Particuology.

[42] Tomczyk A., Sokołowska Z., Boguta P. (2020). Biochar Physicochemical Properties:
Pyrolysis Temperature and Feedstock Kind Effects. Rev Environ Sci Biotechnol.

[43] de
Souza dos Santos G. E., Oliveira C. C., Moura L. G., Hori C. E., de Souza Barrozo M. A. (2025). From Brewers’ Waste to Fuel Precursors: Catalytic
Pyrolysis of BSG Using CaO and Nb2O5-Based Catalysts for Enhanced
Hydrocarbon Production. J. Anal. Appl. Pyrolysis.

[44] Seah C. C., Habib S. H., Hafriz R. S. R. M., Shamsuddin A. H., Salmiaton A. (2023). Can Waste Eggshell Replace Commercial
Zeolites as Catalyst
for Bio-Oil Production?. J. Anal. Appl. Pyrolysis.

[45] Yi L., Liu H., Xiao K., Wang G., Zhang Q., Hu H., Yao H. (2019). In Situ Upgrading of Bio-Oil via CaO Catalyst Derived
from Organic
Precursors. Proc. Combust. Inst..

[46] Vichaphund S., Sricharoenchaikul V., Atong D. (2017). Industrial Waste Derived CaO-Based
Catalysts for Upgrading Volatiles during Pyrolysis of Jatropha Residues. J. Anal. Appl. Pyrolysis.

[47] Yel E., Kalem M., Göktepeli G., Özgan Kurt A., Ahmetli G., Önen V. (2025). Catalytic Co-Pyrolysis of PET/PP
Plastics and Olive Pomace Biomass with Marble Sludge Catalyst. Turkish Journal of Analytical Chemistry.

[48] Mysore
Prabhakara H., Bramer E. A., Brem G. (2021). Role of Dolomite as
an In-Situ CO2 Sorbent and Deoxygenation Catalyst in Fast Pyrolysis
of Beechwood in a Bench Scale Fluidized Bed Reactor. Fuel Process. Technol..

[49] Kwon E. E., Yi H., Park J. (2012). Transesterification
of Used Vegetable Oil by Magnesium
Slag as Heterogeneous Catalyst (MgO-CaO/Al_2_O_3_). J. Chem. Eng. Jpn..

[50] Pratiwi R. G., Wantala K. (2022). Hydro-Conversion of Palm Oil via
Continuously Pyrolytic
Catalysis to Biofuels over Oxide-Based Catalyst Derived from Waste
Blood Clamshell: Effect of Magnesium Contents. Mol. Catal..

[51] Chen W., Li K., Xia M., Yang H., Chen Y., Chen X., Che Q., Chen H. (2018). Catalytic Deoxygenation Co-Pyrolysis of Bamboo Wastes
and Microalgae with Biochar Catalyst. Energy.

[52] Yang Y., Xiao P., Wen M., Liu T., Yang J., Dai S., Zhao Y., Huang Q., Liu Z., Li B. (2024). A Review on
the Modified Red Mud for Biomass Catalytic Pyrolysis: Preparation,
Mechanisms and Perspectives. J. Anal. Appl.
Pyrolysis.

[53] Tsai C.-H., Tsai W.-T. (2024). Sustainable Processes
Reusing Potassium-Rich Biomass
Ash as a Green Catalyst for Biodiesel Production: A Mini-Review. Processes.

[54] Lou J., Rezaee Babadi M., Otadi M., Tarahomi M., Van Le Q., Ali Khonakdar H., Li C. (2023). Agricultural Waste Valorization towards
(Nano)­Catalysts for the Production of Chemicals and Materials. Fuel.

[55] Li H., Niu S., Lu C., Liu M., Huo M. (2014). Use of Lime Mud from
Paper Mill as a Heterogeneous Catalyst for Transesterification. Sci. China: Technol. Sci..

[56] Fan W., Tahir M. H., Chen D., Hong L., Yin L., Yu H. (2024). High Quality Oil and H2-Rich Gas Production from Municipal Solid
Wastes through Pyrolysis and Catalytic Reforming: Comparison of Differently
Modified Waste Char-Based Catalysts. J. Anal.
Appl. Pyrolysis.

[57] Guo F., Liang S., Zhao X., Jia X., Peng K., Jiang X., Qian L. (2019). Catalytic Reforming
of Biomass Pyrolysis
Tar Using the Low-Cost Steel Slag as Catalyst. Energy.

[58] Li X., Huang Z., Shao S., Cai Y. (2023). Catalytic Pyrolysis
of Biomass to Produce Aromatic Hydrocarbons in a Cascade Dual-Catalyst
System: Design of Red Mud Based Catalyst Assisted by the Analysis
of Variance. J Clean Prod.

[59] Agblevor F. A., Wang H., Beis S., Christian K., Slade A., Hietsoi O., Santosa D. M. (2020). Reformulated Red
Mud: A Robust Catalyst for *In Situ* Catalytic Pyrolysis
of Biomass. Energy Fuels.

[60] Gu H., Wang N., Liu S. (2012). Radiological
Restrictions of Using
Red Mud as Building Material Additive. Waste
Management & Research: The Journal for a Sustainable Circular
Economy.

[61] Moise, G. ; Capota, P. ; Enache, L. ; Neagu, E. ; Dragut, V. ; Mihaescu, D. ; Mara, L. ; Chirea, A. ; Zavoianu, R. ; Sarbu, A. MATERIAL COMPOSITION AND PROPERTIES OF RED MUD COMING FROM DOMESTIC ALUMINA PROCESSING PLANT. In SIMI 2017; National Research and Development Institute for Industrial Ecology, 2017; pp 279–289.

[62] Ju F., Wu T., Wang M., Lin R., Zhao M., Ng S. H., Ling H. (2019). Effect of Nitrogen Compounds on Reactive Adsorption Desulfurization
over NiO/ZnO-Al2O3-SiO2 Adsorbents. Ind. Eng.
Chem. Res..

[63] Nokkosmäki M. I., Kuoppala E. T., Leppämäki E. A., Krause A. O. I. (2000). Catalytic
Conversion of Biomass Pyrolysis Vapours with Zinc Oxide. J. Anal. Appl. Pyrolysis.

[64] Małecki S., Gargul K. (2018). Low-Waste Recycling of Spent CuO-ZnO-Al2O3
Catalysts. Metals (Basel).

[65] Pan Y., Sima J., Wang X., Zhou Y., Huang Q. (2021). BTEX Recovery
from Waste Rubbers by Catalytic Pyrolysis over Zn Loaded Tire Derived
Char. Waste Manage..

[66] Namchot W., Jitkarnka S. (2016). Impacts of Nickel Supported on Different
Zeolites on
Waste Tire-Derived Oil and Formation of Some Petrochemicals. J. Anal. Appl. Pyrolysis.

[67] Ding K., Liu S., Huang Y., Liu S., Zhou N., Peng P., Wang Y., Chen P., Ruan R. (2019). Catalytic Microwave-Assisted
Pyrolysis of Plastic Waste over NiO and HY for Gasoline-Range Hydrocarbons
Production. Energy Convers Manag.

[68] Cho E. H., Kim K.-D., Yoon B. S., Cho E., Yu Y. J., Phan T. N., Jeon S.-G., Ko C. H. (2023). Efficient
Synthesis
of Nickel-Molybdenum/USY-Zeolite Catalyst for Eliminating Impurities
(N, S, and Cl) in the Waste Plastic Pyrolysis Oil: Dispersion Effect
of Active Sites by Surfactant-Assisted Melt-Infiltration. Catalysts.

[69] Guo D., Hu M., Chen Z., Cui B., Zhang Q., Liu Y., Luo S., Ruan R., Liu Y. (2020). Catalytic Pyrolysis of Rain Tree
Biomass with Nano Nickel Oxide Synthetized from Nickel Plating Slag:
A Green Path for Treating Waste by Waste. Bioresour.
Technol..

[70] Wang Y., Cui H., Song F., Tan H., Yi W., Zhang Y. (2022). Upgrading
Fast Pyrolysis Oil through Decarboxylation by Using Red Mud as Neutralizing
Agent for Ketones Production and Iron Recovery. ChemistrySelect.

[71] Eldeeb A. M., Aboutaleb W. A., Dhmees A. S., El Naggar A. M. A., Emara K., Elgendy A. T., Ahmed A. I. (2022). Bio-fuels Production
through Waste Tires Pyrolytic Oil Upgrading over Ni-W/Zeolite Composites
Derived from Blast Furnace Slag. Int. J. Energy
Res..

[72] Marhaini M., Fernianti D., Aulia M. R. (2024). Effective Pyrolysis of LDPE Plastic
Waste to Fuel Using Titanium Dioxide Catalyst. International Journal of ADVANCED AND APPLIED SCIENCES.

[73] Correa-Muriel D., Valencia-Sánchez H., Cortes-Hernández H., González-Vera D., Herrera J., Campos C. H., Casella M. L., Arteaga-Perez L. E., Osorio-Vargas P. (2022). Nickel and Cobalt Ilmenites-Based
Catalysts for Upgrading Pyrolytic Oil during Pyrolysis of Waste Tires. Catalysts.

[74] Grzeszczak J., Wróblewska A., Michalkiewicz B., Dzięcioł M., Janda-Milczarek K. (2025). Oxidation
of α-Pinene on the Ti-SBA-15 Catalyst
Obtained Using Orange Peel Waste as Components of the Synthesis Gel. Molecules.

[75] Xiao Y., Zhang J., Zhu L., Shan R., Yuan H., Chen Y. (2024). Electroplating Sludge-Derived
Magnetic Copper-Containing Catalysts
for Selective Hydrogenation of Bio-Based Furfural. Biomass Convers. Biorefin..

[76] Zhang C., Zhang L., Li Q., Wang Y., Liu Q., Wei T., Dong D., Salavati S., Gholizadeh M., Hu X. (2019). Catalytic Pyrolysis
of Poplar Wood over Transition Metal Oxides:
Correlation of Catalytic Behaviors with Physiochemical Properties
of the Oxides. Biomass Bioenergy.

[77] Moonsrikaew W., Duangchan A. (2022). Deoxygenation
of Pyrolysis Vapor from Palm Fruit Cake
over NiMo/γ-Al2O3 Catalyst: Effect of CeO2, TiO2, and ZrO2 Additives. Mol. Catal..

[78] Lee H. W., Kim Y.-M., Jeong C.-S., Park S. H., Kim J. M., Jung S.-C., Kim S. C., Jeon J.-K., Park Y.-K. (2017). Catalytic
Pyrolysis of Waste Wood Plastic Composite Over H-V-MCM-41 Catalysts. Sci. Adv. Mater..

[79] Zolfagharpour H. R., Sharafati A., Hosseinzadeh M. (2024). Effect of V2O5 Nanoparticles and
Temperature on the Chemical Compounds of Bio-Oils in Catalytic Pyrolysis
of Sugarcane Bagasse. Biomass Convers. Biorefin..

[80] Wang J., Xi Z., Niu B., Gao R., Xu Z. (2024). Catalytic Pyrolysis
of Waste-Printed Circuit Boards Using a Cu/Fe Bimetal Synergistic
Effect to Enhance Debromination. Sustainability.

[81] Hoseini S., Rahemi N., Allahyari S., Tasbihi M., Ghareshabani E. (2020). Effect of
Hydrometallurgical Process Parameters on the Mn2O3 Nano Catalysts
Derived from Spent Batteries Used in the Plasma Catalytic Oxidation
of BTX. Adv. Powder Technol..

[82] Xu M., Shi Z., Zhu X., Lai Y., Xia A., Huang Y., Jiang X., He J., Zhou M., Zhu X., Liao Q. (2024). Ex-Situ Catalytic Upgrading
of Biomass Pyrolysis Volatiles over Thermal-Decomposition
Products of Spent Lithium-Ion Batteries for Bio-Oil Deoxygenation
and Hydrogen-Rich Syngas Production. Int. J.
Hydrogen Energy.

[83] Bao X., Zhang Z., Luo T., Wu X., Xie Z., Lan S., Xie S., Zhou D. (2020). Conversion
of Cerium and Lanthanum
from Rare Earth Polishing Powder Wastes to CeO2 and La0.6Ca0.4CoO3. Hydrometallurgy.

[84] Zou D., Li H., Deng Y., Chen J., Bai Y. (2021). Recovery of Lanthanum
and Cerium from Rare Earth Polishing Powder Wastes Utilizing Acid
Baking-Water Leaching-Precipitation Process. Sep. Purif. Technol..

[85] Kar B. B., Datta P., Misra V. N. (2004). Spent Catalyst:
Secondary Source
for Molybdenum Recovery. Hydrometallurgy.

[86] Ren X., Hai C., Li X., Shen Y., Zeng J., Zhou Y., Chen Y. (2017). Investigation
and Preparation of CeO2-TiO2/FA (Fly Ash) SCR Catalyst. Integr. Ferroelectr..

[87] Shanmuganathan R., Nguyen N. D., Al-Ansari M. M., Sathiyamoorthi E., Lee J., Priya S. D. (2024). Identification of
Suitable Catalyst among HZSM-5, HY
and γ-Al2O3 to Obtain Upgraded Pyrolysis Oil with Augmented
Liquid Oil Yield. Environ. Res..

[88] Melo J. A., de Sá M. S., Moral A., Bimbela F., Gandía L. M., Wisniewski A. (2021). Renewable Hydrocarbon Production
from Waste Cottonseed
Oil Pyrolysis and Catalytic Upgrading of Vapors with Mo-Co and Mo-Ni
Catalysts Supported on γ-Al2O3. Nanomaterials.

[89] Yoon B. S., Kim C., Park G.-J., Jeon S. G., Ko C. H. (2024). Upgrading Waste
Plastic Pyrolysis Oil via Hydrotreating over Sulfur-Treated Ni-Mo/Al2O3
Catalysts. Fuel.

[90] Chang G., Shi P., Guo Y., Wang L., Wang C., Guo Q. (2020). Enhanced Pyrolysis
of Palm Kernel Shell Wastes to Bio-Based Chemicals and Syngas Using
Red Mud as an Additive. J Clean Prod.

[91] Pragathi P., Elansezhian R., Magesh G., Velmurugan R. (2024). Utilizing
the Waste Fly Ash and Spent Catalyst Residue as Reinforcement for
the Development of Sustainable Composites and Investigating Its Mechanical
and Microstructure Behaviour. Mater. Chem. Phys..

[92] Kanduri P. K., Seethamraju S. (2023). In Situ and
Ex Situ Catalytic Co-Pyrolysis of Lignocellulosic
Biomass and Plastics (Low-Density and High-Density Polyethylene) Using
Spent FCC Catalyst. Waste Biomass Valorization.

[93] Kongngoen P., Phetwarotai W., Assabumrungrat S., Phusunti N. (2023). Possible Use of Spent
FCC Catalyst for Upgrading of Wax from the Pyrolysis of Plastics to
Liquid Fuel. J. Anal. Appl. Pyrolysis.

[94] Meena P., Singh S., Sharma M., Saharan V. K., George S., Bhoi R. (2024). Enhanced Oil Yield by Catalytic Pyrolysis
of Thermoplastics Using
Cost-Effective Spent FCC and BaCO3 and Its Valorization to Gasoline
and Diesel Grade Fuel via Fractionation. Korean
J. Chem. Eng..

[95] Montini D., Cara C., D’Arienzo M., Di Credico B., Mostoni S., Nisticò R., Pala L., Scotti R. (2023). Recent Advances
on Porous Siliceous Materials Derived from Waste. Materials.

[96] Thao B. T. T., Bui T.-V., Do Q.-D., Nguyen V. C., Kumar P. S., Vo D.-V. N., Hieu N. H., Pham T.-P. T., Yusuf M., Dang V. D., Al-Kahtany K., Nhiem L. T., Pham L. K. H. (2025). Facile
Preparation of Mesoporous Alumina-Silica Catalysts Derived from Rice
Husk Ash for Fast Catalytic Pyrolysis of Biomass. Chem. Eng. Res. Des..

[97] Shen Y., Zhao P., Shao Q. (2014). Porous Silica
and Carbon Derived
Materials from Rice Husk Pyrolysis Char. Microporous
Mesoporous Mater..

[98] Rangel M. do C., Mayer F. M., Carvalho M. da S., Saboia G., de Andrade A. M. (2023). Selecting
Catalysts for Pyrolysis of Lignocellulosic Biomass. Biomass.

[99] Hassan H., Hameed B. H. (2023). Green Hydroxyapatite-Zeolite Catalyst Derived from
Steel Waste as an Effective Catalyst for the Hydrocarbon Production
via Co-Catalytic Pyrolysis of Sugarcane Bagasse and High-Density Polyethylene. Catal. Commun..

[100] Kabir G., Mohd Din A. T., Hameed B. H. (2018). Pyrolysis
of Oil
Palm Mesocarp Fiber Catalyzed with Steel Slag-Derived Zeolite for
Bio-Oil Production. Bioresour. Technol..

[101] Wu R., Lv P., Wang J., Bai Y., Wei J., Song X., Su W., Yu G. (2023). Catalytic
Upgrading
of Cow Manure Pyrolysis Vapors over Zeolite/Carbon Composites Prepared
from Coal Gasification Fine Slag: High Quality Bio-Oil Obtaining and
Mechanism Investigation. Fuel.

[102] Sun K., Themelis N. J., Bourtsalas A. C., Huang Q. (2020). Selective Production
of Aromatics from Waste Plastic Pyrolysis by Using Sewage Sludge Derived
Char Catalyst. J Clean Prod.

[103] Obadiah A., Swaroopa G. A., Kumar S. V., Jeganathan K. R., Ramasubbu A. (2012). Biodiesel Production from Palm Oil Using Calcined Waste
Animal Bone as Catalyst. Bioresour. Technol..

[104] Tang S., Liang J., Xu X., Jin Y., Xuan W., Li O., Fang L., Li Z. (2023). Targeting
Phosphorus Transformation to Hydroxyapatite Through Sewage Sludge
Pyrolysis Boosted by Quicklime Toward Phosphorus Fertilizer Alternative
with Toxic Metals Compromised. Renewable and
Sustainable Energy Reviews.

[105] Chen C., Wang L., Xu Y., Wang F. (2024). Production
of Hydroxyapatite Utilizing Calcium and Phosphorus in Sewage Sludge
Incineration Ash. Process Saf. Environ. Prot..

[106] Mortensen P. M., Grunwaldt J.-D., Jensen P. A., Knudsen K. G., Jensen A. D. (2011). A Review of Catalytic
Upgrading of Bio-Oil to Engine
Fuels. Appl Catal A Gen.

[107] Marafi M., Stanislaus A. (2007). Studies on
Recycling and Utilization
of Spent Catalysts: Preparation of Active Hydrodemetallization Catalyst
Compositions from Spent Residue Hydroprocessing Catalysts. Appl. Catal., B.

[108] Linares N., Silvestre-Albero A. M., Serrano E., Silvestre-Albero J., García-Martínez J. (2014). Mesoporous Materials for Clean Energy
Technologies. Chem. Soc. Rev..

[109] Martínez C., Corma A. (2011). Inorganic Molecular
Sieves: Preparation,
Modification and Industrial Application in Catalytic Processes. Coord. Chem. Rev..

[110] Sudarsanam P., Zhong R., Van den Bosch S., Coman S. M., Parvulescu V. I., Sels B. F. (2018). Functionalised Heterogeneous
Catalysts for Sustainable Biomass Valorisation. Chem. Soc. Rev..

[111] Hart A., Wood J. (2025). Methodological Review of Zeolite
Synthesis from Industrial Waste and Natural Clays and the Fabrication
of Hierarchical Pore Structures. Next Materials.

[112] Hart A. (2023). Circular Economy: Closing the Catalyst Loop with Metal
Reclamation
from Spent Catalysts, Industrial Waste, Waste Shells and Animal Bones. Biomass Convers. Biorefin..

[113] Hart A., Ebiundu K., Peretomode E., Onyeaka H., Nwabor O. F., Obileke K. (2022). Value-Added Materials
Recovered from Waste Bone Biomass: Technologies and Applications. RSC Adv..

[114] Kanwal Q., Zeng X., Li J. (2023). Measuring the Recycling
Potential of Industrial Waste for Long-Term Sustainability. Humanit Soc Sci Commun.

[115] Li X., Sun J., Zhang H., Shao S., Cai Y. (2022). Enhanced Production
of Monocyclic Aromatic Hydrocarbons by Catalytic Pyrolysis of Rape
Straw in a Cascade Dual-Catalyst System of Modified Red Mud and HZSM-5. Fuel Process. Technol..

[116] Jahromi H., Agblevor F. A. (2018). Hydrotreating of
Guaiacol: A Comparative
Study of Red Mud-Supported Nickel and Commercial Ni/SiO2-Al2O3 Catalysts. Appl Catal A Gen.

[117] Santosa D. M., Zhu C., Agblevor F. A., Maddi B., Roberts B. Q., Kutnyakov I. V., Lee S. J., Wang H. (2020). In Situ Catalytic
Fast Pyrolysis Using Red Mud Catalyst: Impact of Catalytic Fast Pyrolysis
Temperature and Biomass Feedstocks. ACS Sustain
Chem Eng.

[118] Karimi E., Briens C., Berruti F., Moloodi S., Tzanetakis T., Thomson M. J., Schlaf M. (2010). Red Mud as a Catalyst
for the Upgrading of Hemp-Seed Pyrolysis Bio-Oil. Energy Fuels.

[119] Marlina E., Alhikami A. F., Mardani S. A., Trismawati T., Yazirin C. (2024). Catalytic Pyrolysis of Plastic Waste
Using Red Mud
and Limestone: Pyrolytic Oil Production and Ignition Characteristics. Automotive Experiences.

[120] Pal S. K., Prabhudesai V. S., Mohanty K., Vinu R. (2025). Red Mud as
a Potential Catalyst for Petrochemicals Production From Oxygenated
Aromatic Plastic Wastes via Fast Pyrolysis. ChemCatChem.

[121] Lopez-Urionabarrenechea A., de Marco I., Caballero B. M., Laresgoiti M. F., Adrados A. (2015). Upgrading of Chlorinated Oils Coming
from Pyrolysis of Plastic Waste. Fuel Process.
Technol..

[122] Tan Y. L., Hameed B. H., Abdullah A. Z. (2020). Deoxygenation
of
Pyrolysis Vapour Derived from Durian Shell Using Catalysts Prepared
from Industrial Wastes Rich in Ca, Fe, Si and Al. Sci. Total Environ..

[123] Mahdavianpour M., Pourakbar M., Alavi N., Masihi N., Mirzaei F., Aghayani E. (2023). Biodiesel
Production from Waste Frying
Oils in the Presence of Zeolite Synthesized from Steel Furnace Slag. Int. J. Environ. Anal. Chem..

[124] Deng N., Liu T., He G., Wang Q. (2022). Optimization
of Waste Paper’s Catalytic Cracking to Liquid Fuel Using Copper
Slag as the Catalyst Based on Response Surface Methodology. J. Anal. Appl. Pyrolysis.

[125] David E. (2023). Upgrading the Corn Cob Pyrolysis
Vapors by Processing over Catalysts
Based on Aluminium Slag and the Assessment of the Produced Bio-Oil. J. Anal. Appl. Pyrolysis.

[126] Vichaphund S., Wimuktiwan P., Soongprasit K., Sricharoenchaikul V., Atong D. (2025). Hydrocarbon Recovery
from On-Shore
Oil-Based Drill Cuttings (OBDCs) by Fast Pyrolysis over Waste-Derived
ZSM-5 and CaO Catalysts Using Py-GCMS. Process
Saf. Environ. Prot..

[127] Ro D., Shafaghat H., Jang S.-H., Lee H. W., Jung S.-C., Jae J., Cha J. S., Park Y.-K. (2019). Production of an Upgraded Lignin-Derived
Bio-Oil Using the Clay Catalysts of Bentonite and Olivine and the
Spent FCC in a Bench-Scale Fixed Bed Pyrolyzer. Environ. Res..

[128] Zhang H., Xiao R., Wang D., Zhong Z., Song M., Pan Q., He G. (2009). Catalytic
Fast Pyrolysis
of Biomass in a Fluidized Bed with Fresh and Spent Fluidized Catalytic
Cracking (FCC) Catalysts. Energy Fuels.

[129] Lahtinen J., Ohra-aho T., Lindfors C., Oasmaa A. (2024). Catalytic
Pyrolysis of a Model Plastic Mixture with a Montmorillonite Clay Catalyst
and Upgrading via Distillation. Energy Fuels.

[130] Cai W., Zhu X., Kumar R., Zhu Z., Ye J., Zhao J. (2024). Catalytic Pyrolysis of Biomass Waste
Using Montmorillonite-Supported
Ultrafine Iron Nanoparticles for Enhanced Bio-Oil Yield and Quality. Green Energy and Resources.

[131] Altalhi A. A., Mohamed E. A., Morsy S. M., Abou
Kana M. T. H., Negm N. A. (2021). Catalytic Manufacture and Characteristic
Valuation of Biodiesel-Biojet Achieved from Jatropha Curcas and Waste
Cooking Oils over Chemically Modified Montmorillonite Clay. J. Mol. Liq..

[132] Veses A., Aznar M., López J. M., Callén M. S., Murillo R., García T. (2015). Production
of Upgraded Bio-Oils by Biomass Catalytic Pyrolysis in an Auger Reactor
Using Low Cost Materials. Fuel.

[133] Abnisa F. (2023). Enhanced Liquid Fuel Production from Pyrolysis of Plastic
Waste Mixtures Using a Natural Mineral Catalyst. Energies (Basel).

[134] Lee S. Y., Yoon J. H., Kim J. R., Park D. W. (2001). Catalytic
Degradation of Polystyrene over Natural Clinoptilolite Zeolite. Polym. Degrad. Stab..

[135] Rocha M. V., Vinuesa A. J., Pierella L. B., Renzini M. S. (2020). Enhancement
of Bio-Oil Obtained from Co-Pyrolysis of Lignocellulose Biomass and
LDPE by Using a Natural Zeolite. Therm. Sci.
Eng. Prog..

[136] Bautista A. S., Rivera K. N. O., Suratos T. A. K. M., Dimaano M. N. R. (2024). Conversion of Polypropylene (PP)
Plastic Waste to Liquid
Oil through Catalytic Pyrolysis Using Philippine Natural Zeolite. IOP Conf Ser Mater Sci Eng.

[137] Bani G. A., Bani M. D. (2023). Ende Natural Zeolite
as a Catalyst
in the Biodiesel Production from Nyamplung Oil. International Journal of Basic and Applied Science.

[138] Lynn C. J., Dhir R. K., Ghataora G. S. (2017). Municipal
Incinerated
Bottom Ash Use as a Cement Component in Concrete. Mag. Concr. Res..

[139] Ahamed A., Liang L., Chan W. P., Tan P. C. K., Yip N. T. X., Bobacka J., Veksha A., Yin K., Lisak G. (2021). In Situ Catalytic Reforming of Plastic Pyrolysis Vapors Using MSW
Incineration Ashes. Environ. Pollut..

[140] Ashok J., Das S., Yeo T. Y., Dewangan N., Kawi S. (2018). Incinerator Bottom Ash Derived from Municipal Solid Waste as a Potential
Catalytic Support for Biomass Tar Reforming. Waste Manage..

[141] Liu B., You C., Wang H. (2024). Ni-FeOx Synergy
Induced by Metal-Support
Interaction on Ni-Impregnated Incineration Bottom Ash for Effective
Tar Reforming. Waste Manage..

[142] Al-Rahbi A. S., Williams P. T. (2019). Waste Ashes as Catalysts for the
Pyrolysis-Catalytic Steam Reforming of Biomass for Hydrogen-Rich Gas
Production. J. Mater. Cycles Waste Manage..

[143] Yu G., Chen D., Arena U., Huang Z., Dai X. (2018). Reforming
Sewage Sludge Pyrolysis Volatile with Fe-Embedded Char: Minimization
of Liquid Product Yield. Waste Manage..

[144] Wang S., Shan R., Gu J., Zhang J., Yuan H., Chen Y. (2020). Pyrolysis Municipal
Sludge Char Supported
Fe/Ni Catalysts for Catalytic Reforming of Tar Model Compound. Fuel.

[145] Lu J., Veksha A., Lisak G. (2024). Conversion
of Municipal Sewage Sludge
into Biogenic Multi-Walled Carbon Nanotubes and Hydrogen Using X-Mo/MgO
(X = Co, Fe, Ni) Catalysts through Pyrolysis-Chemical Vapor Deposition
Process. Chemical Engineering Journal.

[146] Ligero A., Calero M., Pérez A., Solís R. R., Muñoz-Batista M. J., Martín-Lara M. (2023). Á.
Low-Cost Activated Carbon from the Pyrolysis of Post-Consumer Plastic
Waste and the Application in CO2 Capture. Process
Saf. Environ. Prot..

[147] Martín-Lara M.
A., Piñar A., Ligero A., Blázquez G., Calero M. (2021). Characterization and
Use of Char Produced from Pyrolysis of Post-Consumer Mixed Plastic
Waste. Water (Basel).

[148] Garcia L., Cordoba M., Dosso L., Vera C., Busto M., Badano J. (2022). Catalytic Steam Reforming of Biomass
Tar Model Compounds with Low Cost Catalysts: Effect of Operation Conditions. Top. Catal..

[149] Khaliq A., Rhamdhani M., Brooks G., Masood S. (2014). Metal Extraction
Processes for Electronic Waste and Existing Industrial Routes: A Review
and Australian Perspective. Resources.

[150] Gift M. D. M., Verma S., Prasad K., Kathiresan K., Prasad R., Logeswaran T., Ghotekar S., Thao D. V., Lalvani J. I. J. (2022). Green Catalytic
Pyrolysis: An Eco-Friendly Route for
the Production of Fuels and Chemicals by Blending Oil Industry Wastes
and Waste Furniture Wood. J. Nanomater..

[151] Hart A., Omajali J. B., Murray A. J., MacAskie L. E., Greaves M., Wood J. (2016). Comparison of the Effects of Dispersed
Noble Metal (Pd) Biomass Supported Catalysts with Typical Hydrogenation
(Pd/C, Pd/Al2O3) and Hydrotreatment Catalysts (CoMo/Al2O3) for in-Situ
Heavy Oil Upgrading with Toe-to-Heel Air Injection (THAI). Fuel.

[152] Omajali J. B., Hart A., Walker M., Wood J., Macaskie L. E. (2017). In-Situ Catalytic Upgrading of Heavy
Oil Using Dispersed
Bionanoparticles Supported on Gram-Positive and Gram-Negative Bacteria. Appl. Catal., B.

[153] Areeprasert C., Khaobang C. (2018). Pyrolysis and Catalytic
Reforming
of ABS/PC and PCB Using Biochar and e-Waste Char as Alternative Green
Catalysts for Oil and Metal Recovery. Fuel Process.
Technol..

[154] Rafiee E., Khodayari M., Kahrizi M., Tayebee R. (2012). H5CoW12O40
Supported on Nano Silica from Rice Husk Ash: A Green Bifunctional
Catalyst for the Reaction of Alcohols with Cyclic and Acyclic 1,3-Dicarbonyl
Compounds. J Mol Catal A Chem.

[155] Pham L. K. H., Alsaiari M., Thao B. T. T., Hieu N. H., Phuc Hoang Duy N., Vo D.-V. N., Witoon T., Nguyen V. C., Kongparakul S., Samart C., Trinh T. H., Al-Gheethi A. (2024). High Selective
Hydrocarbon and Hydrogen Products from Catalytic Pyrolysis of Rice
Husk: Role of the Ordered Mesoporous Silica Derived from Rice Husk
Ash for Ni-Nanocatalyst Performance. J. Anal.
Appl. Pyrolysis.

[156] Sutrisno B., Hidayat A. (2016). Upgrading of Bio-Oil
from the Pyrolysis
of Biomass over the Rice Husk Ash Catalysts. IOP Conf Ser Mater Sci Eng.

[157] Zou R., Qian M., Wang C., Mateo W., Wang Y., Dai L., Lin X., Zhao Y., Huo E., Wang L., Zhang X., Kong X., Ruan R., Lei H. (2022). Biochar: From
by-Products of Agro-Industrial Lignocellulosic Waste to Tailored Carbon-Based
Catalysts for Biomass Thermochemical Conversions. Chemical Engineering Journal.

[158] Mudi I., Hart A., Ingram A., Wood J. (2023). Catalytic
Hydrodeoxygenation of Vanillin, a Bio-Oil Model Compound over Renewable
Ni/Biochar Catalyst. Catalysts.

[159] An Y., Tahmasebi A., Zhao X., Matamba T., Yu J. (2020). Catalytic
Reforming of Palm Kernel Shell Microwave Pyrolysis Vapors over Iron-Loaded
Activated Carbon: Enhanced Production of Phenol and Hydrogen. Bioresour. Technol..

[160] Malek N. H., Syed-Hassan S. S. A., Zhang S., Alias R., Mohamad Daud A. R. (2022). Hydrogen-
and Methane-Rich Clean Producer Gas from
the Reforming of Bio-Oil with Fe/AC Catalyst Prepared by a Stepwise
Impregnation Method. Bioenergy Res..

[161] Chen, G. ; Li, N. ; Cheng, Z. Solid Waste-Based Materials for Environmental Remediation; CRC Press: New York, 2024.

[162] Bekele D. T., Shibeshi N. T., Reshad A. S. (2022). KNO 3 -Loaded Coffee
Husk Ash as a Heterogeneous Alkali Catalyst for Waste Frying Oil Valorization
into Biodiesel. ACS Omega.

[163] Afessa M. M., Olu F. E., Geleta W. S., Legese S. S., Ramayya A. V. (2025). Unlocking the Potential of Biochar Derived from Coffee
Husk and Khat Stem for Catalytic Tar Cracking during Biomass Pyrolysis:
Characterization and Evaluation. Biomass Convers.
Biorefin..

[164] Fernández J. V., Faria D. N., Santoro M. C., Mantovaneli R., Cipriano D. F., Brito G. M., Carneiro M. T. W. D., Schettino M. A., Gonzalez J. L., Freitas J. C. C. (2023). Use of Unmodified
Coffee Husk Biochar and Ashes as Heterogeneous Catalysts in Biodiesel
Synthesis. Bioenergy Res..

[165] Daimary N., Boruah P., Eldiehy K. S. H., Pegu T., Bardhan P., Bora U., Mandal M., Deka D. (2022). Musa Acuminata
Peel: A Bioresource for Bio-Oil and by-Product Utilization as a Sustainable
Source of Renewable Green Catalyst for Biodiesel Production. Renew Energy.

[166] Hart A. (2020). Mini-Review of Waste Shell-Derived
Materials’ Applications. Waste Management
and Research.

[167] Rasaq W. A., Okpala C. O. R., Igwegbe C. A., Białowiec A. (2024). Catalyst-Enhancing
Hydrothermal Carbonization of Biomass for Hydrochar and Liquid Fuel
Production—A Review. Materials.

[168] Xiong J., Zhang S., Fan L., Zhang Q., Cui X., Ke L., Zeng Y., Wu Q., Cobb K., Liu Y., Ruan R., Wang Y. (2023). Production of Bio-Oil from Waste
Cooking Oil via Microwave-Assisted Pyrolysis in the Presence of Waste
Eggshell CaO and HZSM-5: Process Optimization and Catalyst Lifetime
Exploration. Energy.

[169] Tran X. T., Kim E. S., Mun D. H., Jung T., Shin J., Kang N. Y., Park Y.-K., Kim D. K. (2024). Catalytic
Cracking of Crude Waste Plastic Pyrolysis Oil for Enhanced Light Olefin
Production in a Pilot-Scale Circulating Fluidized Bed Reactor. ACS Sustain Chem Eng.

[170] Catizane C., Jiang Y., Sumner J. (2024). Improving Plastic Pyrolysis
Oil Quality via an Electrochemical Process for Polymer Recycling:
A Review. Energy Adv..

[171] Mangesh V. L., Padmanabhan S., Tamizhdurai P., Ramesh A. (2020). Experimental Investigation to Identify the Type of
Waste Plastic Pyrolysis Oil Suitable for Conversion to Diesel Engine
Fuel. J Clean Prod.

[172] Serras-Malillos A., Perez-Martinez B. B., Iriondo A., Acha E., Lopez-Urionabarrenechea A., Caballero B. M. (2024). Quantification
of the Composition of Pyrolysis Oils of Complex Plastic Waste by Gas
Chromatography Coupled with Mass Spectrometer Detector. RSC Adv..

[173] Fekhar B., Gombor L., Miskolczi N. (2019). Pyrolysis
of Chlorine Contaminated Municipal Plastic Waste: In-Situ Upgrading
of Pyrolysis Oils by Ni/ZSM-5, Ni/SAPO-11, Red Mud and Ca­(OH)­2 Containing
Catalysts. J. Energy Inst..

[174] Fekhar B., Zsinka V., Miskolczi N. (2019). Value Added
Hydrocarbons Obtained by Pyrolysis of Contaminated Waste Plastics
in Horizontal Tubular Reactor: In Situ Upgrading of the Products by
Chlorine Capture. J Clean Prod.

[175] Mase C., Maillard J. F., Paupy B., Hubert-Roux M., Afonso C., Giusti P. (2022). Speciation and Semiquantification
of Nitrogen-Containing Species in Complex Mixtures: Application to
Plastic Pyrolysis Oil. ACS Omega.

[176] Belbessai S., Azara A., Abatzoglou N. (2022). Recent Advances
in the Decontamination and Upgrading of Waste Plastic Pyrolysis Products:
An Overview. Processes.

[177] Budsaereechai S., Hunt A. J., Ngernyen Y. (2019). Catalytic Pyrolysis
of Plastic Waste for the Production of Liquid Fuels for Engines. RSC Adv..

[178] Beccaria M., Piparo M., Zou Y., Stefanuto P. H., Purcaro G., Mendes Siqueira A. L., Maniquet A., Giusti P., Focant J. F. (2023). Analysis of Mixed Plastic Pyrolysis Oil by Comprehensive
Two-Dimensional Gas Chromatography Coupled with Low- and High-Resolution
Time-of-Flight Mass Spectrometry with the Support of Soft Ionization. Talanta.

[179] Lingaiah N., Azhar Uddin Md., Muto A., Sakata Y., Imai T., Murata K. (2001). Catalytic
Dechlorination of Chloroorganic
Compounds from PVC-Containing Mixed Plastic-Derived Oil. Appl Catal A Gen.

[180] Liu M., Wang W., Zhang Q., Liu Z., Li Y., Wu M., Zhang D. (2023). Detailed Characterization of Chlorides
and Oxides in
Plastic Waste Pyrolysis Oil from Paper Mills by GC-ECD and GC-MS with
Solid Phase Extraction. Energy Fuels.

[181] Kusenberg M., Eschenbacher A., Djokic M. R., Zayoud A., Ragaert K., De Meester S., Van Geem K. M. (2022). Opportunities and
Challenges for the Application of Post-Consumer Plastic Waste Pyrolysis
Oils as Steam Cracker Feedstocks: To Decontaminate or Not to Decontaminate?. Waste Manage..

[182] Serras-Malillos A., Perez-Martinez B. B., Iriondo A., Acha E., Lopez-Urionabarrenechea A., Caballero B. M. (2024). Quantification
of the Composition of Pyrolysis Oils of Complex Plastic Waste by Gas
Chromatography Coupled with Mass Spectrometer Detector. RSC Adv..

[183] Dao
Thi H., Djokic M. R., Van Geem K. M. (2021). Detailed Group-Type
Characterization of Plastic-Waste Pyrolysis Oils: By Comprehensive
Two-Dimensional Gas Chromatography Including Linear, Branched, and
Di-Olefins. Separations.

[184] Pumpuang A., Klinkaew N., Wathakit K., Sukhom A., Sukjit E. (2024). The Influence of Plastic Pyrolysis Oil on Fuel Lubricity
and Diesel Engine Performance. RSC Adv..

[185] Netsch N., Weigel L., Schmedding T., Zeller M., Bergfeldt B., Straczewski G., Tavakkol S., Stapf D. (2025). Chemical Characterization of Mixed
Plastic Pyrolysis Oils Relevant for Cracker Reintegration by Advanced
Two-Dimensional Gas Chromatography. Fuel Process.
Technol..

[186] Sharuddin S. D. A., Abnisa F., Daud W. M. A. W., Aroua M. K. (2018). Pyrolysis of Plastic Waste for Liquid Fuel Production
as Prospective Energy Resource. IOP Conf Ser
Mater Sci Eng.

[187] Fan L., Zhang Y., Liu S., Zhou N., Chen P., Liu Y., Wang Y., Peng P., Cheng Y., Addy M., Lei H., Ruan R. (2017). Ex-Situ Catalytic Upgrading of Vapors from Microwave-Assisted
Pyrolysis of Low-Density Polyethylene with MgO. Energy Convers Manag.

[188] Onwudili J. A., Muhammad C., Williams P. T. (2019). Influence
of Catalyst
Bed Temperature and Properties of Zeolite Catalysts on Pyrolysis-Catalysis
of a Simulated Mixed Plastics Sample for the Production of Upgraded
Fuels and Chemicals. J. Energy Inst..

[189] Brown J. L., Radhakrishnan H., Coffman I., Tumu K., Curtzwiler G., Vorst K., Smith R. G., Bai X., Daugaard T. J. (2024). Catalytic
Upgrading of Pyrolysis Condensables from
Postconsumer Polyolefins Using HZSM-5. Energy
Fuels.

[190] Wang S., Kim H., Lee D., Lee Y.-R., Won Y., Hwang B. W., Nam H., Ryu H.-J., Lee K.-H. (2021). Drop-in
Fuel Production with Plastic Waste Pyrolysis Oil over Catalytic Separation. Fuel.

[191] Lv Y., Wang X., Gao D., Ma X., Li S., Wang Y., Song G., Duan A., Chen G. (2018). Hierarchically
Porous ZSM-5/SBA-15 Zeolite: Tuning Pore Structure and Acidity for
Enhanced Hydro-Upgrading of FCC Gasoline. Ind.
Eng. Chem. Res..

[192] Chen W. H., Pratim Biswas P., Kwon E. E., Park Y. K., Rajendran S., Gnanasekaran L., Chang J. S. (2023). Optimization of
the Process Parameters of Catalytic Plastic Pyrolysis for Oil Production
Using Design of Experiment Approaches: A Review. Chemical Engineering Journal.

[193] Han C., Yang J., Dong S., Ma L., Dai Q., Guo J. (2025). Zeolite Preparation from Industrial
Solid Waste: Current Status,
Applications, and Prospects. Sep. Purif. Technol..

[194] Eldeeb A. M., Aboutaleb W. A., Mohamed R. S., Dhmees A. S., Ahmed A. I. (2022). Gasoline and Diesel-like Fuel Production via Hydrocracking
of Hydrotreated Tire Pyrolytic Oil over Ni-W/MCM-41 Derived from Blast
Furnace Slag. J. Energy Inst..

[195] Briones L., Cordero A., Alonso-Doncel M., Serrano D. P., Escola J. M. (2024). Catalytic Upgrading of a Model Polyethylene
Pyrolysis Oil by Hydroconversion over Ni-Containing Hierarchical Beta
Zeolites with Tailored Acidity. Appl. Catal.,
B.

[196] Hwang K.-R., Choi S.-A., Choi I.-H., Lee K.-H. (2021). Catalytic
Cracking of Chlorinated Heavy Wax from Pyrolysis of Plastic Wastes
to Low Carbon-Range Fuels: Catalyst Effect on Properties of Liquid
Products and Dechlorination. J. Anal. Appl.
Pyrolysis.

[197] Gancedo J., Li H., Walz J. S., Faba L., Ordoñez S., Huber G. W. (2024). Investigation into
the Shape Selectivity
of Zeolites for Conversion of Polyolefin Plastic Pyrolysis Oil Model
Compound. Appl Catal A Gen.

[198] Villalobos M., Awojulu A., Greeley T., Turco G., Deeter G. (2006). Oligomeric
Chain Extenders for Economic Reprocessing
and Recycling of Condensation Plastics. Energy.

[199] Padmanabhan S., Giridharan K., Stalin B., Kumaran S., Kavimani V., Nagaprasad N., Tesfaye Jule L., Krishnaraj R. (2022). Energy Recovery of Waste Plastics
into Diesel Fuel
with Ethanol and Ethoxy Ethyl Acetate Additives on Circular Economy
Strategy. Sci Rep.

[200] Park S. J., Kang S. H., Hwang J. G., Kim H. S., Seok J., Choi M., Choi Y. H., Lee J. W. (2025). Upgrading
of Actual Pyrolysis Oil Derived from Waste Plastics through Catalytic
Cracking of Chlorinated Heavy Fractions Using Alumina-Supported Tungsten
Oxide. Mol. Catal..

[201] Zhang R., Deng G., Jiang Z., Fan Y., Guo Y., Dong Z., Chen W., Peng B., Zhang F. (2025). Upgrading
Polyolefin Plastics: Experiences from Petroleum Refining and Distinct
Characteristics. Sci China Chem.

[202] Komandur J., Chaudhary V., Vinu R., Mohanty K. (2025). Red Mud-Supported
Ni and Co Catalysts for Hydrodeoxygenation of Palmitic Acid: Insights
into Reaction Pathway, Product Selectivity, and Catalyst Recyclability. Energy Fuels.

[203] Zhang S., Xin B. (2025). Rapid Regeneration
of Spent FCC Catalysts
through Selective Bioleaching by the Spent Medium Process. ACS Omega.

[204] Vollmer I., Jenks M. J. F., Mayorga González R., Meirer F., Weckhuysen B. M. (2021). Plastic Waste Conversion over a Refinery
Waste Catalyst. Angew. Chem., Int. Ed..

[205] Wang P., Qiao L., Wang W., Yu J. (2023). Catalytic
Pyrolysis of Waste Composite Plastics with Waste FCC Catalyst. J. Energy Inst..

[206] Palmay P., Medina C., Donoso C., Barzallo D., Bruno J. C. (2023). Catalytic Pyrolysis of Recycled Polypropylene
Using
a Regenerated FCC Catalyst. Clean Technol. Environ.
Policy.

[207] Shan T., Wang K., Bian H., Wang C., Tian X. (2023). Study on Kinetics
of Spent FCC Catalyst Applied to the Living Waste
Plastics. Polym. Eng. Sci..

[208] Xu Q., Zhu J., Wu B., Jin G., Liu Y., Huang A., Tian C., Luo Y. (2024). Enhancing Light Fuel
Production through Catalytic Pyrolysis of Municipal Mixed Plastic
Waste over Activated Spent FCC Catalyst. J.
Energy Inst..

[209] Lindfors C., Khan M., Siddiq F., Arnold M., Ohra-aho T. (2024). Catalytic Processing of Mixed Plastics
Aiming for Industrial
Reuse. Energy Fuels.

[210] Heng J. Z. X., Tan T. T. Y., Xing Z., Ong J. L. Y., Lin K. S., Koh X. Q., Jiang W., Zhang L., Zhu Q., Li Z., Loh X. J., Lim J. Y. C., Ye E. (2023). Unraveling
the Catalytic Activity of CaClOH-Rich Incineration Fly Ash in the
Pyrolysis of Single-Use Plastics. Mater. Today
Chem..

[211] Premkumar P., Saravanan C. G., Nalluri P., Seeman M., Vikneswaran M., Madheswaran D. K., Femilda Josephin J., Chinnathambi A., Pugazhendhi A., Varuvel E. G. (2024). Production of Liquid
Hydrocarbon Fuels through Catalytic Cracking of High and Low-Density
Polyethylene Medical Wastes Using Fly Ash as a Catalyst. Process Saf. Environ. Prot..

[212] Zhang Z., Cheng Q., Shan C., Jiang Y., Kong G., Zhang G., Gopakumar S. T., Shi S., Zhang X., Han L. (2024). Catalytic Fast Pyrolysis over Heavy
Metals-Containing Livestock Manure Biochar Catalyst for Polystyrene
Upcycling. J. Anal. Appl. Pyrolysis.

[213] Yanik J., Uddin Md. A., Ikeuchi K., Sakata Y. (2001). The Catalytic
Effect of Red Mud on the Degradation of Poly (Vinyl Chloride) Containing
Polymer Mixture into Fuel Oil. Polym. Degrad.
Stab..

[214] Han T. U., Park Y.-K., Kim Y.-M. (2021). High-Quality
Oil
Production via the Catalytic Conversion of Printed Circuit Boards. J Clean Prod.

[215] Kjønli, A. Gas Dechlorination of Plastic Pyrolysis Derived Oil with Model Compounds Using Fe Based Material. Master’s thesis, Norwegian University of Science and Technology, 2023.

[216] Lingaiah N., Uddin Md. A., Muto A., Imai T., Sakata Y. (2001). Removal of Organic Chlorine Compounds
by Catalytic
Dehydrochlorination for the Refinement of Municipal Waste Plastic
Derived Oil. Fuel.

[217] López A., de Marco I., Caballero B. M., Laresgoiti M. F., Adrados A., Aranzabal A. (2011). Catalytic
Pyrolysis of Plastic Wastes with Two Different Types of Catalysts:
ZSM-5 Zeolite and Red Mud. Appl. Catal., B.

[218] Okino J., Siagi Z., Kumar A., Talai S., Muliwa A., Olomo E., Manirambona E. (2025). Thermal and
Catalytic Pyrolysis of Waste Plastic Heavy Distillate into Diesel-like
Product. Applications in Energy and Combustion
Science.

[219] Anil H., Cakici A. I., Yanik J., Uçar S., Karayildirim T. (2004). Utilization of Red Mud as Catalyst
in Conversion of
Waste Oil and Waste Plastics to Fuel. J. Mater.
Cycles Waste Manage..

[220] Jang J. J., Yoon Y. M., Choi Y., Nam H., Lee D., Kim H., Won Y., Kim D., Ryu H.-J., Bae J. W., Hwang B. (2025). Resource-Efficient Upgrading of Plastic
Pyrolysis Oil: Nickel-Loaded Kaolin for Enhancing Light Oil Yield
at Lower Temperatures. ACS Sustain Chem Eng.

[221] Jahromi H., Agblevor F. A. (2018). Hydrodeoxygenation of Pinyon-Juniper
Catalytic Pyrolysis Oil Using Red Mud-Supported Nickel Catalysts. Appl. Catal., B.

[222] Anil H., Cakici A. I., Yanik J., UΔar S., Karayildirim T. (2004). Utilization of Red Mud as Catalyst
in Conversion of
Waste Oil and Waste Plastics to Fuel. J. Mater.
Cycles Waste Manage..

[223] Kim H. S., Kim H.-J., Kim J., Kim J.-H., Kang T.-J., Kang S.-H., Lee Y., Lee S. C., Chang C.-S., Bae J. W. (2025). Research on the Chlorine Removal
and Upgrading of Waste Plastic Pyrolysis Oil Using Iron-Based Adsorbents. Energies (Basel).

[224] Zhuang Y., Wang X., Shah K. J., Sun Y. (2025). A Review on
the Preparation of Catalysts Using Red Mud Resources. Catalysts.

[225] Zhou X., Zhang L., Chen Q., Xiao X., Wang T., Cheng S., Li J. (2023). Study on the
Mechanism
and Reaction Characteristics of Red-Mud-Catalyzed Pyrolysis of Corn
Stover. Fuel.

[226] Sekizkardeş B., Soyer-Uzun S., Uzun A., Kuhn S., Kaya-Özkiper K., Kurtoğlu-Öztulum S. F. (2025). A Comprehensive
Review on Red Mud-Based Catalysts: Modification Methods and Applications
in Thermal- and Photocatalysis. ChemCatChem.

[227] Kasataka K., Umemoto S., Ikeda A. (2025). Potential
Use of Biochar
as a Catalyst and an Adsorbent in Plastic Pyrolysis Process. Waste Biomass Valorization.

[228] de Freitas Costa A., Ferreira C., da Paz S., Santos M., Moreira L., Mendonça N., da Costa Assunção F., de Freitas A., Costa R., de Sousa Brandão I., da Costa C., da Mota S., de Castro D., Duvoisin S., Borges L., Machado N., Bernar L. (2023). Catalytic
Upgrading of Plastic Waste of Electric and Electronic Equipment (WEEE)
Pyrolysis Vapors over Si-Al Ash Pellets in a Two-Stage Reactor. Energies (Basel).

[229] Das B., Mohanty K. (2019). A Review on Advances
in Sustainable Energy Production
through Various Catalytic Processes by Using Catalysts Derived from
Waste Red Mud. Renew Energy.

[230] Hart A., Patel H., Yildirir E., Onwudili J. A. (2025). Influence
of Surface Acidity/Basicity of Selected Metal Oxide Catalysts and
Reaction Atmospheres on the Ketonisation of Propionic Acid to Produce
3-Pentanone as a Liquid Biofuel Precursor. Renew
Energy.

[231] Watanabe M. D. B., Cherubini F., Cavalett O. (2022). Climate Change Mitigation
of Drop-in Biofuels for Deep-Sea Shipping under a Prospective Life-Cycle
Assessment. J Clean Prod.

[232] Ansari K. B., Arora J. S., Chew J. W., Dauenhauer P. J., Mushrif S. H. (2019). Fast Pyrolysis of Cellulose, Hemicellulose, and Lignin:
Effect of Operating Temperature on Bio-Oil Yield and Composition and
Insights into the Intrinsic Pyrolysis Chemistry. Ind. Eng. Chem. Res..

[233] Tran Q. K., Le M. L., Ly H. V., Woo H. C., Kim J., Kim S.-S. (2021). Fast Pyrolysis of Pitch Pine Biomass in a Bubbling
Fluidized-Bed Reactor for Bio-Oil Production. Journal of Industrial and Engineering Chemistry.

[234] Oasmaa A., Czernik S. (1999). Fuel Oil Quality of Biomass Pyrolysis
OilsState of the Art for the End Users. Energy
Fuels.

[235] Das B., Mohanty K. (2020). Microwave Induced One-Pot
Conversion of D-Glucose to
5-Hydroxymethylfurfural by a Novel Sulfate-Functionalized Sn-Red Mud
Catalyst. Sustain Energy Fuels.

[236] Das, B. ; Mohanty, K. Progress on Red Mud-Based Catalysts for the Removal of Environmental Pollutants Through Oxidation and Advanced Oxidation Process. In Sustainable Green Chemical Processes and their Allied Applications. Nanotechnology in the Life Sciences; Inamuddin, A. , Ed.; Springer: Cham, 2020; pp 461–480.

[237] Das B., Ahmed A. M. A., Hill J. M., Ponnurangam S. (2023). Boron-Induced
Surface Defects in Petcoke as Active Centers for Aerobic Oxidative
Desulfurization. Energy Fuels.

[238] Rahman T., Jahromi H., Roy P., Adhikari S., Feyzbar-Khalkhali-Nejad F., Oh T.-S., Wang Q., Higgins B. T. (2023). Influence of Red Mud Catalyst and
Reaction Atmosphere
on Hydrothermal Liquefaction of Algae. Energies
(Basel).

[239] Chen J., Wang D., Luo F., Yang X., Li X., Li S., Ye Y., Wang D., Zheng Z. (2022). Selective
Production of Alkanes and Fatty Alcohol via Hydrodeoxygenation of
Palmitic Acid over Red Mud-Supported Nickel Catalysts. Fuel.

[240] Wang Q., Li X., Duan J., Chen J., Ye Y., Wang D., Li S., Zheng Z. (2022). Rationally Control
the Path of Hydrodeoxygenation of Palmitic Acid over Ni/Red-Mud Catalysts
by Surface Decoration of Oxophilic MoOx Species. Fuel.

[241] Duan J., Wu Y., Zheng J., Li X., Lin X., Wang D., Ye Y., Zheng Z. (2023). Enhancing
Catalytic
Performance of Red Mud for Palmitic Acid Hydrodeoxygenation by Acid
Pretreatment-Induced Structural Modification. Fuel Process. Technol..

[242] Wang Q., Chen J., Li X., Yang X., Wu Y., Li S., Ye Y., Wang D., Wang D., Zheng Z. (2022). Calcination Temperature
Induced Structural Change of Red Mud and
Its Enhanced Catalytic Performance for Hydrocarbon-Based Biofuels
Production. Fuel Process. Technol..

[243] Damizia M., Bracciale M. P., Mousavi S., Tai L., De Filippis P., de Caprariis B. (2024). Red Mud as Hydrogen Producer in Hydrothermal
Liquefaction of Pinewood: Minimization of Process Wastes by Recycling
the Water and Hydrochar Phases. Renew Energy.

[244] Duan J., Chen S., Yao Q., Wu K., Luo F., Wang D., Liu P., Jiang J., Zheng Z. (2025). Tailoring
Nickel Precursors on Red Mud Supports for Catalytic Hydrodeoxygenation
of Palmitic Acid towards Green Diesel Production. Renew Energy.

[245] Rana M., Park J.-H. (2025). One-Pot Catalytic
Hydrogenolysis
and Hydrogenation of Kraft Lignin to Highly Selective Catechols in
Subcritical Water over Co-Supported Activated Red Mud Catalyst. Biomass Bioenergy.

[246] Huan W., Zhu L., Wang J., Liu P., Liu B., Li M., Ma Z., Li J. (2024). Red Mud Catalysts
for
Hydrothermal Liquefaction of Alkali Lignin: Optimization of Reaction
Parameters. J. Energy Inst..

[247] Suchamalawong P., Pengnarapat S., Reubroycharoen P., Vitidsant T. (2019). Biofuel Preparation
from Waste Chicken Fat Using Coal
Fly Ash as a Catalyst: Optimization and Kinetics Study in a Batch
Reactor. J. Environ. Chem. Eng..

[248] Xie J.-X., Zhao Y.-P., Li Q., Qiu L.-L., Liu J.-C., Liu F.-J., Liang J., Cao J.-P. (2024). Application
of Coal-Based Waste Coal Gasification Slag for Catalytic Conversion
of Waste Lignin and Its Derivatives. Ind. Crops
Prod..

[249] Yu C., Jiang H., Shi J., Huang H., Fan L., Wen Z., Ai X., Que Z., Li Q., Xu C. C., Yang W. (2025). Steel Slag-Assisted
Hydrothermal Liquefaction of Hyperaccumulators
for Upgrading Bio-Oil and Immobilization of Arsenic. Fuel.

[250] Trisunaryanti W., Wijaya K., Triyono T., Adriani A. R., Larasati S. (2021). Green Synthesis of Hierarchical Porous
Carbon Prepared
from Coconut Lumber Sawdust as Ni-Based Catalyst Support for Hydrotreating
Callophyllum Inophyllum Oil. Results Eng..

[251] García-Rollán M., Bertran-Llorens S., Palazzolo M. A., Deuss P. J., Heeres H. J., Ruiz-Rosas R., Rosas J. M., Rodriguez-Mirasol J., Cordero T. (2025). Lignin Hydrotreatment
to Aromatics Products on Metallic Phosphides Carbon-Based Catalysts
Produced from Lignin. Fuel.

[252] Hamidi R., Tai L., Paglia L., Scarsella M., Damizia M., De Filippis P., Musivand S., de Caprariis B. (2022). Hydrotreating
of Oak Wood Bio-Crude Using Heterogeneous Hydrogen Producer over Y
Zeolite Catalyst Synthesized from Rice Husk. Energy Convers Manag.

[253] de la Puente G., Gil A., Pis J. J., Grange P. (1999). Effects of
Support Surface Chemistry in Hydrodeoxygenation Reactions over CoMo/Activated
Carbon Sulfided Catalysts. Langmuir.

[254] Rajesh M., Sau M., Malhotra R. K., Sharma D. K. (2016). Synthesis
and Characterization of Ni-Mo Catalyst Using *Jatropha Curcas* Leaves as Carbon Support and Its Catalytic Activity for Hydrotreating
of Gas Oil, Jatropha Oil, and Their Blends. Pet. Sci. Technol..

[255] Rajesh M., Sau M., Malhotra R. K., Sharma D. K. (2016). Synthesis
and Characterization of Ni-Mo Catalyst Using Pea Pod (*Pisum
Sativum L*) as Carbon Support and Its Hydrotreating Potential
for Gas Oil, Jatropha Oil, and Their Blends. Pet. Sci. Technol..

[256] Domínguez-Barroso M. V., Herrera C., Larrubia M. A., Alemany L. J. (2016). Diesel Oil-like Hydrocarbon Production from Vegetable
Oil in a Single Process over Pt-Ni/Al2O3 and Pd/C Combined Catalysts. Fuel Process. Technol..

[257] Roy P., Jahromi H., Adhikari S., Zou Finfrock Y., Rahman T., Ahmadi Z., Mahjouri-Samani M., Feyzbar-Khalkhali-Nejad F., Oh T.-S. (2022). Performance of Biochar
Assisted Catalysts during Hydroprocessing of Non-Edible Vegetable
Oil: Effect of Transition Metal Source on Catalytic Activity. Energy Convers Manag.

[258] Trisunaryanti W., Wijaya K., Triyono T., Wahyuningtyas N., Utami S. P., Larasati S. (2022). Characteristics of
Coconut Shell-Based
Activated Carbon as Ni and Pt Catalyst Supports for Hydrotreating
Calophyllum Inophyllum Oil into Hydrocarbon-Based Biofuel. J. Environ. Chem. Eng..

[259] Tran C.-C., Akmach D., Kaliaguine S. (2020). Hydrodeoxygenation
of Vegetable Oils over Biochar Supported Bimetallic Carbides for Producing
Renewable Diesel under Mild Conditions. Green
Chem..

[260] Roy P., Jahromi H., Rahman T., Baltrusaitis J., Hassan E. B., Torbert A., Adhikari S. (2023). Hydrotreatment of Pyrolysis
Bio-Oil with Non-Edible Carinata Oil and Poultry Fat for Producing
Transportation Fuels. Fuel Process. Technol..

[261] Kordouli E., Vourtsani P.-I., Mourgkogiannis N., Zafeiropoulos J., Bourikas K., Kordulis C. (2024). Green Diesel Production
Catalyzed by MoNi Catalysts Supported on Rice Husk Biochar. Catalysts.

[262] Cordero-Lanzac T., Rodríguez-Mirasol J., Cordero T., Bilbao J. (2021). Advances and Challenges in the Valorization
of Bio-Oil:
Hydrodeoxygenation Using Carbon-Supported Catalysts. Energy Fuels.

[263] Roy P., Jahromi H., Rahman T., Adhikari S., Feyzbar-Khalkhali-Nejad F., Barbary
Hassan E., Oh T.-S. (2022). Understanding the Effects of Feedstock
Blending and Catalyst Support on Hydrotreatment of Algae HTL Biocrude
with Non-Edible Vegetable Oil. Energy Convers
Manag.

[264] Liu C., Zhou C., Wang Y., Liu X., Zhu L., Ma H., Zhou Z., Qi F. (2021). Gas-Phase
Hydrodeoxygenation of Bio-Oil
Model Compound over Nitrogen-Doped Carbon-Supported Palladium Catalyst. Proc. Combust. Inst..

[265] Leal Mendes F., Teixeira da Silva V., Edral Pacheco M., de Rezende Pinho A., Assumpção Henriques C. (2020). Hydrotreating
of Fast Pyrolysis Oil: A Comparison of Carbons and Carbon-Covered
Alumina as Supports for Ni2P. Fuel.

[266] Ge F., Xia H., Li J., Xue Y., Su J., Yang X., Jiang J., Zhou M. (2023). Lignin Meets MOFs:
Lignin Derived Metal Carbon Material for the Catalytic Hydrotreatment
of Guaiacol to Cyclohexanol. Chemical Engineering
Journal.

[267] Yan S., Zhuang J., Liu X., Wu H., Guan Y., Zhu X., Yang F. (2024). Modification of ZSM-23
Zeolite via Vapor-Phase Transport
Method: Reducing the Length along the Unidimensional Channels for
Enhanced Catalytic Performances in Methanol-to-Hydrocarbons. Microporous Mesoporous Mater..

[268] Hao Q., Yang Z., Wu B., Zhu J., Li Z., Liu J., Ma L. (2022). Study on the Deactivation
of Ni-Based Catalyst in the
Hydrotreating Process of Waste Plastic Pyrolysis Oil. J. Anal. Appl. Pyrolysis.

[269] Murthy K. (2004). An Exploration of Activity Loss during
Hydrodechlorination
and Hydrodebromination over Ni/SiO2. J. Catal..

[270] Kim P., Kim Y., Kim H., Song I. K., Yi J. (2005). Preparation,
Characterization, and Catalytic Activity of NiMg Catalysts Supported
on Mesoporous Alumina for Hydrodechlorination of o-Dichlorobenzene. J Mol Catal A Chem.

[271] Chen J., Ci D., Yang Q., Li K. (2014). Deactivation
of Ni 2 P/SiO 2 Catalyst in Hydrodechlorination of Chlorobenzene. Appl. Surf. Sci..

[272] Ding Z., Zhu R., Xie Q., Long S., Fu H., Chen Q., Xi Y. (2024). Effects of
Iron Oxide Minerals on
the Pyrolysis of Polyvinyl Chloride: Catalytic Dechlorination, Chlorine
Capture, and Carbon Sequestration. J Clean Prod.

[273] Kim T.-Y., Hong S.-H., Kim J.-C., Jang H.-W., Lee Y., Kim H.-J., Lee S.-C., Kang S.-H. (2024). Chlorine Gas Removal
by H2 Treated Red Mud for the Potential Application in Waste Plastic
Pyrolysis Process. Sustainability.

[274] Liu Y., Li Y., Zhou F., Hu Y., Zhang Y. (2016). Sulfur Fixation
by Chemically Modified Red Mud Samples Containing Inorganic Additives:
A Parametric Study. Adv. Mater. Sci. Eng..

[275] Lee S., Eun Lee J., Jong Lee S., Wook Lee J., Yun Y., Park N.-K., Kang D., Kim M. (2024). Synergistic Desulfurization
Performance of Industrial Waste Red Mud: A Comprehensive Experimental
and Computational Study for COS Removal and CO­(g) Production. Appl. Surf. Sci..

[276] Romero A., Moreno I., Escudero L., Yuste R., Pizarro P., Moreno J. M., Serrano D. P. (2024). Dechlorination
of
a Real Plastic Waste Pyrolysis Oil by Adsorption with Zeolites. J. Environ. Chem. Eng..

[277] Nganda A., Srivastava P., Lamba B. Y., Pandey A., Kumar M. (2023). Advances in
the Fabrication, Modification, and Performance of Biochar,
Red Mud, Calcium Oxide, and Bentonite Catalysts in Waste-to-Fuel Conversion. Environ. Res..

[278] Tan K. Q., Ahmad M. A., Oh W. D., Low S. C. (2023). Valorization
of Hazardous Plastic Wastes into Value-Added Resources by Catalytic
Pyrolysis-Gasification: A Review of Techno-Economic Analysis. Renewable and Sustainable Energy Reviews.

[279] Aminu I., Nahil M. A., Williams P. T. (2023). Pyrolysis-Plasma/Catalytic
Reforming of Post-Consumer Waste Plastics for Hydrogen Production. Catal. Today.

